# Advanced EXAFS analysis techniques applied to the *L*-edges of the lanthanide oxides

**DOI:** 10.1107/S1600576724010240

**Published:** 2024-11-22

**Authors:** Adam Smerigan, Adam S. Hoffman, Lars Ostervold, Jiyun Hong, Jorge Perez-Aguillar, Ash C. Caine, Lauren Greenlee, Simon R. Bare

**Affiliations:** a Department of Chemical Engineering Pennsylvania State University University Park PA USA; b Stanford Synchrotron Radiation Laboratory SLAC National Accelerator Laboratory Menlo Park CA94025 USA; c SUNCAT Center for Interface Science and Catalysis SLAC National Accelerator Laboratory Menlo Park CA94025 USA; d Department of Mechanical Engineering Washington University in St Louis St Louis MO63130 USA; Montanuniversität Leoben, Austria

**Keywords:** extended X-ray absorption fine structure, EXAFS, rare earth elements, lanthanide oxides, multielectron excitation, cubic crystal expansion, non-cubic crystal expansion

## Abstract

The unique properties of the lanthanide (Ln) elements make them critical components of modern technologies, such as lasers, anti-corrosive films and catalysts. Thus, there is significant interest in establishing structure–property relationships for Ln-containing materials to advance these technologies. Extended X-ray absorption fine structure (EXAFS) is an excellent technique for this task considering its ability to determine the average local structure around the Ln atoms for both crystalline and amorphous materials. However, the limited availability of EXAFS reference spectra of the Ln oxides and challenges in the EXAFS analysis have hindered the application of this technique to these elements. The challenges include the limited *k*-range available for the analysis due to the superposition of *L*-edges on the EXAFS, multielectron excitations (MEEs) creating erroneous peaks in the EXAFS and the presence of inequivalent absorption sites. Herein, we removed MEEs to model the local atomic environment more accurately for light Ln oxides. Further, we investigated the use of cubic and non-cubic lattice expansion to minimize the fitting parameters needed and connect the fitting parameters to physically meaningful crystal parameters. The cubic expansion reduced the number of fitting parameters but resulted in a statistically worse fit. The non-cubic expansion resulted in a similar quality fit and showed non-isotropic expansion in the crystal lattice of Nd_2_O_3_. In total, the EXAFS spectra and the fits for the entire set of Ln oxides (excluding promethium) are included. The knowledge developed here can assist in the structural determination of a wide variety of Ln compounds and can further studies on their structure–property relationships.

## Abstract 2

The *L*-edge extended X-ray absorption fine structure (EXAFS) spectra of the entire set of lanthanide oxides were collected and modeled, taking into consideration the aggregation of inequivalent absorbing sites, geometric parameterization of the crystal lattice and multielectron excitation removal.

## 1. Introduction

There is much interest in determining the local structure of lanthanides within catalysts (Romanchuk *et al.*, 2022 ▸; Fonda *et al.*, 1999 ▸; Ebitani *et al.*, 2001 ▸; Huang *et al.*, 2017 ▸), laser materials (Karabulut *et al.*, 2002 ▸; Peters & Houde-Walter, 1998 ▸), anti-corrosive films (Abuín *et al.*, 2012 ▸; Shahin *et al.*, 2005 ▸) and others (Schlegel *et al.*, 2004 ▸; Lu *et al.*, 2014 ▸; Yang *et al.*, 2014 ▸) due to the lanthanide (Ln) elements having unique properties (*e.g.* photoluminescence, magnetism, reactivity) that make their inclusion into a diverse array of materials beneficial. However, detailed information about their local bonding environment is required to optimize their integration (Erol *et al.*, 2021 ▸; Pan *et al.*, 2022 ▸). Since compounds containing lanthanides have varying levels of crystallinity, extended X-ray absorption fine structure (EXAFS) is an ideal method for determining the local structure of these elements (Malet *et al.*, 1994 ▸). The EXAFS spectra of Ln oxides have been used to confirm the incorporation and the phase of lanthanides in materials (Abuín *et al.*, 2012 ▸; Sun *et al.*, 2022 ▸). Studies have investigated how the coordination number (CN) and disorder of Ln oxides incorporated in glasses affect the photoluminescent properties [*i.e.* broadening of emission bands (Karabulut *et al.*, 2002 ▸; Peters & Houde-Walter, 1998 ▸)]. Others have compared the CNs of Ln atoms in bulk oxides with those of nanoparticles to determine their average size (Romanchuk *et al.*, 2022 ▸).

However, the availability of high-quality Ln oxide EXAFS spectra and EXAFS fits is limited for the *L*-edge of many of the Ln oxides. The EXAFS spectra and fits of La_2_O_3_ (Lu *et al.*, 2014 ▸; Malet *et al.*, 1994 ▸), CeO_2_ (Fonda *et al.*, 1999 ▸; Shahin *et al.*, 2005 ▸; Romanchuk *et al.*, 2022 ▸), Nd_2_O_3_ (Fabian *et al.*, 2021 ▸), Sm_2_O_3_ (Malet *et al.*, 1994 ▸), Eu_2_O_3_ (Schlegel *et al.*, 2004 ▸) and Er_2_O_3_ (Peters & Houde-Walter, 1998 ▸) are available. Yet EXAFS spectra of other Ln oxides are not available in the literature, or lack phase confirmation or modeling of the data to validate the structure (Huang *et al.*, 2017 ▸; Abuín *et al.*, 2012 ▸). This may be due to challenges during the EXAFS analysis of Ln oxides. A few studies address this issue, but they are limited to a small number of compounds and fail to create a comprehensive resource for the Ln oxides (Malet *et al.*, 1994 ▸; Fonda *et al.*, 1999 ▸). Thus, for many of the Ln oxides, the effects of multielectron excitations (MEEs), limited *k*-range and lattice geometry have not been examined in-depth.

For the lanthanides early in the series (La–Nd), highly visible erroneous peaks around a photoelectron wavenumber of 6 Å^−1^ in *k*-space are attributed to an MEE (2*p*, 4*d* → 5*d*^2^ transitions for the *L*_3_- and *L*_2_-edges and 2*s*, 4*d* → 6*p*, 5*d* for the *L*_1_-edge; Solera *et al.*, 1995 ▸; D’Angelo *et al.*, 2008 ▸). These MEEs have been shown to produce errors of around 0.02 Å in the interatomic distance (Solera *et al.*, 1995 ▸; Ohta *et al.*, 2009 ▸) and ∼10% in the CN (Chaboy *et al.*, 1994 ▸) during fitting. With limited literature describing accurate MEE removal for the lanthanides, it is often unclear whether the resulting spectrum has been ‘corrected’. This problem is further exacerbated by the limited *k*-range of the *L*-edges of lanthanides. The *L*-edges (which occur in the energy range 5–10 keV) of the lanthanides have limited range in the EXAFS region due to the presence of the next absorption edge. For instance, the edge energies for the *L*_3_- and *L*_2_-edges of lanthanum are 5483 and 5891 eV, respectively. This is only 408 eV or 10.3 Å^−1^ between the edges, which limits the *k*-range and leads to poor data resolution in the Fourier transform (FT) and limited information content in the data (Malet *et al.*, 1994 ▸; Calvin, 2013 ▸). Additional complications such as multiple absorbing sites can strain the information content of the data further due to the need for additional scattering paths to model each unique absorbing site (Ravel, 2014 ▸). Some analytical methods, such as the parameterization by assuming isotropic cubic expansion of a crystal lattice, are relatively simple to apply and can reduce the number of fitting parameters (Ravel, 2016*a* ▸). Geometric parameterization of non-cubic lattices is seldom carried out because of its complexity but can be useful for structural studies with severe information constraints (Ravel, 2009 ▸).

In this study, we collected and analyzed the EXAFS of the entire series of Ln oxides (excluding promethium due to its radioactivity) and confirmed the phase purity of each Ln by X-ray diffraction (XRD) prior to analysis. We achieved high-quality fits for all the Ln oxides using several techniques including fuzzy degeneracy, aggregation of unique absorbing sites, cubic and non-cubic crystal lattice expansion, and removal of MEEs. We explore a comprehensive subset of the Ln oxides (*e.g.* absorption edge, modeling technique and crystal lattice group) and summarize the results for the entire Ln series. Further details on the EXAFS fits of other Ln oxides along with sample preparation and phase confirmation are provided in Sections S2 and S3 of the supporting information.

## 2. Experimental

### 2.1. Materials and sample preparation

Lanthanide oxide compounds in the form Ln_2_O_3_ – lanthanide sesquioxides – were chosen for all elements except Ce. The tetravalent form of cerium, CeO_2_, was used owing to its application in catalysis and its prevalence in the literature. Pr_2_O_3_ and Tb_2_O_3_ were purchased from Sigma Aldrich at above 99.9% trace metal purity. All other Ln oxides were provided by the Mineralogical Society of America with a trace metal purity greater than 99.9%. A total of 100 mg of each oxide was calcined in air in a ceramic crucible following the steps outlined in Table S2 of the supporting information. For EXAFS measurements the samples were diluted using cellulose according to the *CatMass* Python package (Perez-Aguilar *et al.*, 2023 ▸) so that a 7 mm-diameter pellet had a total mass between 10 and 25 mg with an estimated edge step at the Ln *L*_III_-edge of approximately 0.85 when measured in transmission geometry with the sample perpendicular to the X-ray beam. The powder mixture was then ground with a mortar and pestle. For all samples, the average crystallite was not nanosized. More crystallite size information can be found in Section S2. The specified mass of the oxide–cellulose mixture was then pelletized (7 mm diameter) and wrapped in Kapton tape prior to mounting at the beamline. A table of dilution factors and preparations is included in Table S3.

### 2.2. X-ray absorption spectroscopy data collection

The X-ray absorption spectra were collected at beamlines 9-3 and 10-2 at the Stanford Synchrotron Radiation Lightsource within the SLAC National Accelerator Laboratory. Beamline 9-3 is a wiggler-based side station equipped with an Si(220), φ = 90°, double-bounce liquid-nitro­gen-cooled monochromator and Rh-coated collimating and focusing mirrors located before and after the monochromator, respectively. The beamline 9-3 mirrors were operated in a 10 keV cutoff mode with the monochromator detuned by 40% to minimize the effect of harmonics. The beam size was 1.0 mm (vertical) × 4.0 mm (horizontal). The flux of the X-rays is estimated to be 2 × 10^12^ photons s^−1^ in a spot size of 1 × 4 mm. Beamline 10-2 is a wiggler-based beamline with an Si(111), φ = 90^o^, double-bounce liquid-nitro­gen-cooled monochromator. The beamline 10-2 monochromator was detuned by 30% to minimize the effect of harmonics and the beam size was 0.25 mm (vertical) × 5.0 mm (horizontal). The estimated flux of the X-rays was 5 × 10^11^ photons s^−1^ given the beam size. EXAFS spectra were collected in step-scanning mode with the samples in transmission geometry. Nitro­gen-gas-filled ionization chambers were used to measure the relative intensity of the X-ray beam. Reference spectra of either a manganese, nickel, cobalt, zinc, copper or iron foil were collected simultaneously as an internal energy standard in transmission geometry by an off-axis photodiode or nitro­gen-filled ionization chamber. The reference foils were additionally scanned without the sample to be used for alignment in the data processing. For each sample, four–nine spectra were collected to improve the signal-to-noise ratio.

The *Demeter* software package (Ravel & Newville, 2005 ▸) was used for all XAS analysis and fitting. Each metal foil was calibrated to its tabulated edge energy (Henke *et al.*, 1993 ▸) using the first-derivative maxima to define the edge position. The reference spectrum on an Ln scan was then aligned to the foil spectrum to calibrate the edge energy of the Ln. The EXAFS was isolated by subtracting the background with an Rbkg of 1.0. The pre-edge and post-edge were set, and a cubic spline was used to normalize the data. Details on the EXAFS modeling for each Ln oxide can be found in Section S2. The crystal structures used in the generation of theoretical scattering paths and phase confirmation of our samples (by XRD) can be found in Section S3.

### 2.3. X-ray diffraction

Powder XRD was used to confirm the phase purity of the Ln oxides by comparing peak positions with those in the experimental data from the International Centre for Diffraction Data PDF4 database (Gates-Rector & Blanton, 2019 ▸) using the *Jade* software (version 8.7; Materials Data Inc.). Samples were front-loaded into a silicon zero-background holder and diffraction data were collected from 15 to 70° 2θ using a Malvern Panalytical Empyrean instrument fitted with a copper (*K*α_1_ = 1.540598, *K*α_2_ = 1.544426 Å) long-fine-focus X-ray tube operated at 45 kV and 40 mA. The incident beam path included iCore optics fitted with a BBHD optic with 0.03 radian Soller slits, a 14 mm primary and a 14 mm secondary mask, and a fixed 1/4° divergence slit. The diffracted beam path incorporated dCore optics with a 1/4° fixed anti-scatter slit and 0.04 radian Soller slits. A PIXcel3D detector was used in scanning line (1D) mode with an active length of 3.347° 2θ. Data were collected with a nominal step size of 0.0263° 2θ for 96.39 s for a total scan time of 15 min. Peak half-width at half-maximum deviation lower and upper levels were set at 4.02 and 11.27 keV, respectively. The data collected are given in Section S3.

## 3. Results and discussion

The *L*-edge EXAFS of each Ln oxide (excluding promethium due to its radioactivity) was recorded. Table 1 ▸ lists each compound, its crystal lattice information, the techniques used for EXAFS modeling, the edge that was used and the *R*-factor for each of the EXAFS fits. On average, slightly higher *R*-factors were observed for the lanthanides earlier in the Ln series (light lanthanides) and for lanthanides where the *L*_2_-edge was used for the EXAFS modeling. These higher *R*-factors are probably due to the more restricted *k*-range for these lanthanides (Section S1) and the greater influence of MEEs for the light lanthanides. In the following sections, several Ln oxides are used to demonstrate the EXAFS modeling approach for groups of compounds with similar crystal structures and the associated challenges. Table 1 ▸ shows the three distinct groups of compounds considered in this work: cubic Ln oxides, non-cubic Ln oxides and light Ln oxides.

The group of cubic Ln oxides, which have two unique crystallographic sites, consists of Pr and Sm through Lu (excluding CeO_2_ due to its space group). We examine the effect of aggregating these unique sites and applying cubic expansion to the EXAFS fit. The second group is non-cubic Ln oxides which includes La and Nd. A non-cubic expansion is completed for these compounds. The final group is the light Ln oxides which have observable MEEs (La, Ce and Pr). Other studies have reported minimal effect of MEEs on Nd and heavier lanthanides (Ohta *et al.*, 2008 ▸; Karabulut *et al.*, 2002 ▸). However, a weak EXAFS signal can increase the prevalence of the MEE peak, making the effect more pronounced for disordered materials (Fonda *et al.*, 1999 ▸). For these light Ln oxides, the effect of MEE removal on the EXAFS fits is observed.

Four different Ln oxides that span all three absorption edges and analysis groups (bold in Table 1 ▸) are discussed in the main text to provide a comprehensive summary of the EXAFS modeling. The EXAFS fits and modeling choices are provided in Section S2 for all Ln oxides not discussed in the main text. To summarize all these results, the first shell Ln—O distances are displayed in Fig. 6 and validated against the XRD results and the trend expected due to the lanthanide contraction (Bart, 2023 ▸; Jordan, 2023 ▸).

### 3.1. EXAFS of cubic lanthanide oxides

#### 3.1.1. Accounting for multiple unique absorbing sites

The heavier Ln oxides (with higher atomic numbers) exhibit a cubic, bixbyite crystal structure. The bixbyite crystal is comparable to a fluorite crystal (*e.g.* that of CeO_2_) except it is more complex due to oxygen deficiencies that lead to the creation of two unique cation sites. These two sites are labeled the 24*d* and 8*b* sites using Wyckoff notation. The 24*d* site is more prevalent than the 8*b* site (75% of the sites are 24*d*) and has distorted coordination with three pairs of oxygen atoms having different distances to the cation site. The 8*b* site has diagonally opposed oxygen deficiencies that retain six oxygen atoms at identical distances to the cation (Niu *et al.*, 2013 ▸; Stanek *et al.*, 2007 ▸). In the context of EXAFS analysis, considering the two Ln absorbing sites in addition to the multitude of unique oxygen distances requires at least 12 scattering paths to capture the first two shells (four Ln—O paths and two Ln—Ln paths for each absorbing site). Fitting each path can require a unique set of fitting parameters, which can strain the information content of the data (varying more parameters than there are independent parameters in the data). This makes fitting the *L*-edge EXAFS of the lanthanides especially challenging due to the limited *k*-range.

An approach to simplify the number of unique scattering paths is to use ‘fuzzy degeneracy’ in which nearly degenerate paths (*i.e.* paths from the same atom types that differ in half-path length by less than a set distance) are considered fully degenerate in the fitting process. This ‘fuzziness margin’ should be less than the spatial resolution of the data to obtain accurate results [spatial resolution = π/(2Δ*k*), where Δ*k* is the *k*-range used in the fit]. Using this fuzziness margin, similar paths for each absorbing site can be aggregated into one path to simplify the fitting process. However, fuzzy degeneracy can affect the signal due to the increase in the static disorder of the combined path [as much as 23 × 10^−3^ Å^2^ to the mean square relative displacement (MSRD), also known as the Debye–Waller factor (Smerigan *et al.*, 2023 ▸)], especially with large *k*-ranges (Ravel, 2014 ▸). However, the restricted *k*-range of the Ln *L*-edges makes this technique ideal and MSRDs reported here are comparable to those of similar studies (Schlegel *et al.*, 2004 ▸; Niu *et al.*, 2013 ▸; Fonda *et al.*, 1999 ▸; Malet *et al.*, 1994 ▸).

**Figure 1:**
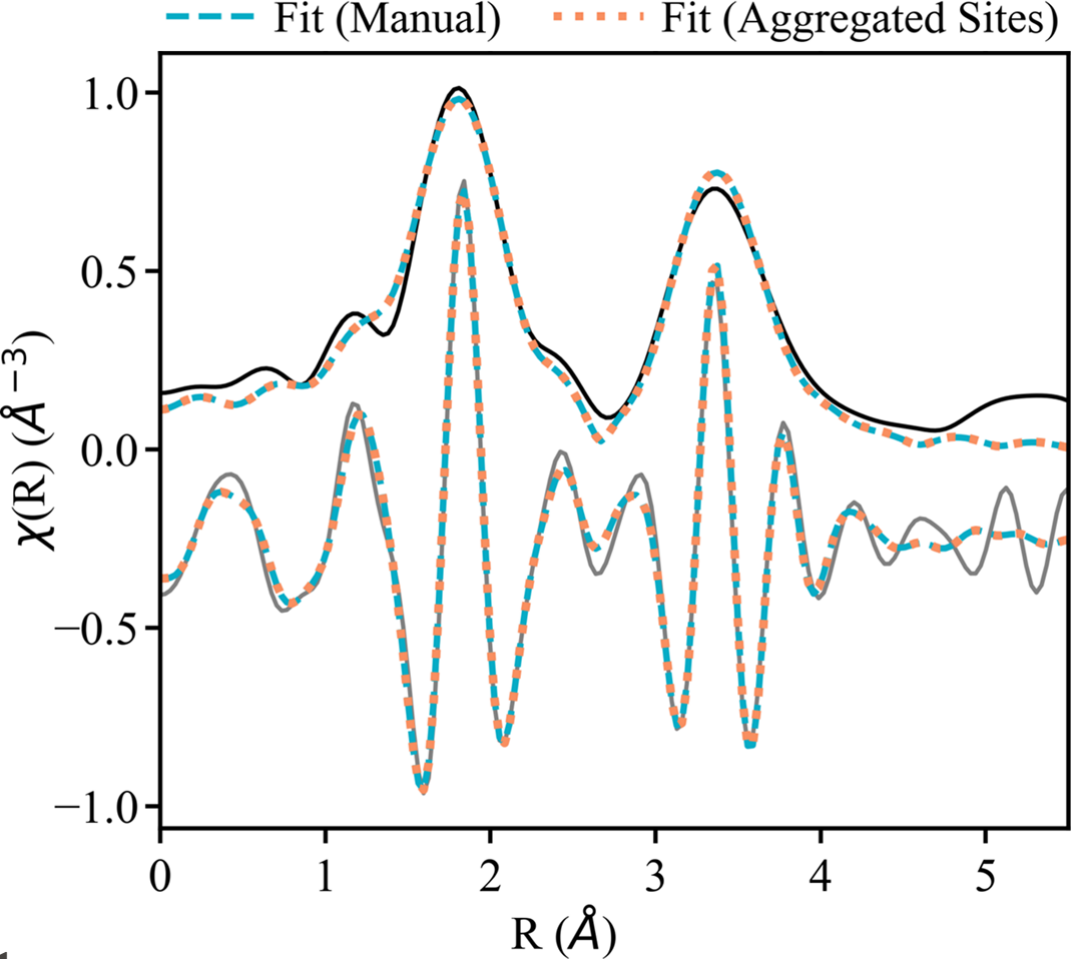
The magnitude and real part of the *k*^2^-weighted FT of the EXAFS for the Tm_2_O_3_*L*_3_-edge (solid lines). Fits with the *k*-range 3.7–10.5 Å^−1^ and *R*-range 1.3–4.2 Å are represented by the dashed (manual fit) and dotted (aggregated sites fit) lines.

We compared two methods for analyzing the two unique absorbing sites. One fit, the ‘manual’ fit, modeled each unique absorbing site individually by multiplying the amplitude reduction factor (S_o_^2^) of each path by the fraction of absorber in that site (*e.g.* 0.25 for the 8*b* cation site). The other fit, the ‘aggregated sites’ fit, used fuzzy degeneracy to simplify the EXAFS analysis by condensing contributions from similar paths of each absorbing site into one path. Fig. 1 ▸ shows that the two methods are closely similar qualitatively for a representative compound, Tm_2_O_3_.

For both fits, the amplitude reduction factor was set to 1.0, as was done in similar studies (Ohta *et al.*, 2009 ▸; Schlegel *et al.*, 2004 ▸). We did not observe large differences in the fit on changing the value of *S*_o_^2^. The addition of multiple-scattering paths within the fitting range (*k*-range 3.7–10 Å^−1^ and *R*-range 1.3–4.2 Å) and oxygen scatterers >4 Å did not considerably change the results and these were omitted. We attempted a fit with a Fourier transform over a larger range in *k*-space (2.8–10 Å^−1^), but this significantly decreased the quality of the fit. This is probably due to strong edge effects on the early EXAFS. The first shell Ln—O and Ln—Ln scattering paths were allowed to have different mean squared radial dis­place­ments (MSRDs, σ^2^) and adjustment in half-path length (Δ*R*), which were varied in the fit. CNs were fixed to their bulk crystallographic values. The change in edge energy (Δ*E*_o_) was varied to align the theoretical paths to the experimental results. Both fits used the same parameterization scheme to ensure any difference in fit could be attributed to aggregation.

The results of the ‘manual’ and ‘aggregated sites’ fits are similar (Table 2 ▸). The largest difference is in the MSRD of the Tm—Tm scattering path (0.5 × 10^−3^ Å^2^), which is well within the error of the fit (∼2.5 × 10^−3^ Å^2^). The interatomic distances (*R*) of the fits are also similar with only a minor difference in Tm—O and Tm—Tm (0.01 ± 0.01 Å). Further, the statistics of the fits show no change in *R*-factor (0.015) and only a slight improvement in reduced χ^2^ for the aggregated fit (a decrease from 1756 to 1715). This suggests that aggregating the two unique absorbing sites for the cubic Ln oxides is an applicable method for simplifying the analysis and minimizing the information content required during EXAFS modeling. Therefore, aggregation was used to fit all cubic Ln oxides of the same crystallographic space group (*Ia*3). The EXAFS spectra and EXAFS modeling results for these other cubic Ln oxides can be found in Section S2.

#### 3.1.2. Cubic expansion

Using a physical parameterization (*i.e.* constraining fit parameters through knowledge of the sample) in an EXAFS model can lead to more physically reasonable fits. One common approach for crystalline samples is to use the cubic lattice expansion approximation, which uses lattice constants to model expansion or contraction of a crystal lattice. If an isotropic expansion is assumed, for which all unit-cell lengths are equal (*a* = *b* = *c*), Δ*R* of any path can be described by equation (1):
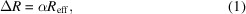
where the volume expansion coefficient α is a varied parameter in the fit representing the extent of expansion or contraction, and the effective path length *R*_eff_ is fixed by the input crystal structure. A derivation and example is available in the *Artemis* documentation and the book by Ravel (2016*a* ▸,*b* ▸).

**Figure 2:**
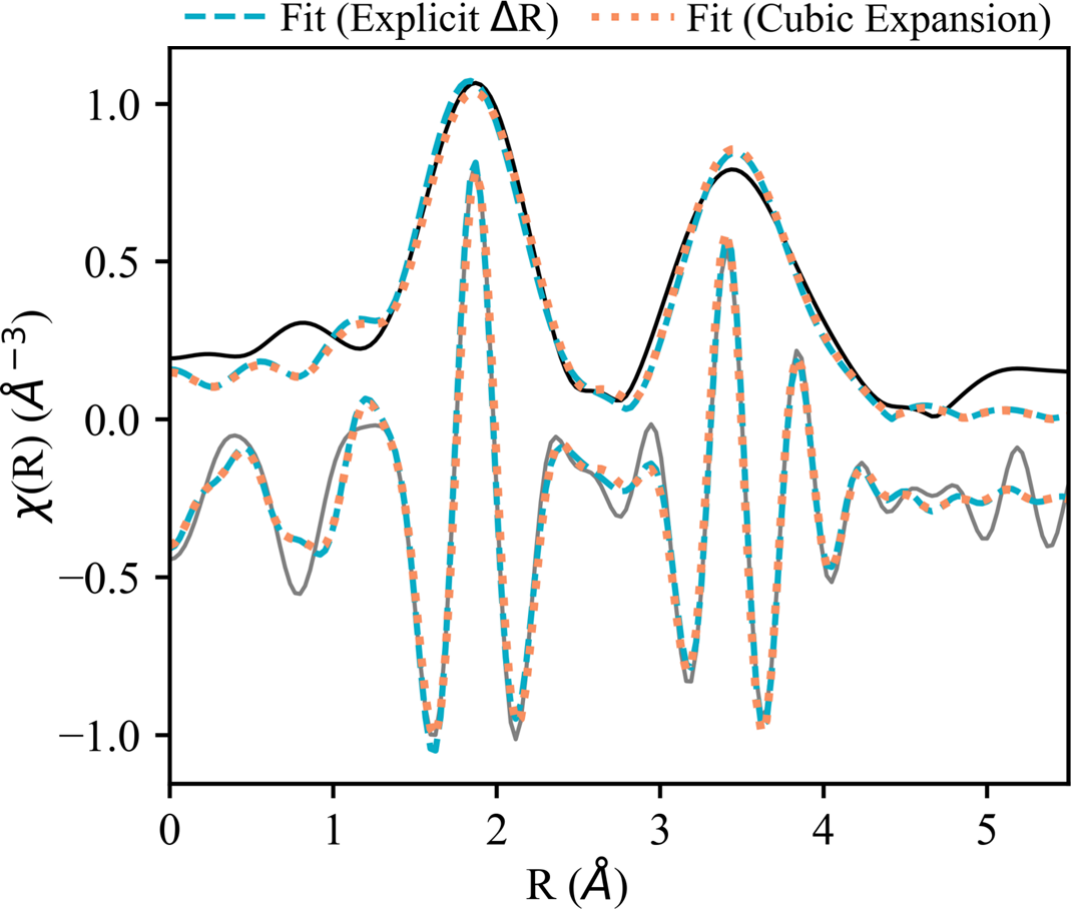
The magnitude and real part of the *k*^2^-weighted FT of the EXAFS for the Dy_2_O_3_*L*_2_-edge (solid lines). Fits with the *k*-range 3.65–9.76 Å^−1^ and *R*-range 1.3–4.2 Å are represented by dashed (explicit Δ*R*) and dotted (cubic expansion) lines.

We compared a fit that used the ‘cubic expansion’ fit with another that did not use cubic expansion: the ‘explicit Δ*R*’ fit. The *L*_2_-edge of Dy_2_O_3_ is shown in Fig. 2 ▸ and is representative of the fits for other cubic Ln oxides. Fig. 2 ▸ shows that the two fits are qualitatively similar. Both fits were completed using aggregation of inequivalent absorbing sites, as described in Section 3.1.1. *S*_o_^2^ was fixed at 1.0 and the CN was fixed to the bulk crystallographic value for all paths. Δ*R* and σ^2^ were allowed to vary for each path in the fits. In the ‘cubic expansion fit’, Δ*R* for each path was solved according to equation (1). The ‘explicit Δ*R* fit’ varied Δ*R* for each path. Δ*E*_o_ was varied and the value was shared for all paths. The results of these fits are compared in Table 3 ▸.

We found that both fits are reasonable with the explicit Δ*R* fit having better statistics than the cubic expansion fit (respective *R*-factors of 0.012 and 0.020). These fits also show slightly different distances and disorder. The largest difference in distance is for the Dy—O path at around 2.30 Å, which has a difference of ∼0.02 ± 0.01 Å between the two fits. This difference in distance is also accompanied by a slight change in MSRD (5.7 ± 1.9 × 10^−3^ and 6.0 ± 1.0 × 10^−3^ Å^2^ for the explicit Δ*R* fit and cubic expansion fit, respectively). The ‘cubic expansion’ fit has a larger *R*-factor than the ‘explicit Δ*R*’ fit (0.020 to 0.012) and a higher reduced χ^2^ (2369 to 1846). This could be explained by a poor assumption of isotropic expansion of the crystal lattice. It could also be due to fewer independent points being used in the cubic expansion fit (5 compared with 7 in the ‘explicit Δ*R*’ fit). The decrease in fit quality after applying the cubic expansion was also observed for other Ln oxides (*R*-factors ranging from ∼0.02 to 0.07). Since the ‘explicit Δ*R*’ fit performs statistically better in all cases, we used it to fit the remaining cubic Ln oxides (Section S2). However, cubic expansion can be useful to reduce the number of fitted parameters when information content is more severely constrained.

### 3.2. EXAFS of non-cubic lanthanide oxides

Geometric parameterization of a non-cubic crystal lattice is more complicated thanthat of its cubic counterpart. Non-cubic Ln oxides, like Nd_2_O_3_, form a trigonal lattice with the lattice constants *a* = *b* ≠ *c* and angles α = β = 90° and γ = 120°. This forms an oblique coordinate system where one axis is shifted 30°. The geometric parameterization mainly consists of four varied parameters and as many defined parameters as there are scattering paths being fitted. The two unique values of lattice constants are both varied parameters in the fit. The other two varied parameters are the displacements of the neodymium and oxygen atoms from the *c* axis of the lattice. Displacements from the other axes are unnecessary since the lattice constants are equal and they will expand evenly. The position of each atom in the lattice can be defined by a geometric factor multiplied by these lattice constants. The geometric factor is determined by finding the position of the absorbing atom and scattering atom in the unit cell. Using each atom’s position, the distance formula and varied parameters can be used to determine the distance between every scattering and absorbing atom (after adding a correction for the oblique coordinate system). This ties the position of each atom in the lattice to the four varied parameters which are allowed to optimize during the fitting process. It also provides a way to model non-isotropic expansion or contraction of any crystal lattice. Equation (2) shows a generalized form of the equation used for determining the distance of any path in the Nd_2_O_3_ lattice:
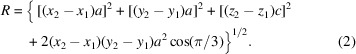
In equation (2), *x*, *y* and *z* are positions in the unit cell corresponding to the direction of the *a*, *b* and *c* lattice parameters, respectively. Since *a* = *b* in this lattice, lattice parameter *b* was replaced with *a*. Variables with a subscript of 1 are positions of the absorbing atom and subscript of 2 are the scattering atoms. The (*z*_2_ − *z*_1_) term should be written in terms of the displacements of the central neodymium and oxygen atoms from the *c* axis since these are varied parameters. The last term in the curly brackets is the correction for the oblique coordinate system.

For the ‘non-cubic expansion’ fit, Δ*R* is defined as the scattering distance calculated in equation (2) minus the *R*_eff_ of the path. The ‘explicit Δ*R*’ fit varied the Δ*R* of paths explicitly. For both fits, an *S*_o_^2^ of 1 was fixed and the CN was set to the bulk crystallographic value for each path. All paths shared the same Δ*E*_o_ since they came from the same crystal structure. Paths of similar lengths were given their own σ^2^ in both fits. The exact formulae to determine path lengths are available in Tables S10 and S11, including all varied, set and defined parameters for the non-cubic expansion fit.

**Figure 3:**
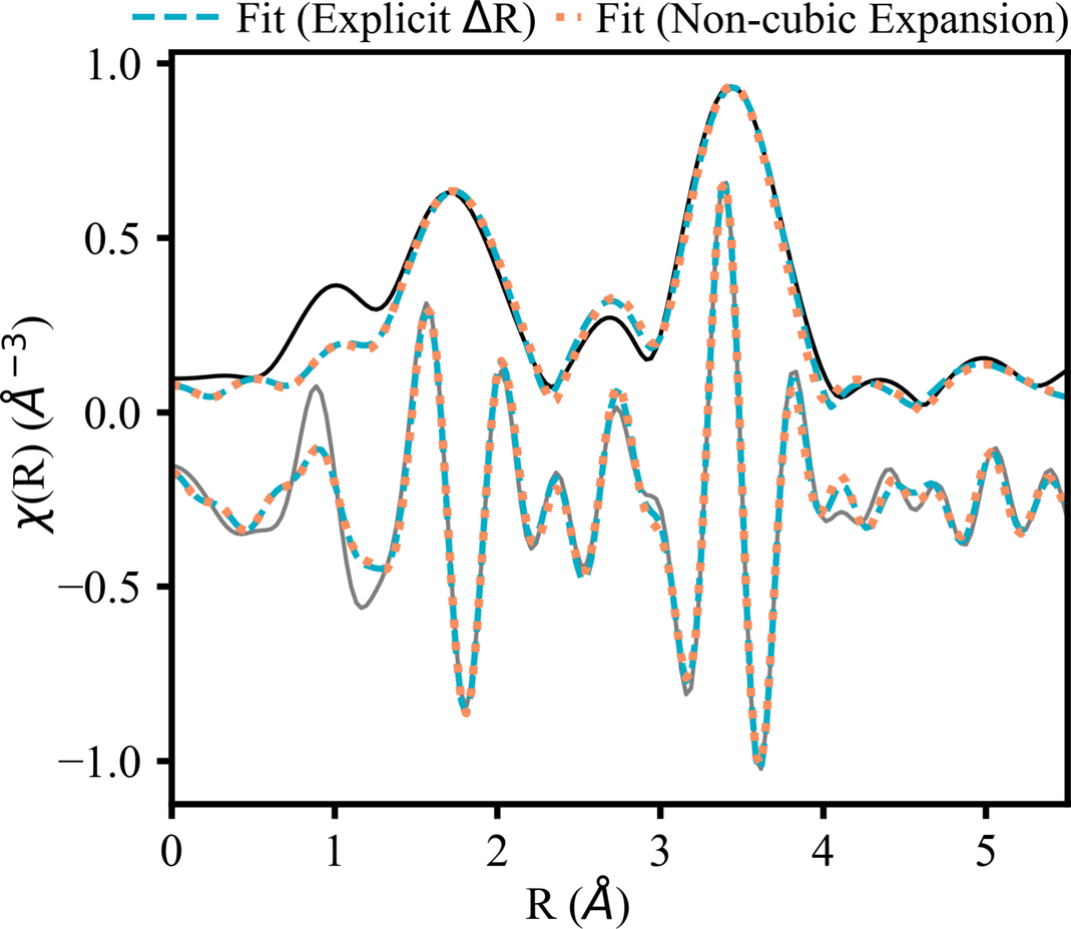
The magnitude and real part of the *k*^2^-weighted FT of the EXAFS for the Nd_2_O_3_*L*_1_-edge (solid lines). Fits with the *k*-range 3.3–10.1 Å^−1^ and *R*-range 1.3–5.4 Å are represented by dashed (explicit Δ*R*) and dotted (non-cubic expansion) lines.

The non-cubic expansion fit is almost indiscernible from the non-geometric fit shown in Fig. 3 ▸. Both fits fail to perfectly capture the amplitude of the peak at 2.75 Å. The path that contributes the most in this region is an Nd—O path at 2.66 Å (Table 4 ▸), which has a relatively high MSRD (0.0128 Å^2^). The value of the MSRD of this Nd—O path is ∼5× larger than the next shortest Nd—O path and 2× as large as longer Nd—O paths. However, it is comparable to that found in a similar study (Fabian *et al.*, 2021 ▸). Other than the high MSRD for this Nd—O scattering path, both fits have reasonable results and statistics (*R*-factors less than 0.02). In addition, both fits have similar MSRD values and interatomic distances. An additional benefit of using the non-cubic expansion is that the parameterization physically represents the system. For instance, we can see whether the trigonal crystal lattice of Nd_2_O_3_ expands isotropically by looking at the fitted value of the lattice parameters (*a* = 3.84 and *c* = 6.08) compared with the original values (*a*_o_ = 3.83 and *c*_o_ = 6.00). We observed a 0.3% increase in the *a* direction and a 1.3% increase in the *c* direction, suggesting that expansion is not isotropic in this lattice. Overall, the non-cubic expansion fit is comparable to the non-geometric fit, while using the same number of parameters to provide more information about the system.

### 3.3. EXAFS of light lanthanide oxides

**Figure 4:**
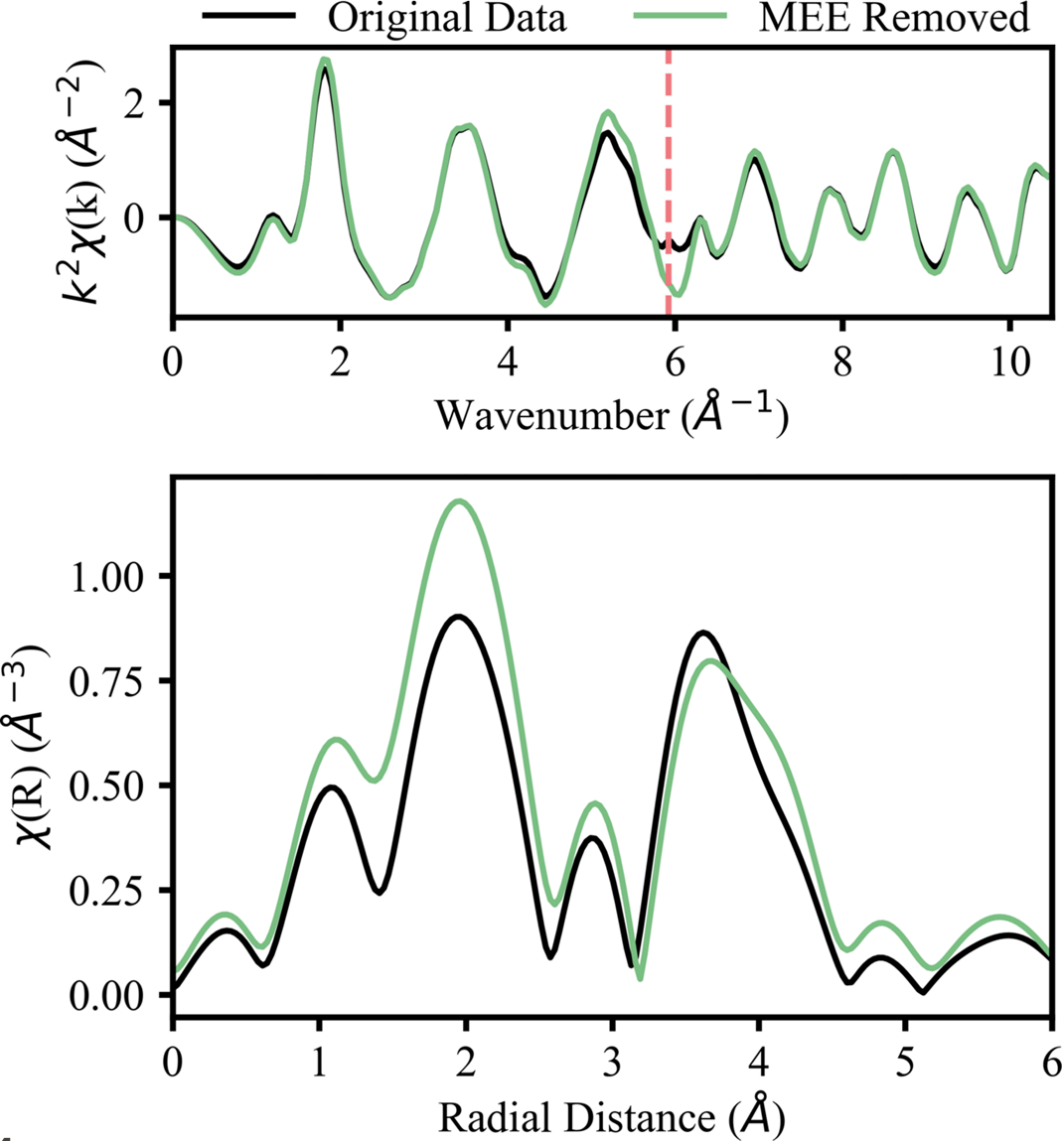
Comparison of the *k*-space (top) and *R*-space (bottom) before and after MEE removal of the CeO_2_*L*_3_-edge. The location of the MEE in *k*-space is indicated by a vertical, red dashed line.

As stated previously, the EXAFS of the *L*-edges of light lanthanides is complicated by the 2*p*, 4*d* → 5*d*^2^ transitions for the *L*_3_- and *L*_2_-edges and 2*s*, 4*d* → 6*p*, 5*d* for the *L*_1_-edge (Solera *et al.*, 1995 ▸; D’Angelo *et al.*, 2008 ▸). This additional electron that is excited, known as an MEE, results in an erroneous feature in the EXAFS spectrum as shown by the vertical line in Fig. 4 ▸. These MEEs have been shown to produce errors of around 0.02 Å in interatomic distances (Solera *et al.*, 1995 ▸; Ohta *et al.*, 2009 ▸) and 10% in CNs (Chaboy *et al.*, 1994 ▸) during fitting (Ohta *et al.*, 2008 ▸). Some studies have deglitched the MEE out of the spectra (Allen *et al.*, 2000 ▸; Mayanovic *et al.*, 2009 ▸), while others use functions to reduce the prevalence of the MEE features (D’Angelo *et al.*, 2008 ▸; Ohta *et al.*, 2009 ▸). Here, we used the reflection algorithm in the MEE removal tool in *Athena* (Ravel & Newville, 2005 ▸) to remove the MEE from the EXAFS of La_2_O_3_, CeO_2_ and Pr_2_O_3_. We show the MEE removal and resulting EXAFS fits for the light Ln oxides in Section S2. The EXAFS of the lanthanides heavier than praseodymium did not have distinguishable MEEs and MEE removal was not considered during EXAFS modeling.

**Figure 5:**
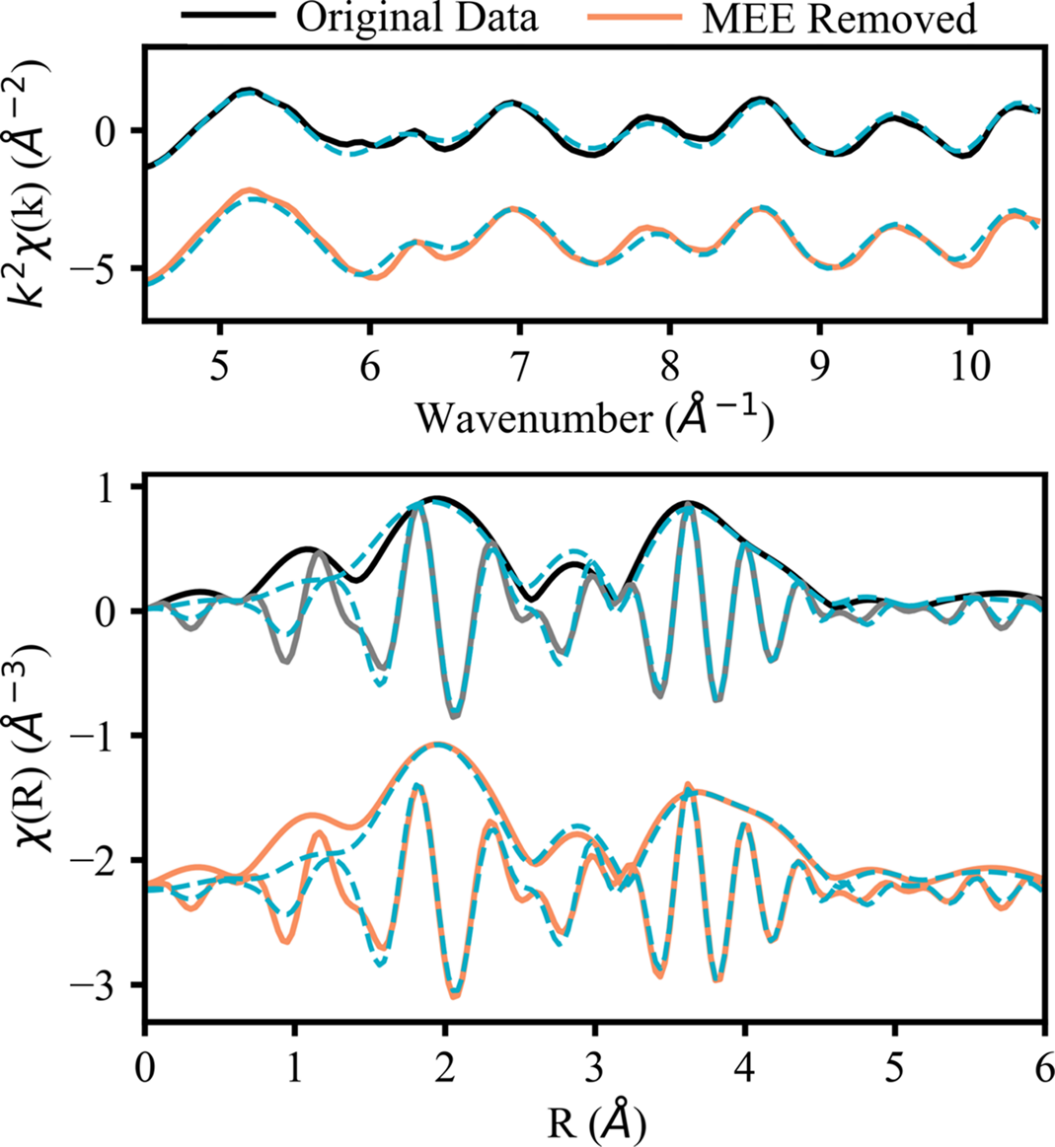
EXAFS of the CeO_2_*L*_3_-edge with the *k*-space (top) and the magnitude and real part of the FT (bottom). Experimental data are shown as solid lines. The orange spectra, data after MEE removal, are shifted down 2.25 Å for comparison with the ‘original data’ in black. Fits with the *k*-range 4.5–10.1 Å^−1^ and *R*-range 1.5–6.1 Å are shown by blue dashed lines.

The compound CeO_2_ was chosen as a representative light Ln to investigate the effect of MEEs on EXAFS results. As part of the MEE-removal process, we fit the *R*-space of the CeO_2_*L*_3_-edge for the spectrum still containing the MEE (the ‘original data’ fit) and without the MEE (the ‘MEE removed’ fit). Using physically reasonable limits from the literature (Solera *et al.*, 1995 ▸; Ohta *et al.*, 2008 ▸), MEE-removal parameters can be adjusted to achieve a better removal, both visually and quantitatively. Different attempts at MEE removal were completed in *Athena* and then fitted in *Artemis*. The *R*-factor was tracked for each fit until a minimum was reached. We found that MEE removal makes a small but noticeable improvement in a fit, as seen in Fig. 5 ▸ and Table 5 ▸. Therefore, a poor fit (*R*-factor > 0.05) prior to MEE removal indicates a fundamental problem (*e.g.* an incorrect crystal structure or fitting procedure). MEE removal should only be used as a way to refine an already suitable fit since the influence of an MEE is minimal (Solera *et al.*, 1995 ▸; Ohta *et al.*, 2008 ▸). The MEE-removal process is highly user dependent, and the resulting spectrum will remain influenced by the MEE to a small degree. Future work should aim to standardize the process of MEE removal for the Ln elements for simpler and more accurate EXAFS modeling. The FT in the bottom of Fig. 5 ▸ shows how MEE removal qualitatively improves the fit in several places: in the rising edge of the peak at 2 Å, the amplitude of the peak at 3 Å and the rising edge of the broad peak at 4 Å.

To complete the EXAFS fits, only single-scattering paths were significant. Inclusion of multiple scattering paths had minimal effect on the goodness of fit and were ignored for simplicity (considering the *k*-range 4.5–10.1 Å^−1^ and *R*-range 1.5–6.1 Å). An *S*_o_^2^ of 1.0 was used for the fit and *N* was set to the crystallographic values. Δ*E*_o_ was varied and shared by all scattering paths. Each scattering path was allowed to vary Δ*R* explicitly. The shortest three paths were each given an MSRD. To constrain the MSRDs, we gave the Ce—Ce path at around 5.4 Å an MSRD 1.5× larger than the MSRD of the shorter Ce—Ce path (∼3.86 Å). Similarly, we gave the Ce—O path at around 5.95 Å an MSRD 1.5× larger than the MSRD of the shorter Ce—O path (∼4.42 Å). Constraining these MSRDs by element and length resulted in better fits with lower *R*-factors than when we grouped MSRDs by just path lengths. In the fit, we only considered EXAFS data with wavenumbers greater than 4.5 Å^−1^ to minimize the impact of the final-state mixed valence of CeO_2_ on the EXAFS analysis. Another study fitted two sets of theoretical phases and amplitudes, one shifted in energy, to account for the multivalency (Fonda *et al.*, 1999 ▸), but we did not see a significant difference in the fit when we attempted to replicate this method. Since we achieved a satisfactory fit without this method, we only considered the tetravalent EXAFS signal in this paper.

The results of the ‘original data’ fit and the ‘MEE removed’ fit are displayed in Table 5 ▸. The ‘MEE removed’ fit has a smaller *R*-factor (0.019 compared with 0.027 for the ‘original data’ fit), supporting the qualitative result of a better fit after MEE removal from Fig. 5 ▸. The fit parameters are reasonable (Δ*R* less than 0.1 Å and σ^2^ less than 0.02 Å^2^). Further, the path lengths are comparable to those observed in other EXAFS studies (Fonda *et al.*, 1999 ▸; Romanchuk *et al.*, 2022 ▸; Shahin *et al.*, 2005 ▸). Comparing the fits, the ‘original data’ fit shows that Ce—O paths have greater disorder, on average, than Ce—Ce paths (12.7 × 10^−3^ to 8.0 × 10^−3^ Å^2^, respectively). After removal of the MEE in the ‘MEE removed’ fit, the MSRDs of Ce—O paths decrease by about 33%, reducing the disparity in disorder between the Ce—O and Ce—Ce paths (9.1 × 10^−3^ and 8.0 × 10^−3^ Å^2^, respectively). This is consistent with the increase in amplitude observed in the first peak of the FT (primarily oxygen scattering) shown in Fig. 4 ▸. The differences in path length between the fits are around 0.03 ± 0.01 Å, which agrees with the conclusions of relevant literature of Ln MEE removal (Solera *et al.*, 1995 ▸; Ohta *et al.*, 2008 ▸). The EXAFS analysis before and after MEE removal for lanthanum oxide and praseodymium oxide show similar results and are included in Section S2.

### 3.4. Summary of EXAFS fits

**Figure 6:**
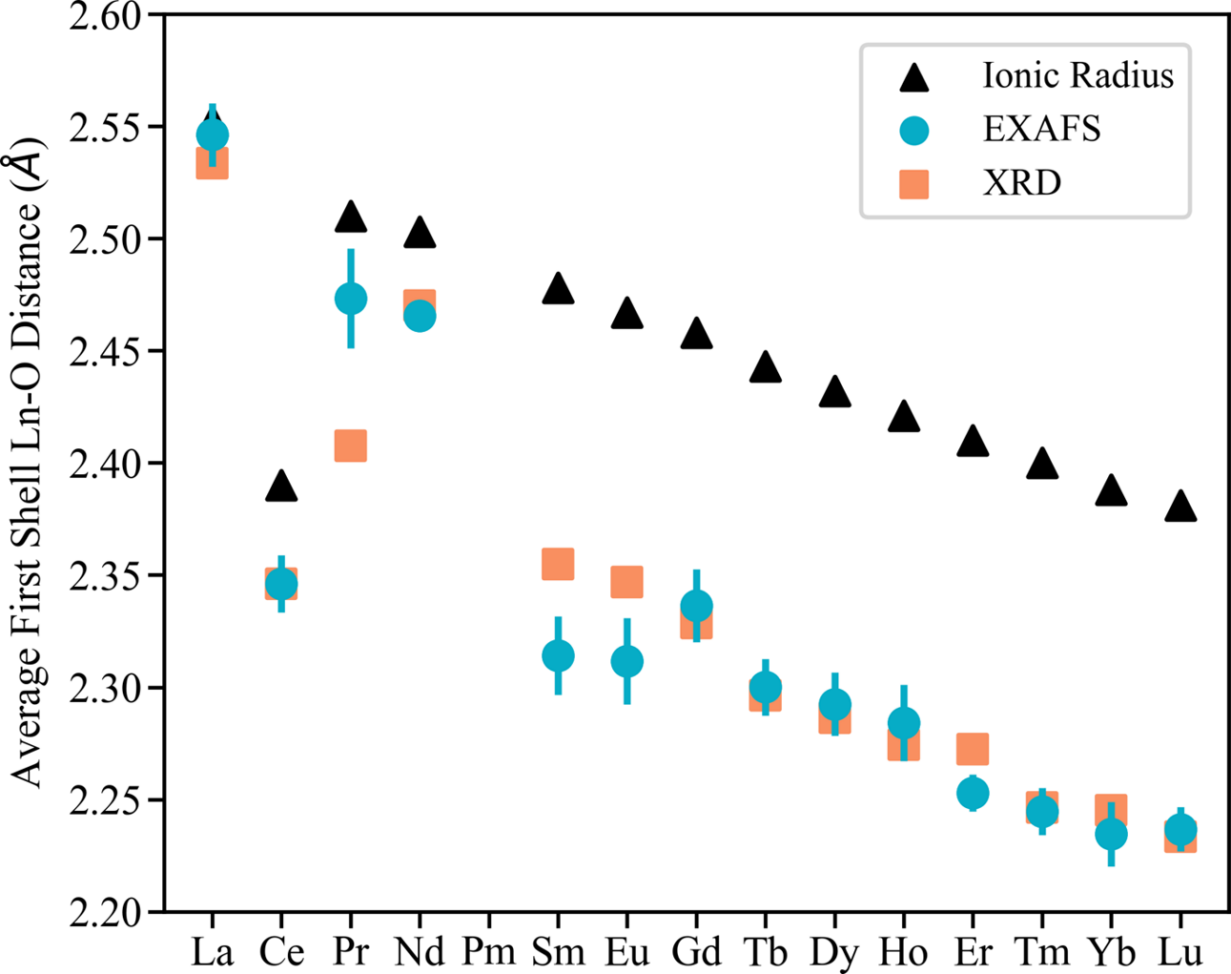
Average first shell Ln—O distances as determined by ionic radius, XRD and EXAFS analysis (this work). Distances generally follow the expected trend for Ln oxides and agree with XRD values.

A summary of the first shell Ln—O bond lengths is shown in Fig. 6 ▸ along with those from XRD and estimated distances using the ionic radius [ionic radius of the Ln plus the ionic radius of oxygen (Seaborg, 1993 ▸)]. As predicted by the lanthanide contraction (Bart, 2023 ▸; Jordan, 2023 ▸), the Ln—O bond lengths decrease across the Ln series. The EXAFS modeling results follow this trend well (other than CeO_2_ which is tetravalent as opposed to trivalent). Compared with the estimated Ln—O distances by ionic radii, all crystals have shorter distances with the hexagonal crystals (La and Nd) having longer Ln—O bonds than cubic bixbyite structures.

In addition, the EXAFS Ln—O distances generally agree with the XRD results, though Pr_2_O_3_, Sm_2_O_3_ and Eu_2_O_3_ stray the furthest from XRD values. For Pr_2_O_3_, we identified a minor phase of hexagonal Pr_2_O_3_ (shown in Section S3) that is likely making the Pr—O distance (2.47 ± 0.02 Å) longer than the pure cubic lattice. Other EXAFS studies have also reported slightly longer Pr—O distances (2.45 ± 0.02 Å) than the XRD structure used here (2.41 Å). As for Sm_2_O_3_ and Eu_2_O_3_, the distances determined by EXAFS are within two standard deviations of the XRD values. Since we used the *L*_2_-edge for these two compounds, we attribute the very small *k*-ranges (∼3.6–9.3 Å^−1^) of these fits to the more uncertain result. This finding emphasizes the challenge of EXAFS fitting for the Ln *L*_2_-edge, which has a smaller usable *k*-range than the *L*_3_-edge (Section S1). Further, the *L*_2_-edges may have residual signal from the *L*_3_-edge introducing additional error in the fit. It may be advantageous to perform simultaneous fitting of the *L*_3_- and *L*_2_-edges to reduce the uncertainty in fitting results and increase confidence in conclusions. Overall, we generated a simple and flexible EXAFS model for the *L*-edges of Ln oxides that agrees with XRD and follows the trend expected due to the lanthanide contraction.

Interestingly, the EXAFS spectra of these Ln oxide compounds do not display significant destructive interference as can be observed in similar systems (Martens *et al.*, 1985 ▸). This apparent lack of interference may be due to the significantly disordered state of the Ln oxide crystal structures studied here. Since there are many unique Ln scattering distances, the phases of these paths do not completely cancel each other resulting in a peak in the FT. Other more ordered Ln compounds may exhibit greater destructive interference with greatly diminished peaks.

## 4. Conclusions

In this study, we analyzed the *L*-edge EXAFS of the complete series of Ln oxides. All EXAFS spectra, best fit results (Section S2) and phase confirmation by XRD (Section S3) are provided. To achieve the best fit for the Ln oxides, we investigated the effects of aggregating inequivalent absorbing sites, geometric crystal expansion and MEE removal. In the limited *k*-range of the Ln *L*-edges, aggregation of unique absorbing sites had no effect on the EXAFS fit while reducing the complexity of the problem. For cubic lattices, fits that explicitly varied the deviation of path length from the XRD crystal structure for each path were statistically better than those that used a cubic crystal expansion. A non-cubic expansion of the trigonal Nd_2_O_3_ lattice produced a similar quality fit with evidence of non-isotropic expansion of the crystal. The oxides of light lanthanides (namely lanthanum, cerium and praseodymium) showed a visible MEE peak in their EXAFS around 5.9 Å^−1^. This MEE was removed, increasing the quality of the EXAFS fits. After MEE removal, the interatomic distances remained similar (within 0.03 Å) while the mean squared radial displacement of Ln—O scattering paths decreased by as much as 33%. Heavier lanthanides did not exhibit an easily identifiable MEE peak. The knowledge accumulated here can be used to develop greater structural insight into a wide variety of Ln-containing compounds (*i.e.* glasses, thin films and catalysts).

## 5. Related literature

The following references are cited in the supporting information for this article: Abdusalyamova *et al.* (2014 ▸); Abu-Zied & Asiri (2014 ▸); Abu-Zied *et al.* (2016 ▸); An *et al.* (2008 ▸); Azad & Maqsood (2014 ▸); Chen *et al.* (2010 ▸, 2016 ▸); Curtis & Tharp (1959 ▸); Djuričić & Pickering (1999 ▸); Duhan *et al.* (2008 ▸); Ekthammathat *et al.* (2015 ▸); El Desouky *et al.* (2020 ▸); Farahmandjou *et al.* (2016 ▸); Gao *et al.* (2003 ▸); Ghiasi & Malekzadeh (2015 ▸); Ghosh *et al.* (2021 ▸); Hosokawa *et al.* (2007 ▸); Hussein (2001 ▸); Hussein *et al.* (2000 ▸, 2003 ▸); Kirk & Wood (1995 ▸); Kumar *et al.* (2015 ▸); Lee *et al.* (2014 ▸); Leoni *et al.* (2004 ▸); Li *et al.* (2004 ▸, 2022 ▸); Mekhemer (2004 ▸); Mortazavi-Derazkola *et al.* (2015 ▸); Nachimuthu *et al.* (2000 ▸); Neumann & Walter (2006 ▸); Panitz (1999 ▸); Panitz *et al.* (1997 ▸); Rahimi-Nasrabadi *et al.* (2017*a* ▸,*b* ▸,*c* ▸,*d* ▸); Riva *et al.* (2016 ▸); Rosid *et al.* (2019 ▸); Rudraswamy & Dhananjaya (2012 ▸); Salavati-Niasari *et al.* (2010 ▸); Seo *et al.* (2013 ▸); Sidorowicz *et al.* (2016 ▸); Sirotinkin *et al.* (2022 ▸); Squire *et al.* (1994 ▸); Sunding *et al.* (2011 ▸); Tok *et al.* (2006 ▸); Tsuzuki *et al.* (1999 ▸); Watcharapasorn *et al.* (2008 ▸); Whba *et al.* (2021 ▸); Yang *et al.* (2008 ▸); Zawadzki & Kępiński (2004 ▸); Zhang *et al.* (2020 ▸); Zhao *et al.* (2013 ▸); Zinatloo-Ajabshir *et al.* (2017 ▸).

## Supplementary Material

Additional XRD and XAS data, including supporting figures and tables. DOI: 10.1107/S1600576724010240/xx5061sup1.pdf

## Figures and Tables

**Table 1:** List of Ln oxides analyzed in this paper with the *R*-factor of each EXAFS fit The Ln oxides were grouped based on the techniques used during EXAFS analysis, which are discussed in the remainder of the results. Bold indicates compounds that are examined within the main text. ‘Yes’ and ‘no’ indicate whether a compound is within a group for analysis.

			Group for analysis				
Compound	Crystal system	Space group	Cubic Ln oxide	Non-cubic Ln oxide	Light Ln oxide	Edge used for EXAFS fitting	*R*-factor
La_2_O_3_	Trigonal	*P*3*m*1	No	Yes	Yes	*L*_3_	0.018
**CeO_2_**	**Cubic**	***Fm* 3 *m***	**No**	**No**	**Yes**	***L*_3_**	**0.019**
Pr_2_O_3_	Cubic	*Ia* 3	Yes	No	Yes	*L*_3_	0.020
**Nd_2_O_3_**	**Trigonal**	**P3*m*1**	**No**	**Yes**	**No**	***L*_1_**	**0.012**
Sm_2_O_3_	Cubic	*Ia* 3	Yes	No	No	*L*_2_	0.020
Eu_2_O_3_	Cubic	*Ia* 3	Yes	No	No	*L*_2_	0.019
Gd_2_O_3_	Cubic	*Ia* 3	Yes	No	No	*L*_2_	0.014
Tb_2_O_3_	Cubic	*Ia* 3	Yes	No	No	*L*_3_	0.013
**Dy_2_O_3_**	**Cubic**	***Ia* 3**	**Yes**	**No**	**No**	***L*_2_**	**0.012**
Ho_2_O_3_	Cubic	***Ia* 3**	Yes	No	No	*L*_2_	0.015
Er_2_O_3_	Cubic	*Ia* 3	Yes	No	No	*L*_2_	0.009
**Tm_2_O_3_**	**Cubic**	***Ia* 3**	**Yes**	**No**	**No**	***L*_3_**	**0.015**
Yb_2_O_3_	Cubic	*Ia* 3	Yes	No	No	*L*_2_	0.019
Lu_2_O_3_	Cubic	*Ia* 3	Yes	No	No	*L*_2_	0.013

**Table 2:** EXAFS fits for the Tm_2_O_3_*L*_3_-edge with and without aggregation of inequivalent absorbing sites

		Manual fit	Aggregated sites fit
Tm—O	CN	6†	6†
	σ^2^ (10^−3^ Å^2^)	7.9 ± 1.7	7.9 ± 0.9
	Δ*R* (Å)	0.00 ± 0.01	0.00 ± 0.01
	*R* (Å)	2.25 ± 0.01‡	2.24 ± 0.01
Tm—Tm	CN	6†	6†
	σ^2^ (10^−3^ Å^2^)	5.0 ± 1.1	5.0 ± 1.1
	Δ*R* (Å)	0.01 ± 0.02	0.01 ± 0.01
	*R* (Å)	3.50 ± 0.02‡	3.51 ± 0.01
Tm—Tm	CN	6†	6†
	σ^2^ (10^−3^ Å^2^)	7.8 ± 3.1	7.3 ± 2.1
	Δ*R* (Å)	0.03 ± 0.04	0.03 ± 0.03
	*R* (Å)	3.96 ± 0.04‡	3.96 ± 0.03
All paths	Δ*E*_o_ (eV)	1.65 ± 1.59	1.68 ± 1.55
	*S*_o_^2^	1.0†	1.0†
Fit statistics	Independent points	12.3	12.3
	Number of parameters	7	7
	Reduced χ^2^	1756	1715
	*R*-factor	0.015	0.015
Fit range	*k*-range (Å^−1^)	3.7–10.5	3.7–10.5
	*R*-range (Å)	1.3–4.2	1.3–4.2
Fuzziness	Distance fuzz (Å)	0.10	0.10

† Parameters fixed during the fitting.

‡ Average of both absorbing sites.

**Table 3:** EXAFS fit results for the Dy_2_O_3_*L*_3_-edge using explicit Δ*R* values and cubic expansion

		Explicit Δ*R* fit	Cubic expansion fit
Dy—O	CN	6†	6†
	σ^2^ (10^−3^ Å^2^)	5.7 ± 1.9	6.0 ± 1.0
	Δ*R* (Å)	0.01 ± 0.00	0.02 ± 0.01
	*R* (Å)	2.29 ± 0.00	2.31 ± 0.01
Dy—Dy	CN	6†	6†
	σ^2^ (10^−3^ Å^2^)	3.7 ± 1.3	3.6 ± 1.3
	Δ*R* (Å)	0.03 ± 0.02	0.03 ± 0.01
	*R* (Å)	3.57 ± 0.02	3.57 ± 0.01
Dy—Dy	CN	6†	6†
	σ^2^ (10^−3^ Å^2^)	7.3 ± 2.4	6.9 ± 2.6
	Δ*R* (Å)	0.03 ± 0.03	0.04 ± 0.01
	*R* (Å)	4.04 ± 0.03	4.05 ± 0.01
Dy—O	CN	3†	3†
	σ^2^ (10^−3^ Å^2^)	7.3 ± 2.4	6.9 ± 2.6
	Δ*R* (Å)	0.03 ± 0.03	0.04 ± 0.01
	*R* (Å)	4.25 ± 0.03	4.27 ± 0.01
All paths	Δ*E*_o_ (eV)	2.27 ± 1.53	3.40 ± 1.53
	*S*_o_^2^	1.0†	1.0†
Fit statistics	Independent points	11.0	11.0
	Number of parameters	7	5
	Reduced χ^2^	1846	2369
	*R*-factor	0.012	0.020
Fit range	*k*-range (Å^−1^)	3.65–9.76	3.65–9.76
	*R*-range (Å)	1.3–4.2	1.3–4.2
Fuzziness	Distance fuzz (Å)	0.10	0.10

† Parameters fixed during the fitting.

**Table 4:** Comparing a standard fit with a fit using non-cubic expansion for the *L*_1_-edge of Nd_2_O_3_

		Explicit Δ*R*	Non-cubic expansion
Nd—O	CN	4†	4†
	σ^2^ (10^−3^ Å^2^)	3.6 ± 0.7	0.9 ± 1.5
	Δ*R* (Å)	0.00 ± 0.02	0.01 ± 0.02‡
	*R* (Å)	2.32 ± 0.02	2.34 ± 0.02‡
Nd—O	CN	3†	3†
	σ^2^ (10^−3^ Å^2^)	13.0 ± 3.4	13.0 ± 4.2
	Δ*R* (Å)	0.00 ± 0.02	0.02 ± 0.02
	*R* (Å)	2.66 ± 0.02	2.68 ± 0.02
Nd—Nd	CN	6†	6†
	σ^2^ (10^−3^ Å^2^)	5.3 ± 0.449	6.5 ± 2.3
	Δ*R* (Å)	0.02 ± 0.01	0.04 ± 0.04‡
	*R* (Å)	3.75 ± 0.01	3.76 ± 0.04‡
Nd—Nd	CN	6†	6†
	σ^2^ (10^−3^ Å^2^)	5.3 ± 0.4	6.5 ± 2.3
	Δ*R* (Å)	0.02 ± 0.01	0.01 ± 0.03
	*R* (Å)	3.85 ± 0.01	3.84 ± 0.03
Nd—O	CN	9†	9†
	σ^2^ (10^−3^ Å^2^)	5.3 ± 0.4	6.5 ± 2.3
	Δ*R* (Å)	0.00 ± 0.02	0.02 ± 0.03‡
	*R* (Å)	4.50 ± 0.02	4.52 ± 0.03‡
Nd—O	CN	3†	3†
	σ^2^ (10^−3^ Å^2^)	5.3 ± 0.4	6.5 ± 2.3
	Δ*R* (Å)	0.00 ± 0.02	0.02 ± 0.03
	*R* (Å)	4.66 ± 0.02	4.68 ± 0.03
Nd—O	CN	3†	3†
	σ^2^ (10^−3^ Å^2^)	9.4 ± 2.8	9.2 ± 3.8
	Δ*R* (Å)	0.00 ± 0.02	0.05 ± 0.07
	*R* (Å)	5.03 ± 0.02	5.09 ± 0.07
Nd—O	CN	6†	6†
	σ^2^ (10^−3^ Å^2^)	9.4 ± 2.8	9.2 ± 3.8
	Δ*R* (Å)	−0.00 ± 0.02	0.04 ± 0.05
	*R* (Å)	5.25 ± 0.02	5.30 ± 0.05
Nd—Nd	CN	6†	6†
	σ^2^ (10^−3^ Å^2^)	9.4 ± 2.8	9.2 ± 3.8
	Δ*R* (Å)	0.04 ± 0.03	0.03 ± 0.04‡
	*R* (Å)	5.39 ± 0.03	5.38 ± 0.04‡
All paths	Δ*E*_o_ (eV)	−8.09 ± 0.876	−7.54 ± 0.969
	S_o_^2^	1.0†	1.0†
Fit statistics	Independent points	17.5	17.5
	Number of parameters	9	9
	Reduced χ^2^	987	1500
	*R*-factor	0.012	0.019
Fit range	*k*-range (Å^−1^)	3.3–10.1	3.3–10.1
	*R*-range (Å)	1.3–5.4	1.3–5.4
Fuzziness	Distance fuzz (Å)	0.1	0.03

† Parameters fixed during the fitting.

‡ Average of nearly degenerate scattering paths for easier comparison.

**Table 5:** Comparison of the EXAFS fits before and after MEE removal from the CeO_2_*L*_3_-edge

		Original data fit	MEE removed fit
Ce—O	CN	8†	8†
	σ^2^ (10^−3^ Å^2^)	9.2 ± 1.2	6.2 ± 0.8
	Δ*R* (Å)	−0.02 ± 0.02	0.00 ± 0.01
	*R* (Å)	2.33 ± 0.02	2.35 ± 0.01
Ce—Ce	CN	12†	12†
	σ^2^ (10^−3^ Å^2^)	6.4 ± 0.8	6.4 ± 0.8
	Δ*R* (Å)	0.02 ± 0.02	0.05 ± 0.01
	*R* (Å)	3.85 ± 0.02	3.88 ± 0.01
Ce—O	CN	24†	24†
	σ^2^ (10^−3^ Å^2^)	11.6 ± 3.6	8.5 ± 2.8
	Δ*R* (Å)	−0.10 ± 0.02	−0.06 ± 0.02
	*R* (Å)	4.40 ± 0.02	4.44 ± 0.02
Ce—Ce	CN	6†	6†
	σ^2^ (10^−3^ Å^2^)	9.6 ± 1.1	9.6 ± 1.2
	Δ*R* (Å)	−0.04 ± 0.05	−0.01 ± 0.05
	*R* (Å)	5.38 ± 0.05	5.40 ± 0.05
Ce—O	CN	24†	24†
	σ^2^ (10^−3^ Å^2^)	17.4 ± 5.3	12.7 ± 4.2
	Δ*R* (Å)	0.01 ± 0.09	0.09 ± 0.06
	*R* (Å)	5.92 ± 0.09	5.99 ± 0.06
All paths	Δ*E*_o_ (eV)	6.06 ± 2.04	9.56 ± 1.59
	*S*_o_^2^	1.0†	1.0†
Fit statistics	Independent points	16.2	16.2
	Number of parameters	9	9
	Reduced χ^2^	1092	1028
	*R*-factor	0.027	0.019
Fit range	*k*-range (Å^−1^)	4.5–10.1	4.5–10.1
	*R*-range (Å)	1.5–6.1	1.5–6.1
Fuzziness	Distance fuzz (Å)	0.03	0.03

† Parameters during the fitting.

## Data Availability

Data available upon request.
