# Supplementary material for: Advanced EXAFS analysis techniques applied to the L-edges of the lanthanide oxides
Source: J Appl Crystallogr. 2024 Nov 22;57(Pt 6):1913–23. doi: 10.1107/S1600576724010240 (PMC11611292; doi:10.1107/S1600576724010240)
Supplement: Supplementary file 1 [file j-57-01913-sup1.pdf]

JOURNAL OF
APPLIED
CRYSTALLOGRAPHY

Volume 57 (2024)

Supporting information for article:

Advanced EXAFS analysis techniques applied to the *L*-edges of the lanthanide oxides

Adam Smerigan, Adam S. Hoffman, Lars Ostervold, Jiyun Hong, Jorge Perez-Aguillar, Ash C. Caine, Lauren Greenlee and Simon R. Bare

S1. Limited Information Content of the Lanthanide L-edge EXAFS

One consideration prior to collecting data at the beamline is the choice of absorption edge. The K-edge of the lanthanides requires high energy X-rays around 40-50 keV, which restricts beamline options. Further, to maintain an equivalent absorption length, more material is required for the measurement (which may be prohibitive) resulting in a less absorbing sample and noisier EXAFS data.

The L-edges (5-10 keV) of lanthanides have limited range in k-space due to the EXAFS being interrupted by the next edge. This limited k-space leads to information constraints in EXAFS fitting and poorly resolved peaks in the Fourier transform (FT). The L_3-edge of the lanthanides has a larger k-range than the L_2-edge, which increases across the series (Figure S1). The values of k-range below are the maximum possible (edge to edge); However, the first 2-3 $Å^{-1}$ will not be used in the fit due to edge effects. Depending on the concentration of lanthanides in the sample, the L_1-edge signal may be strong enough to see further into k-space. Therefore, in most situations, the L_3-edge will be the best edge to collect for EXAFS fitting.

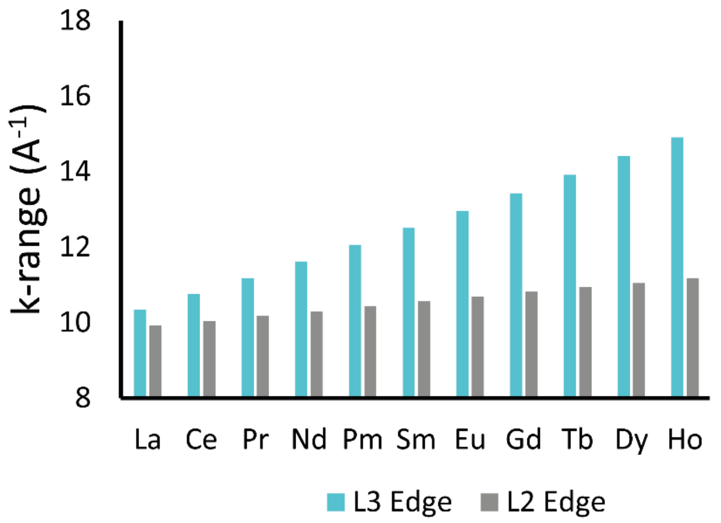

Figure S1 The k-range of the lanthanide L_2- and L_3-edges.

S2. Lanthanide Oxide EXAFS Fits

Below, the EXAFS of each of the lanthanide oxides is shown with the fit results. We hope that these can be a helpful reference when working on the EXAFS of other lanthanide compounds and lanthanide containing materials. Some figures are repeated from the main text to provide all the EXAFS in one document for ease of use.

The same methods discussed in the main text are used here. For cubic, bixbyite-type crystals (Pr, Sm-Lu), aggregation of paths was used to simplify EXAFS fits by collapsing similar length paths between both unique absorbing sites to one path. EXAFS models for all compounds used fuzzy degeneracy and the fuzziness of scattering distances is reported in Tables S6-S22 with the fit results. Cubic expansion was not used in any of the EXAFS models within the supporting information. Non-cubic expansion results are shown for Nd_2O_3 and La_2O_3. The early lanthanides La, Ce, and Pr had identifiable multielectron excitations and removal was completed for each. The before and after of the spectra are shown. The amplitude reduction factor was set to 1.0 for all fits and the coordination numbers were kept at the value defined in the structure file. Each table shows which fitting parameters were varied for each scattering path. Overall, the fitting scheme is similar for similar crystal groups (flexible models, models with many varied parameters and few constraints that can be applied to many similar compounds, often lead to more defensible fits(Calvin, 2013)). During the fitting process, we noticed that there are many Ln-O scattering paths longer than ~4 Å in the cubic bixbyite structures. The majority of these paths have small signals (weak oscillations), and their inclusion did not seem to affect the fitted parameters of the other Ln-O and Ln-Ln paths. Further, due to the limited k-range (information content) and resolution of these Ln data, these paths are difficult to resolve with any certainty, especially with the high static disorder of oxygen atoms within cubic bixbyite crystals. Depending on the specific compound, we included zero to two of these paths with zero or one extra varied parameters to account for these longer Ln-O paths (Table S1). This approach led to minor improvements in the fit but is not necessary to achieve a quality fit (R-square < 0.03) for the first two shells.

Information detailing the preparation of each sample is shown in Tables S2 an S3 below. Table S2 describes the calcination procedures used to get pure Ln oxides with references. Table S3 includes the sample preparation details along with estimated crystallite size. We estimated the average crystallite size using the Scherrer equation. We recorded the FWHM and 2θ angle of the largest peak from the XRD data, used a shape factor 0.9, and an X-ray wavelength of 0.15406 nm for all samples except Ce and Nd which were measured on synchrotron X-rays at 0.072825 nm. This calculation does

not decouple instrumental broadening from specimen broadening. Therefore, this crystallite size is an underestimate of the actual crystallite size and only serves to confirm our samples are not nanocrystals (<20 nm crystallite size) and can be considered "bulk-like" for EXAFS analysis.

Table S1 A list of Ln oxide compounds and whether single scattering paths greater than 4 Å are included in the EXAFS model of cubic bixbyite Ln oxides.

Further, the number of additional varied parameters due to the inclusion of these Ln-O paths is provided.

Compound	Number of Ln-O scattering paths > 4 Å	Number of varied parameters due to fitting these Ln-O scattering paths
La_2O_3	n/a	n/a
CeO_2	n/a	n/a
Pr_2O_3	2	1
Nd_2O_3	n/a	n/a
Sm_2O_3	0	0
Eu_2O_3	2	1
Gd_2O_3	1	0
Tb_2O_3	2	1
Dy_2O_3	1	0
Ho_2O_3	1	0
Er_2O_3	1	0
Tm_2O_3	0	0
Yb_2O_3	0	0
Lu_2O_3	1	0

Table S2 Temperature and atmosphere parameters used in the calcination of the lanthanide oxides using a Thermo Fisher Lindberg Blue M Box furnace.

Parameters were determined based upon a survey of literature for each oxide.

Compound	Ramp Rate [ºC/min]	Temperature [ºC]	Dwell time [h]	Atmosphere	Reference
La_2O_3	10	900	5	Air	(Sunding *et al.*, 2011; Neumann & Walter, 2006; Squire *et al.*, 1994; Ghiasi & Malekzadeh, 2015)
CeO_2	2	500	1.5		(Nachimuthu *et al.*, 2000; Kirk & Wood, 1995; Leoni *et al.*, 2004; Farahmandjou *et al.*, 2016; El Desouky *et al.*, 2020; Djuričić & Pickering, 1999; Chen *et al.*, 2010)
Pr_2O_3	10	600	2		*
Nd_2O_3	5	900	5		(Zawadzki & Kępiński, 2004; Ekthammathat *et al.*, 2015; Rosid *et al.*, 2019; Duhan *et al.*, 2008)
Sm_2O_3	10	700	3		(Gao *et al.*, 2003; Hussein *et al.*, 2003; Li *et al.*, 2004; Rahimi-Nasrabadi, Pourmortazavi, Aghazadeh, Ganjali, Sadeghpour Karimi *et al.*, 2017)
Eu_2O_3	10	650	3		(Kumar *et al.*, 2015; Rahimi-Nasrabadi, Pourmortazavi, Sadeghpour Karimi, Aghazadeh, Ganjali *et al.*, 2017; Yang *et al.*, 2008; Curtis & Tharp, 1959)
Gd_2O_3	10	900	3		(Whba *et al.*, 2021; Tsuzuki *et al.*, 1999; Chen *et al.*, 2016; Rudraswamy & Dhananjaya, 2012; Seo *et al.*,

					2013)
Tb_2O_3	15	800	2.5		*
Dy_2O_3	10	700	2		(Abu-Zied & Asiri, 2014; Watcharapasorn *et al.*, 2008; Rahimi-Nasrabadi, Pourmortazavi, Ganjali, Novrouzi, Faridbod *et al.*, 2017; Salavati-Niasari *et al.*, 2010; Tok *et al.*, 2006)
Ho_2O_3	10	600	1.5		(Mortazavi-Derazkola *et al.*, 2015; Abdusalyamova *et al.*, 2014; Zinatloo-Ajabshir *et al.*, 2017; Mekhemer, 2004)
Er_2O_3	10	500	1.5		(Abu-Zied *et al.*, 2016; Hussein, 2001; Li *et al.*, 2022; Azad & Maqsood, 2014)
Tm_2O_3	20	600	4		(Lee *et al.*, 2014; M. Hussein *et al.*, 2000; Sidorowicz *et al.*, 2016)
Yb_2O_3	10	900	5		(Hosokawa *et al.*, 2007; Panitz *et al.*, 1997; Rahimi-Nasrabadi, Pourmortazavi, Aghazadeh, Ganjali, Karimi *et al.*, 2017; Zhang *et al.*, 2020; Sirotinkin *et al.*, 2022; Panitz, 1999)
Lu_2O_3	5	900	2		(Zhao *et al.*, 2013; Ghosh *et al.*, 2021; An *et al.*, 2008; Riva *et al.*, 2016)

*These oxides were purchased as ampules and opened under inert atmosphere. Therefore, no calcination was performed.

Table S3 The details for sample preparation.

Compound	Diluent	Ln Oxide:Diluent Ratio	Pellet Diameter [mm]	Pellet Mass [mg]	Estimated Crystallite Size* [nm]
La_2O_3	Cellulose	1:16	7	≈20	71
CeO_2	Cellulose	1:16	7	21.0	39
Pr_2O_3	Cellulose	1:16	7	21.0	171
Nd_2O_3	Cellulose	1:16	7	23.6	46
Sm_2O_3	Cellulose	1:16	7	17.4	38
Eu_2O_3	Cellulose	1:16	7	18.2	38
Gd_2O_3	Cellulose	1:16	7	19.8	58
Tb_2O_3	Cellulose	1:16	7	20.3	50
Dy_2O_3	Cellulose	1:16	7	22.0	69
Ho_2O_3	Cellulose	1:16	7	22.8	69
Er_2O_3	Cellulose	1:16	7	24.4	40
Tm_2O_3	Cellulose	1:16	7	25.2	50
Yb_2O_3	Cellulose	1:16	7	26.8	50
Lu_2O_3	Cellulose	1:16	7	27.6	45

*Crystal size estimated using the Scherrer equation on XRD data. These values are underestimates of actual crystallite size. See Section S2 for a more detailed description.

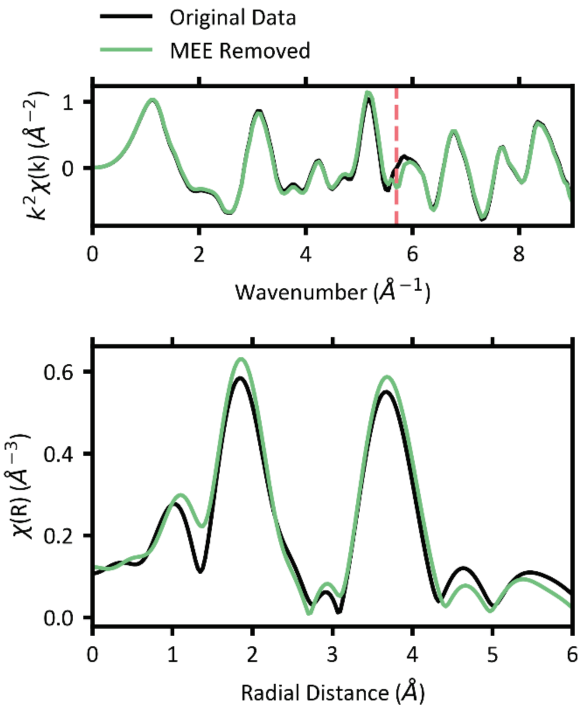

Figure S2 The k^2-weighted k-space (top) and R-space (bottom) for the L_3-edge of La_2O_3 comparing the original data to the data with the MEE removed. A vertical red line shows the location of the MEE in k-space.

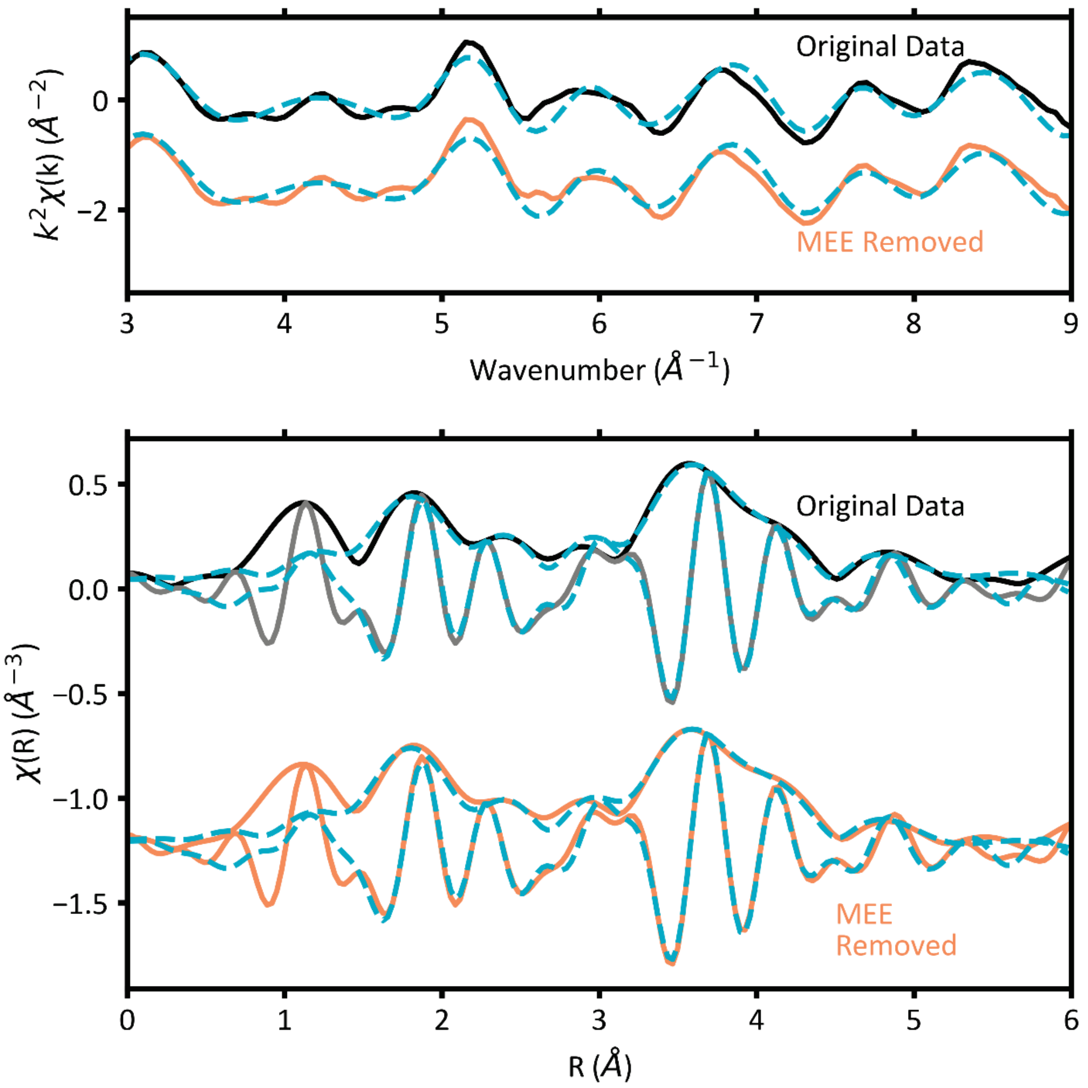

Figure S3 The k^2-weighted k-space (top) and magnitude and real part of the Fourier Transform (bottom) of the EXAFS for the La_2O_3 L_3-edge (solid black, gray, and orange lines). Fits with k-range 3.0-9.0 Å-1 and R-range 1.5-5.6 Å are represented by the blue dashed lines for both the original data and the data with the MEE removed.

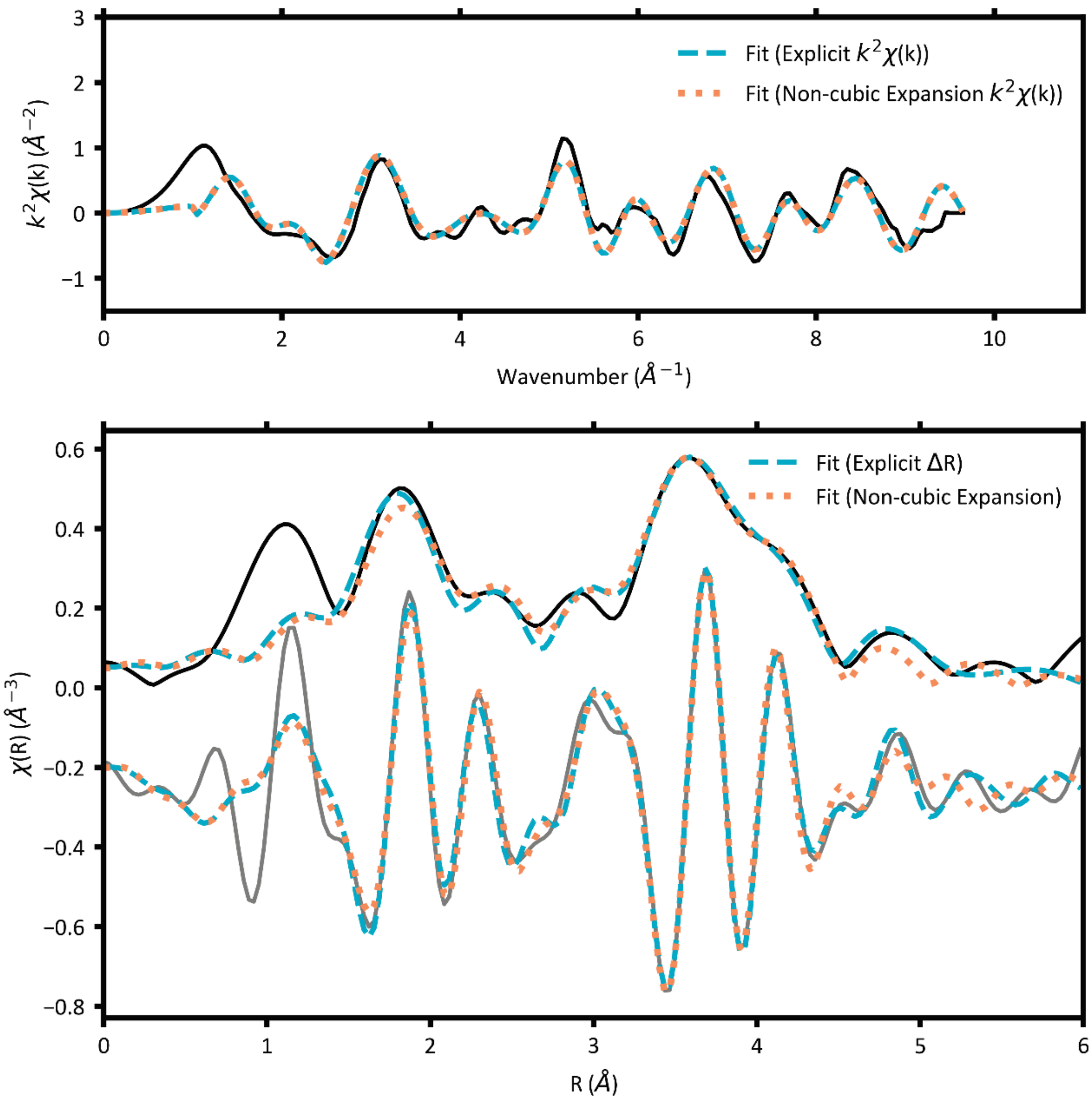

Figure S4 The k²-weighted k-space (top) and magnitude and real part of the Fourier Transform (bottom) of the EXAFS for the La_2O_3 L_3-edge (solid black and gray lines). Fits are represented by the blue dashed lines (explicit ΔR fit with k-range 3.0-9.0 and R-range 1.5-5.6) and orange dotted lines (non-cubic expansion fit with k-range 3.0-9.0 and R-range 1.45-4.6).

Table S4 Non-cubic expansion equations for the L_3-edge EXAFS of La_2O_3.

All varied parameters, set parameters, and defined parameters are shown here and used according to Table S6.

	Name	Definition
Set Parameters	S_o^2<br>a_o<br>c_o	Amplitude reduction factor (set at 1.0)<br>Initial value of the lattice constants, a and b (set at 3.937)<br>Initial value of the lattice constants, c (set at 6.132)
Varied Parameters	ΔE_o<br>ss1<br>ss2<br>ss3<br>ss4<br>Δa<br>Δc<br>ΔLa<br>ΔO1	Change in edge energy<br>MSRDs for different length paths<br>"<br>"<br>"<br>Change in lattice parameter a<br>Change in lattice parameter c<br>Distance in the c-direction of the La atom from c=0 (initial value 0.2467)<br>Distance in the c-direction of the central O atom from c=0 (initial value 0.6470)
Defined Parameters	a	$a_0 + \Delta a$
	c	$c_0 + \Delta c$
	reff	the half path length predicted by FEFF6 for each scattering path
	rLaO1.1	$\sqrt{\left(\frac{a}{3}\right)^2 + \left(\frac{a}{3}\right)^2 + \left(\left((1-\Delta O1) - \Delta La\right)\cdot c\right)^2 + 2\cdot\left(\frac{1}{3}\right)\cdot\left(\frac{1}{3}\right)\cdot a^2\cdot\cos(\frac{\pi}{3})}$
	rLaO1.3	$(\Delta O1 - \Delta La)\cdot c$
	rLaO2.1	$\sqrt{\left(\frac{a}{3}\right)^2 + \left(\frac{2a}{3}\right)^2 + (\Delta La\cdot c)^2 - 2\cdot\left(\frac{1}{3}\right)\cdot\left(\frac{2}{3}\right)\cdot a^2\cdot\cos(\frac{\pi}{3})}$
	rLaLa1.1	$\sqrt{\left(\frac{a}{3}\right)^2 + \left(\frac{a}{3}\right)^2 + (2\cdot\Delta La\cdot c)^2 + 2\cdot\left(\frac{1}{3}\right)\cdot\left(\frac{1}{3}\right)\cdot a^2\cdot\cos(\frac{\pi}{3})}$
	rLaLa1.3	$\sqrt{\left(\frac{a}{3}\right)^2 + \left(\frac{a}{3}\right)^2 + \left(\left((1-\Delta La) - \Delta La\right)\cdot c\right)^2 + 2\cdot\left(\frac{1}{3}\right)\cdot\left(\frac{1}{3}\right)\cdot a^2\cdot\cos(\frac{\pi}{3})}$
	rLaLa1.5	a
	rLaO1.5	$\sqrt{\left(\frac{4a}{3}\right)^2 + \left(\frac{2a}{3}\right)^2 + \left(\left((1-\Delta O1) - \Delta La\right)\cdot c\right)^2 - 2\cdot\left(\frac{4}{3}\right)\cdot\left(\frac{2}{3}\right)\cdot a^2\cdot\cos(\frac{\pi}{3})}$
	rLaO1.7	$\sqrt{(a)^2 + (a)^2 + \left((\Delta O1 - \Delta La)\cdot c\right)^2 - 2\cdot a^2\cdot\cos(\frac{\pi}{3})}$
	rLaO2.2	$\sqrt{\left(\frac{2a}{3}\right)^2 + \left(\frac{2a}{3}\right)^2 + (\Delta La\cdot c)^2 + 2\cdot\left(\frac{2}{3}\right)\cdot\left(\frac{2}{3}\right)\cdot a^2\cdot\cos(\frac{\pi}{3})}$

Table S5 Fitting parameters for the non-cubic expansion for the L_3-edge EXAFS of La_2O_3.

		Parameter definitions	Noncubic Expansion Fit
La-O1.1	CN	-	3[a]
	σ^2 (10^{-3} Å^2)	ss1	12.3 ± 3.61
	ΔR (Å)	rLaO1.1 - reff	0.07 ± 0.03
	R (Å)		2.44 ± 0.03
La-O1.3	CN	-	1[a]
	σ^2 (10^{-3} Å^2)	ss1	12.3 ± 3.61
	ΔR (Å)	rLaO1.3 - reff	-0.06 ± 0.06
	R (Å)		2.39 ± 0.06
La-O2.1	CN	-	3[a]
	σ^2 (10^{-3} Å^2)	ss2	5.42 ± 2.18
	ΔR (Å)	rLaO2.1 - reff	0.05 ± 0.02
	R (Å)		2.78 ± 0.02
La–La1.1	CN	-	3[a]
	σ^2 (10^{-3} Å^2)	ss3	1.36 ± 3.05
	ΔR (Å)	rLaLa1.1 - reff	0.22 ± 0.04
	R (Å)		4.00 ± 0.04
La-La 1.3	CN	-	3[a]
	σ^2 (10^{-3} Å^2)	ss3	1.36 ± 3.05
	ΔR (Å)	rLaLa1.3 - reff	0.20 ± 0.04
	R (Å)		4.05 ± 0.04
La-La 1.5	CN	-	6[a]
	σ^2 (10^{-3} Å^2)	ss3	1.36 ± 3.05
	ΔR (Å)	rLaLa1.5 - reff	-0.08 ± 0.02
	R (Å)		3.86 ± 0.02
La-O1.5	CN	-	3[a]
	σ^2 (10^{-3} Å^2)	ss3	1.36 ± 3.05
	ΔR (Å)	rLaO1.5 - reff	-0.03 ± 0.03
	R (Å)		4.57 ± 0.03
La-O1.7	CN	-	6[a]
	σ^2 (10^{-3} Å^2)	ss3	1.36 ± 3.05
	ΔR (Å)	rLaO1.7 - reff	-0.10 ± 0.04
	R (Å)		4.54 ± 0.04
La-O2.2	CN	-	3[a]
	σ^2 (10^{-3} Å^2)	ss3	1.36 ± 3.05
	ΔR (Å)	rLaO2.2 - reff	-0.03 ± 0.02
	R (Å)		4.76 ± 0.02
All Paths	ΔE_o (eV)	dE	5.78 ± 1.44
	S_o^2	-	1.0[a]
Fit Statistics	Independent Points		11.7
	Number of Parameters		8
	Reduced χ^2		679
	R-factor		0.017
Fit Range	k-range (Å^{-1})		3.0-9.0
	R-range (Å)		1.45-4.6
Fuzziness	Distance Fuzz (Å)		0.03

[a]Parameters that were fixed during fitting.

Table S6 Fitting parameters for the L_3-edge EXAFS of La_2O_3 for the 'explicit ΔR fit' with and without MEE removal.

		Varied Parameter	Explicit ΔR and Original Data	Explicit ΔR and MEE Removed
La-O	CN	-	4[a]	4[a]
	σ^2 (10^{-3} Å^2)	ss1	10.28 ± 1.71	8.74 ± 3.12
	ΔR (Å)	dr1	0.02 ± 0.01	0.01 ± 0.01
	R (Å)		2.41 ± 0.01	2.4 ± 0.01
La -O	CN	-	3[a]	3[a]
	σ^2 (10^{-3} Å^2)	ss2	5.06 ± 1.97	5.76 ± 2.08
	ΔR (Å)	dr1	0.02 ± 0.01	0.01 ± 0.01
	R (Å)		2.75 ± 0.01	2.74 ± 0.01
La - La	CN	-	6[a]	6[a]
	σ^2 (10^{-3} Å^2)	ss3	5.52 ± 1.22	6.68 ± 1.6
	ΔR (Å)	dr2	0.06 ± 0.02	0.04 ± 0.02
	R (Å)		3.88 ± 0.02	3.86 ± 0.02
La - La	CN	-	6[a]	6[a]
	σ^2 (10^{-3} Å^2)	ss4	17.34 ± 7.87	10.89 ± 3.94
	ΔR (Å)	dr2	0.06 ± 0.02	0.04 ± 0.02
	R (Å)		3.99 ± 0.02	3.97 ± 0.02
La -O	CN	-	9[a]	9[a]
	σ^2 (10^{-3} Å^2)	ss4	17.34 ± 7.87	10.89 ± 3.94
	ΔR (Å)	dr3	-0.03 ± 0.03	-0.06 ± 0.03
	R (Å)		4.6 ± 0.03	4.56 ± 0.03
La -O	CN	-	3[a]	3[a]
	σ^2 (10^{-3} Å^2)	ss4	17.34 ± 7.87	10.89 ± 3.94
	ΔR (Å)	dr3	-0.03 ± 0.03	-0.06 ± 0.03
	R (Å)		4.76 ± 0.03	4.73 ± 0.03
La -O	CN	-	3[a]	3[a]
	σ^2 (10^{-3} Å^2)	ss5	10.68 ± 4.69	14.62 ± 6.29
	ΔR (Å)	dr3	-0.03 ± 0.03	-0.06 ± 0.03
	R (Å)		5.13 ± 0.03	5.09 ± 0.03
La -O	CN	-	6[a]	6[a]
	σ^2 (10^{-3} Å^2)	ss5	10.68 ± 4.69	14.62 ± 6.29
	ΔR (Å)	dr3	-0.03 ± 0.03	-0.06 ± 0.03
	R (Å)		5.36 ± 0.03	5.33 ± 0.03
La - La	CN	-	6[a]	6[a]
	σ^2 (10^{-3} Å^2)	ss5	10.68 ± 4.69	14.62 ± 6.29
	ΔR (Å)	dr4	0.08 ± 0.04	0.06 ± 0.05
	R (Å)		5.56 ± 0.04	5.54 ± 0.05
All Paths	ΔE_o (eV)	dE	4.17 ± 0.91	3.81 ± 1.04
	S_o^2	-	1.0[a]	1.0[a]
Fit Statistics	Independent Points		15.5	15.5
	Number of Parameters		10	10
	Reduced χ^2		796	530
	R-factor		0.022	0.018
Fit Range	k-range (Å^{-1})		3.0-9.0	3.0-9.0
	R-range (Å)		1.5-5.6	1.5-5.6
Fuzziness	Distance Fuzz (Å)		0.1	0.1

[a]S_o^2 set to 1.0 and CN set to the value of the structure file during fitting.

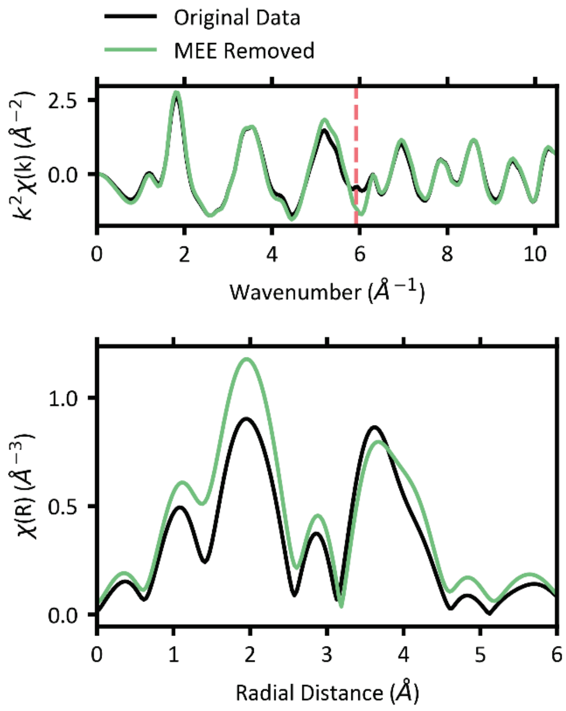

Figure S5 The k^2-weighted k-space (top) and R-space (bottom) for the L_3-edge of CeO_2 comparing the original data to the data with the MEE removed. A vertical red line shows the location of the MEE in k-space.

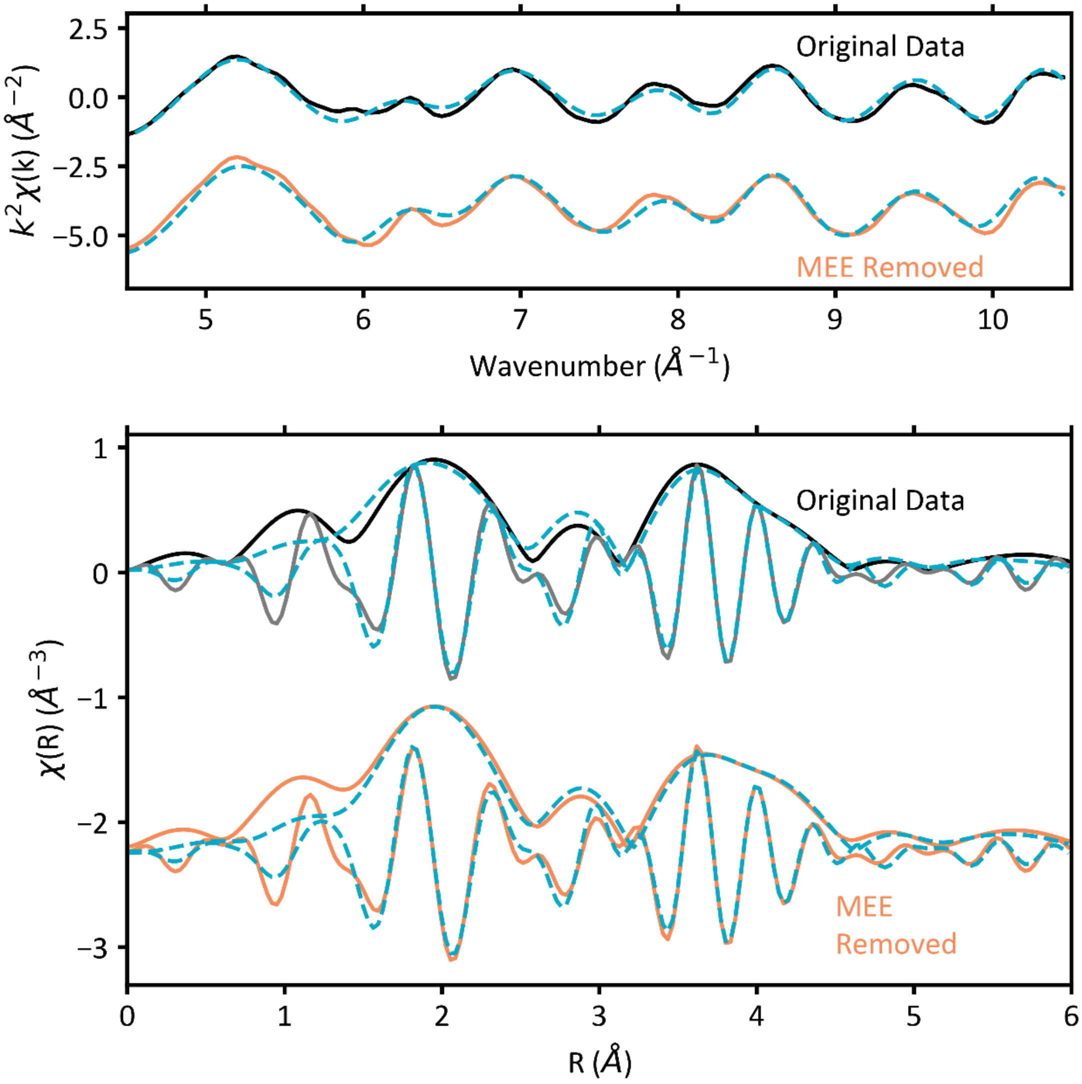

Figure S6 The k^2-weighted k-space (top) and magnitude and real part of the Fourier Transform (bottom) of the EXAFS for the CeO_2 L_3-edge (solid black, gray, and orange lines). Fits with k-range 4.5-10.1 Å-1 and R-range 1.5-6.1 Å are represented by the blue dashed lines for both the original data and the data with the MEE removed.

Table S7 Fitting parameters for the L_3-edge EXAFS of CeO_2.

		Varied Parameter	Original Data Fit	MEE Removed Fit
Ce-O	CN	-	8[a]	8[a]
	σ^2 (10^{-3} Å^2)	ss1	9.21 ± 1.17	6.21 ± 0.82
	ΔR (Å)	dr1	-0.02 ± 0.02	0.00 ± 0.01
	R (Å)		2.33 ± 0.02	2.35 ± 0.01
Ce-Ce	CN	-	12[a]	12[a]
	σ^2 (10^{-3} Å^2)	ss2	6.39 ± 0.75	6.42 ± 0.78
	ΔR (Å)	dr2	0.02 ± 0.02	0.05 ± 0.01
	R (Å)		3.85 ± 0.02	3.88 ± 0.01
Ce-O	CN	-	24[a]	24[a]
	σ^2 (10^{-3} Å^2)	ss3	11.6 ± 3.55	8.46 ± 2.77
	ΔR (Å)	dr3	-0.10 ± 0.02	-0.06 ± 0.02
	R (Å)		4.40 ± 0.02	4.44 ± 0.02
Ce-Ce	CN	-	6[a]	6[a]
	σ^2 (10^{-3} Å^2)	ss2*1.5	9.58 ± 1.13	9.63 ± 1.17
	ΔR (Å)	dr4	-0.04 ± 0.05	-0.01 ± 0.05
	R (Å)		5.38 ± 0.05	5.40 ± 0.05
Ce-O	CN	-	24[a]	24[a]
	σ^2 (10^{-3} Å^2)	ss3*1.5	17.4 ± 5.32	12.7 ± 4.16
	ΔR (Å)	dr5	0.01 ± 0.09	0.09 ± 0.06
	R (Å)		5.92 ± 0.09	5.99 ± 0.06
All Paths	ΔE_o (eV)	dE	6.06 ± 2.04	9.56 ± 1.59
	S_o^2	-	1.0[a]	1.0[a]
Fit Statistics	Independent Points		16.2	16.2
	Number of Parameters		9	9
	Reduced χ^2		1092	1028
	R-factor		0.027	0.019
Fit Range	k-range (Å^{-1})		4.5-10.1	4.5-10.1
	R-range (Å)		1.5-6.1	1.5-6.1
Fuzziness	Distance Fuzz (Å)		0.03	0.03

[a]S_o^2 set to 1.0 and CN set to the value of the structure file during fitting.

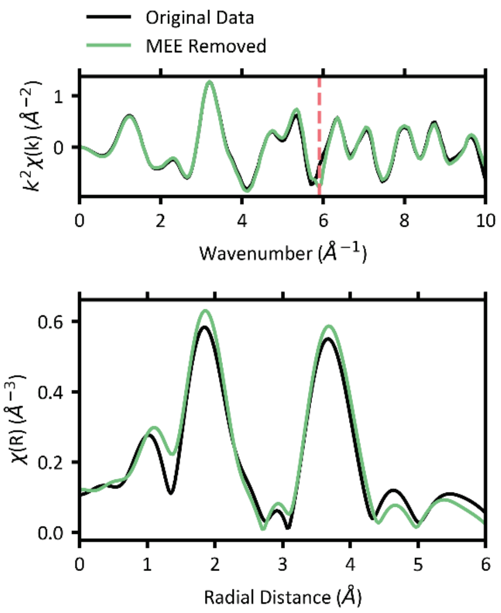

Figure S7 The k^2-weighted k-space (top) and R-space (bottom) for the L_2-edge of Pr_2O_3 comparing the original data to the data with the MEE removed. A vertical red line shows the location of the MEE in k-space.

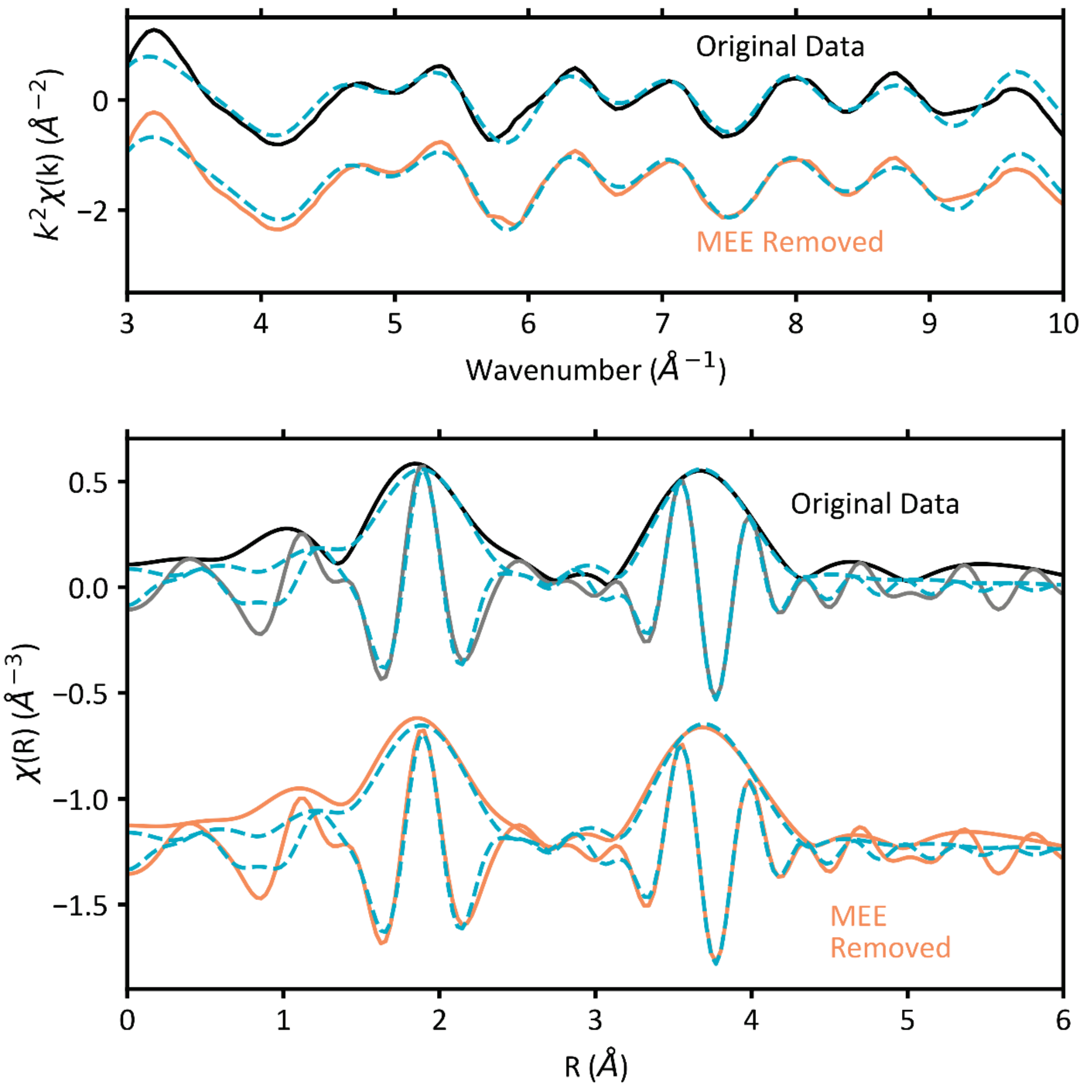

Figure S8 The k^2-weighted k-space (top) and magnitude and real part of the Fourier Transform (bottom) of the EXAFS for the Pr_2O_3 L_2-edge (solid black, gray, and orange lines). Fits with k-range 3.6-9.5 Å-1 and R-range 1.45-4.9 Å are represented by the blue dashed lines for both the original data and the data with the MEE removed.

Table S8 Fitting parameters for the L_2-edge EXAFS of Pr_2O_3.

		Varied Parameter	Original Data	MEE Removed
Pr-O	CN	-	4.5	4.5
	σ^2 (10^{-3} Å^2)	ss1	8.60 ± 1.72	7.88 ± 1.27
	ΔR (Å)	dr1	0.07 ± 0.03	0.07 ± 0.02
	R (Å)		2.4 ± 0.03	2.39 ± 0.02
Pr-O	CN	-	1.5	1.5
	σ^2 (10^{-3} Å^2)	ss1	8.60 ± 1.72	7.88 ± 1.27
	ΔR (Å)	dr1	0.07 ± 0.03	0.07 ± 0.02
	R (Å)		2.73 ± 0.03	2.72 ± 0.02
Pr-Pr	CN	-	6	6
	σ^2 (10^{-3} Å^2)	ss2	6.71 ± 2.04	6.57 ± 1.48
	ΔR (Å)	dr2	0.08 ± 0.03	0.08 ± 0.02
	R (Å)		3.77 ± 0.03	3.77 ± 0.02
Pr-Pr	CN	-	6	6
	σ^2 (10^{-3} Å^2)	ss3	22.17 ± 25.28	21.25 ± 11.93
	ΔR (Å)	dr3	-0.08 ± 0.07	-0.02 ± 0.15
	R (Å)		4.16 ± 0.07	4.22 ± 0.15
Pr-O	CN	-	6	6
	σ^2 (10^{-3} Å^2)	ss*1.5	12.9 ± 2.58	11.82 ± 1.9
	ΔR (Å)	dr4	0.11 ± 0.14	0.06 ± 0.13
	R (Å)		4.48 ± 0.14	4.43 ± 0.13
Pr-O	CN	-	3	3
	σ^2 (10^{-3} Å^2)	ss*1.5	12.9 ± 2.58	11.82 ± 1.9
	ΔR (Å)	dr4	0.11 ± 0.14	0.06 ± 0.13
	R (Å)		4.64 ± 0.14	4.59 ± 0.13
All Paths	ΔE_o (eV)	dE	4.49 ± 3.83	4.78 ± 2.79
	S_o^2	-	1.0 [a]	1.0 [a]
Fit Statistics	Independent Points		12.7	12.7
	Number of Parameters		8	8
	Reduced χ^2		2286	1208
	R-factor		0.036	0.020
Fit Range	k-range (Å^{-1})		3.6-9.5	3.6-9.5
	R-range (Å)		1.45-4.9	1.45-4.9
Fuzziness	Distance Fuzz (Å)		0.1	0.1

[a] S_o^2 set to 1.0 and CN set to the value of the structure file during fitting.

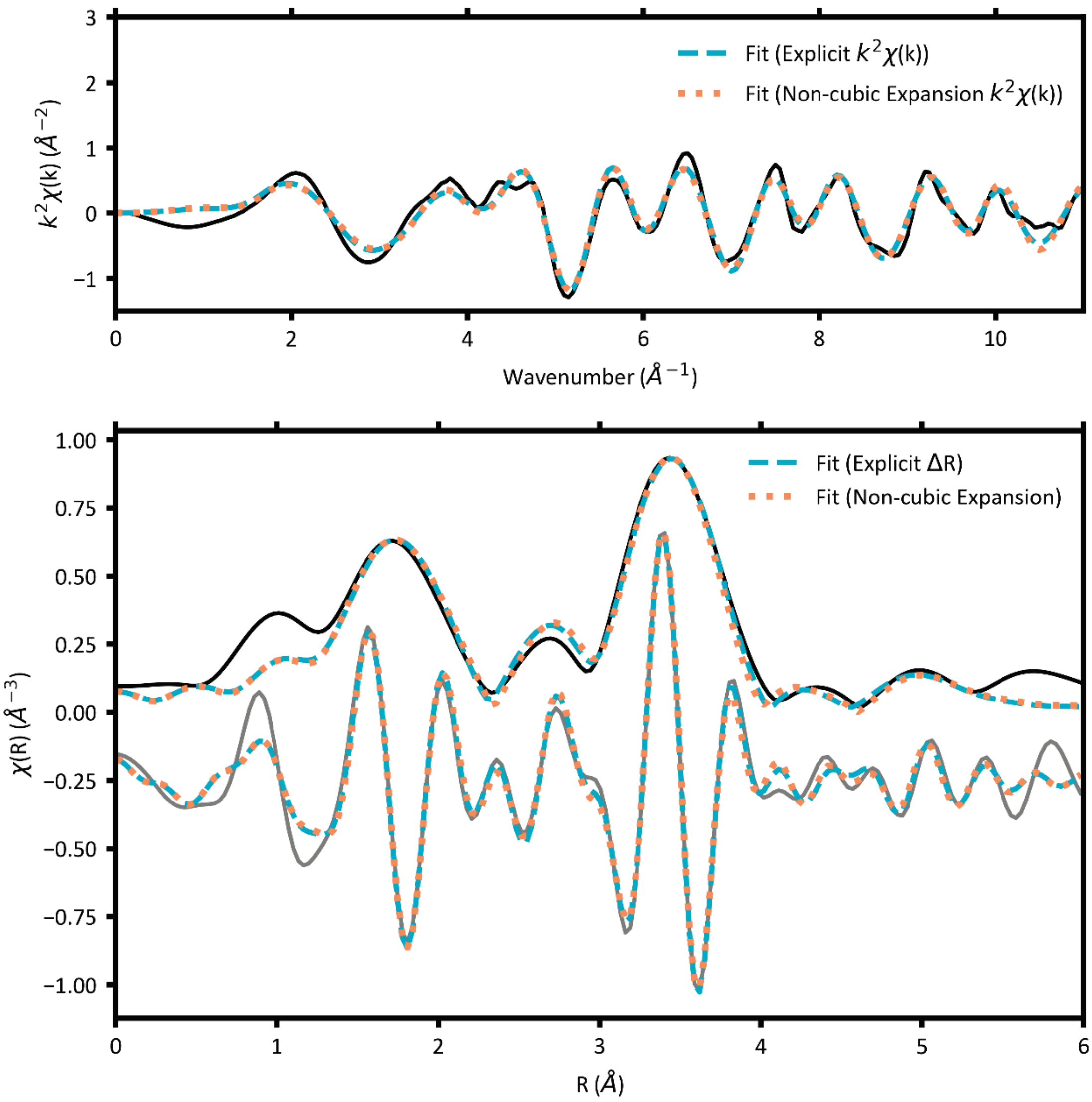

Figure S9 The k[2]-weighted k-space (top) and magnitude and real part of the Fourier Transform (bottom) of the EXAFS for the Nd_2O_3 L_1-edge (solid black and gray lines). Fits with k-range 3.3-10.1 Å-1 and R-range 1.3-5.4 Å are represented by the blue dashed (explicit ΔR fit) and orange dotted (non-cubic expansion fit) lines.

Table S9 Non-cubic expansion equations for the L_1-edge EXAFS of Nd_2O_3.

All varied parameters, set parameters, and defined parameters are shown here and used according to Table S11.

	Name	Definition
Set Parameters	S_o^2	Amplitude reduction factor (set at 1.0)
	a_o	Initial value of the lattice constants, a and b (set at 3.831)
	c_o	Initial value of the lattice constants, c (set at 5.999)
Varied Parameters	ΔE_o	Change in edge energy
	ss1	MSRDs for different length paths
	ss2	"
	ss3	"
	ss4	"
	Δa	Change in lattice parameter a
	Δc	Change in lattice parameter c
	ΔNd	Distance in the c-direction of the Nd atom from c=0 (initial value 0.2462)
	ΔO1	Distance in the c-direction of the central O atom from c=0 (initial value 0.6466)
Defined Parameters	a	$a_0 + \Delta a$
	c	$c_0 + \Delta c$
	reff	the half path length predicted by FEFF6 for each scattering path
	rNdO1.1	$\sqrt{\left(\frac{a}{3}\right)^2 + \left(\frac{a}{3}\right)^2 + \left(\left((1-\Delta O1) - \Delta Nd\right)\cdot c\right)^2 + 2\cdot\left(\frac{1}{3}\right)\cdot\left(\frac{1}{3}\right)\cdot a^2\cdot\cos(\frac{\pi}{3})}$
	rNdO1.3	$(\Delta O1 - \Delta Nd)\cdot c$
	rNdO2.1	$\sqrt{\left(\frac{a}{3}\right)^2 + \left(\frac{2a}{3}\right)^2 + (\Delta Nd\cdot c)^2 - 2\cdot\left(\frac{1}{3}\right)\cdot\left(\frac{2}{3}\right)\cdot a^2\cdot\cos(\frac{\pi}{3})}$
	rNdNd1.1	$\sqrt{\left(\frac{a}{3}\right)^2 + \left(\frac{a}{3}\right)^2 + (2\cdot\Delta Nd\cdot c)^2 + 2\cdot\left(\frac{1}{3}\right)\cdot\left(\frac{1}{3}\right)\cdot a^2\cdot\cos(\frac{\pi}{3})}$
	rNdNd1.3	$\sqrt{\left(\frac{a}{3}\right)^2 + \left(\frac{a}{3}\right)^2 + \left(\left((1-\Delta \mathrm{Nd}) - \Delta Nd\right)\cdot c\right)^2 + 2\cdot\left(\frac{1}{3}\right)\cdot\left(\frac{1}{3}\right)\cdot a^2\cdot\cos(\frac{\pi}{3})}$
	rNdNd1.5	a
	rNdO1.5	$\sqrt{\left(\frac{4a}{3}\right)^2 + \left(\frac{2a}{3}\right)^2 + \left(\left((1-\Delta O1) - \Delta Nd\right)\cdot c\right)^2 - 2\cdot\left(\frac{4}{3}\right)\cdot\left(\frac{2}{3}\right)\cdot a^2\cdot\cos(\frac{\pi}{3})}$
	rNdO1.7	$\sqrt{(a)^2 + (a)^2 + \left((\Delta O1 - \Delta Nd)\cdot c\right)^2 - 2\cdot a^2\cdot\cos(\frac{\pi}{3})}$
	rNdO2.2	$\sqrt{\left(\frac{2a}{3}\right)^2 + \left(\frac{2a}{3}\right)^2 + (\Delta Nd\cdot c)^2 + 2\cdot\left(\frac{2}{3}\right)\cdot\left(\frac{2}{3}\right)\cdot a^2\cdot\cos(\frac{\pi}{3})}$
	rNdO2.5	$\sqrt{\left(\frac{a}{3}\right)^2 + \left(\frac{2a}{3}\right)^2 + ((1-\Delta Nd)\cdot c)^2 - 2\cdot\left(\frac{1}{3}\right)\cdot\left(\frac{2}{3}\right)\cdot a^2\cdot\cos(\frac{\pi}{3})}$
	rNdO1.8	$\sqrt{(a)^2 + (a)^2 + \left(\left((1-\Delta O1) + \Delta Nd\right)\cdot c\right)^2 - 2\cdot a^2\cdot\cos(\frac{\pi}{3})}$
	rNdNd1.6	$\sqrt{\left(\frac{4a}{3}\right)^2 + \left(\frac{2a}{3}\right)^2 + (2\cdot\Delta Nd\cdot c)^2 - 2\cdot\left(\frac{4}{3}\right)\cdot\left(\frac{2}{3}\right)\cdot a^2\cdot\cos(\frac{\pi}{3})}$

	rNdNd1.8	$\sqrt{\left(\frac{4a}{3}\right)^2+\left(\frac{2a}{3}\right)^2+\left(((1-\Delta \mathrm{Nd})-\Delta Nd)\cdot c\right)^2-2\cdot\left(\frac{4}{3}\right)\cdot\left(\frac{2}{3}\right)\cdot a^2\cdot\cos(\frac{\pi}{3})}$

Table S10 Fitting parameters for the 'noncubic expansion' fit for the L_1-edge EXAFS of Nd_2O_3.

		Parameter definitions	Noncubic Expansion Fit
Nd-O1.1	CN	-	3[a]
	σ² (10⁻³ Å²)	ss1	0.92 ± 1.54
	ΔR (Å)	rNdO1.1 - reff	0.00 ± 0.02
	R (Å)		2.31 ± 0.02
Nd-O1.3	CN	-	1[a]
	σ² (10⁻³ Å²)	ss1	0.92 ± 1.54
	ΔR (Å)	rNdO1.3 - reff	0.03 ± 0.06
	R (Å)		2.44 ± 0.06
Nd-O2.1	CN	-	3[a]
	σ² (10⁻³ Å²)	ss2	12.98 ± 4.23
	ΔR (Å)	rNdO2.1 - reff	0.02 ± 0.02
	R (Å)		2.68 ± 0.02
Nd-Nd1.1	CN	-	3[a]
	σ² (10⁻³ Å²)	ss3	6.50 ± 2.25
	ΔR (Å)	rNdNd1.1 - reff	0.05 ± 0.04
	R (Å)		3.74 ± 0.04
Nd-Nd1.3	CN	-	3[a]
	σ² (10⁻³ Å²)	ss3	6.50 ± 2.25
	ΔR (Å)	rNdNd1.3 - reff	0.02 ± 0.05
	R (Å)		3.79 ± 0.05
Nd-Nd1.5	CN	-	6[a]
	σ² (10⁻³ Å²)	ss3	6.50 ± 2.25
	ΔR (Å)	rNdNd1.5 - reff	0.01 ± 0.03
	R (Å)		3.84 ± 0.03
Nd-O1.5	CN	-	3[a]
	σ² (10⁻³ Å²)	ss3	6.50 ± 2.25
	ΔR (Å)	rNdO1.5 - reff	0.01 ± 0.03
	R (Å)		4.48 ± 0.03
Nd-O1.7	CN	-	6[a]
	σ² (10⁻³ Å²)	ss3	6.50 ± 2.25
	ΔR (Å)	rNdO1.7 - reff	0.03 ± 0.04
	R (Å)		4.55 ± 0.04
Nd-O2.2	CN	-	3[a]
	σ² (10⁻³ Å²)	ss3	6.50 ± 2.25

	ΔR (Å)	rNdO2.2 - reff	0.02 ± 0.03
	R (Å)		4.68 ± 0.03
Nd-O2.5	CN	-	3[a]
	σ^2 (10^{-3} Å^2)	ss4	9.23 ± 3.76
	ΔR (Å)	rNdO2.5 - reff	0.05 ± 0.07
	R (Å)		5.09 ± 0.07
Nd-O1.8	CN	-	6[a]
	σ^2 (10^{-3} Å^2)	ss4	9.23 ± 3.76
	ΔR (Å)	rNdO1.8 - reff	0.04 ± 0.05
	R (Å)		5.30 ± 0.05
Nd-Nd1.6	CN	-	3[a]
	σ^2 (10^{-3} Å^2)	ss4	9.23 ± 3.76
	ΔR (Å)	rNdNd1.6 - reff	0.04 ± 0.04
	R (Å)		5.36 ± 0.04
Nd-Nd1.8	CN	-	3[a]
	σ^2 (10^{-3} Å^2)	ss4	9.23 ± 3.76
	ΔR (Å)	rNdNd1.8 - reff	0.02 ± 0.04
	R (Å)		5.39 ± 0.04
All Paths	ΔE_o (eV)	dE	-7.54 ± 0.969
	S_o^2	-	1.0 [a]
Fit Statistics	Independent Points		17.5
	Number of Parameters		9
	Reduced χ^2		1500
	R-factor		0.019
Fit Range	k-range (Å^{-1})		3.3-10.1
	R-range (Å)		1.3 – 5.4
Fuzziness	Distance Fuzz (Å)		0.03

[a]Parameters that were fixed during fitting.

Table S11 Fitting parameters for the 'explicit ΔR' fit for the L_1-edge EXAFS of Nd_2O_3.

		Varied Parameters	Explicit ΔR Fit
Nd-O	CN	-	4 [a]
	σ^2 (10^{-3} Å^2)	ss1	3.56 ± 0.670
	ΔR (Å)	dr1	0.00 ± 0.02
	R (Å)		2.32 ± 0.02
Nd-O	CN	-	3 [a]
	σ^2 (10^{-3} Å^2)	ss2	12.6 ± 3.40
	ΔR (Å)	dr1	0.00 ± 0.02
	R (Å)		2.66 ± 0.02
Nd-Nd	CN	-	6 [a]
	σ^2 (10^{-3} Å^2)	ss3	5.34 ± 0.449
	ΔR (Å)	dr2	0.02 ± 0.01
	R (Å)		3.75 ± 0.01
Nd-Nd	CN	-	6 [a]
	σ^2 (10^{-3} Å^2)	ss3	5.34 ± 0.449
	ΔR (Å)	dr2	0.02 ± 0.01
	R (Å)		3.85 ± 0.01
Nd-O	CN	-	9 [a]
	σ^2 (10^{-3} Å^2)	ss3	5.34 ± 0.449
	ΔR (Å)	dr	0.00 ± 0.02
	R (Å)		4.50 ± 0.02
Nd-O	CN	-	3 [a]
	σ^2 (10^{-3} Å^2)	ss3	5.34 ± 0.449
	ΔR (Å)	dr	0.00 ± 0.02
	R (Å)		4.66 ± 0.02
Nd-O	CN	-	3 [a]
	σ^2 (10^{-3} Å^2)	ss4	9.44 ± 2.79
	ΔR (Å)		0.00 ± 0.02
	R (Å)		5.03 ± 0.02
Nd-O	CN	-	6 [a]
	σ^2 (10^{-3} Å^2)	ss4	9.44 ± 2.79
	ΔR (Å)		-0.00 ± 0.02
	R (Å)		5.25 ± 0.02
Nd-Nd	CN	-	6 [a]
	σ^2 (10^{-3} Å^2)	ss4	9.44 ± 2.79
	ΔR (Å)		0.04 ± 0.03
	R (Å)		5.39 ± 0.03
All Paths	ΔE_o (eV)	dE	-7.54 ± 0.969
	S_o^2	-	1.0 [a]
Fit Statistics	Independent Points		17.5
	Number of Parameters		9
	Reduced χ^2		987
	R-factor		0.012
Fit Range	k-range (Å^{-1})		3.3-10.1
	R-range (Å)		1.3 – 5.4
Fuzziness	Distance Fuzz (Å)		0.1

[a]Parameters that were fixed during fitting.

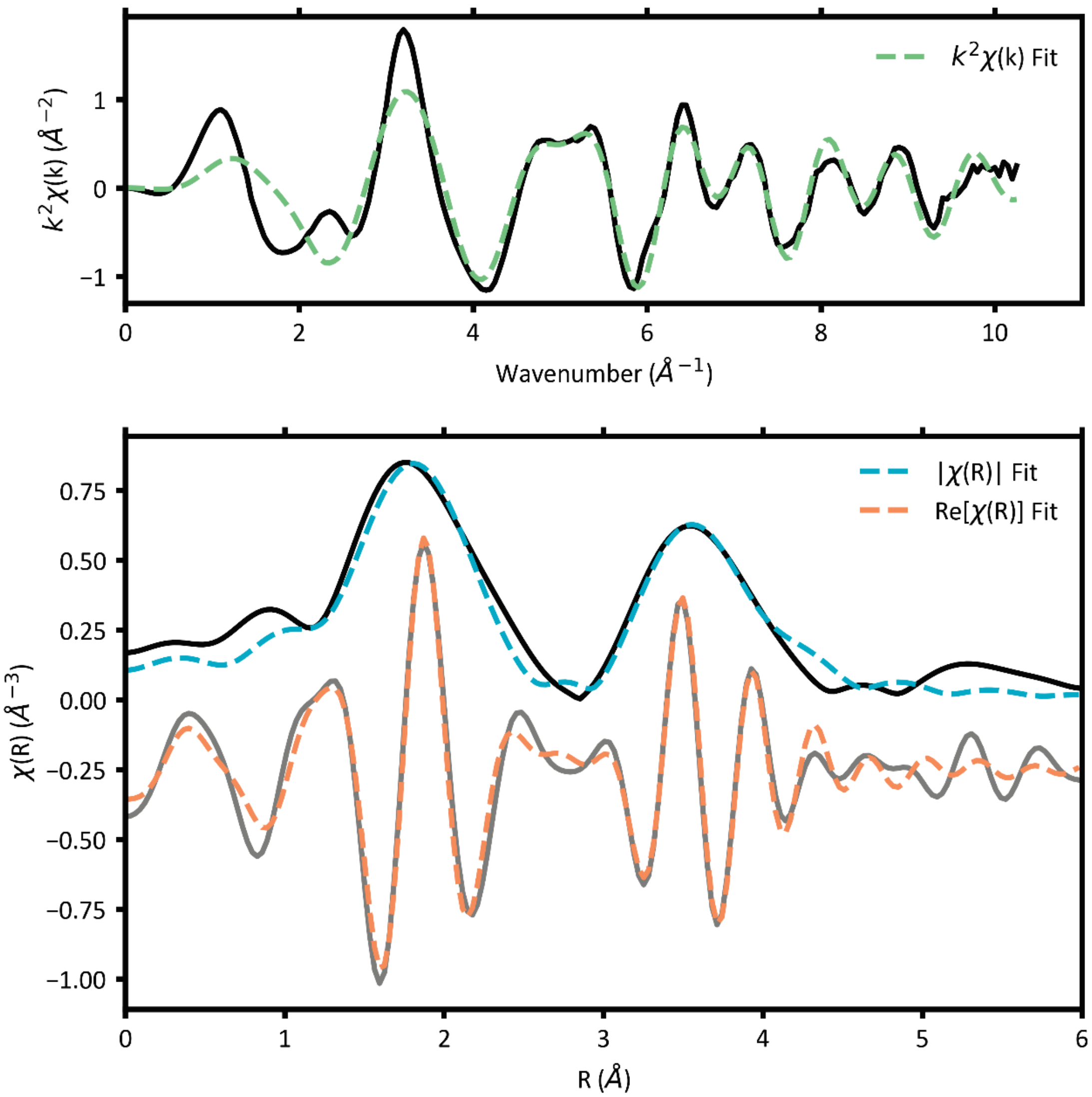

Figure S10 The k^2-weighted k-space (top) and magnitude and real part of the Fourier Transform (bottom) of the EXAFS for the Sm_2O_3 L_2-edge (solid black and gray lines). Fits with k-range 3.6-9.1 Å-1 and R-range 1.3-4.3 Å are represented by the colored dashed lines.

Table S12 Fitting parameters for the L_2-edge EXAFS of Sm_2O_3.

		Varied Parameter	Fit Results
Sm-O	CN	-	4.5[a]
	σ^2 (10^{-3} Å^2)	ss1	6.75 ± 1.22
	ΔR (Å)	dr1	-0.04 ± 0.02
	R (Å)		2.29 ± 0.02
Sm-O	CN	-	1.5[a]
	σ^2 (10^{-3} Å^2)	ss1	6.75 ± 1.22
	ΔR (Å)	dr1	-0.04 ± 0.02
	R (Å)		2.38 ± 0.02
Sm-Sm	CN	-	6.0[a]
	σ^2 (10^{-3} Å^2)	ss2	7.15 ± 1.17
	ΔR (Å)	dr2	0.02 ± 0.02
	R (Å)		3.66 ± 0.02
Sm-Sm	CN	-	6.0[a]
	σ^2 (10^{-3} Å^2)	ss2	7.15 ± 1.17
	ΔR (Å)	dr3	-0.04 ± 0.03
	R (Å)		4.08 ± 0.03
All Paths	ΔE_o (eV)	dE	-0.44 ± 1.85
	S_o^2	-	1.0[a]
Fit Statistics	Independent Points		10.3
	Number of Parameters		6
	Reduced χ^2		730.2
	R-factor		0.020
Fit Range	k-range (Å^{-1})		3.6-9.1
	R-range (Å)		1.3-4.3
Fuzziness	Distance Fuzz (Å)		0.10

[a]S_o^2 set to 1.0 and CN set to the value of the structure file during fitting.

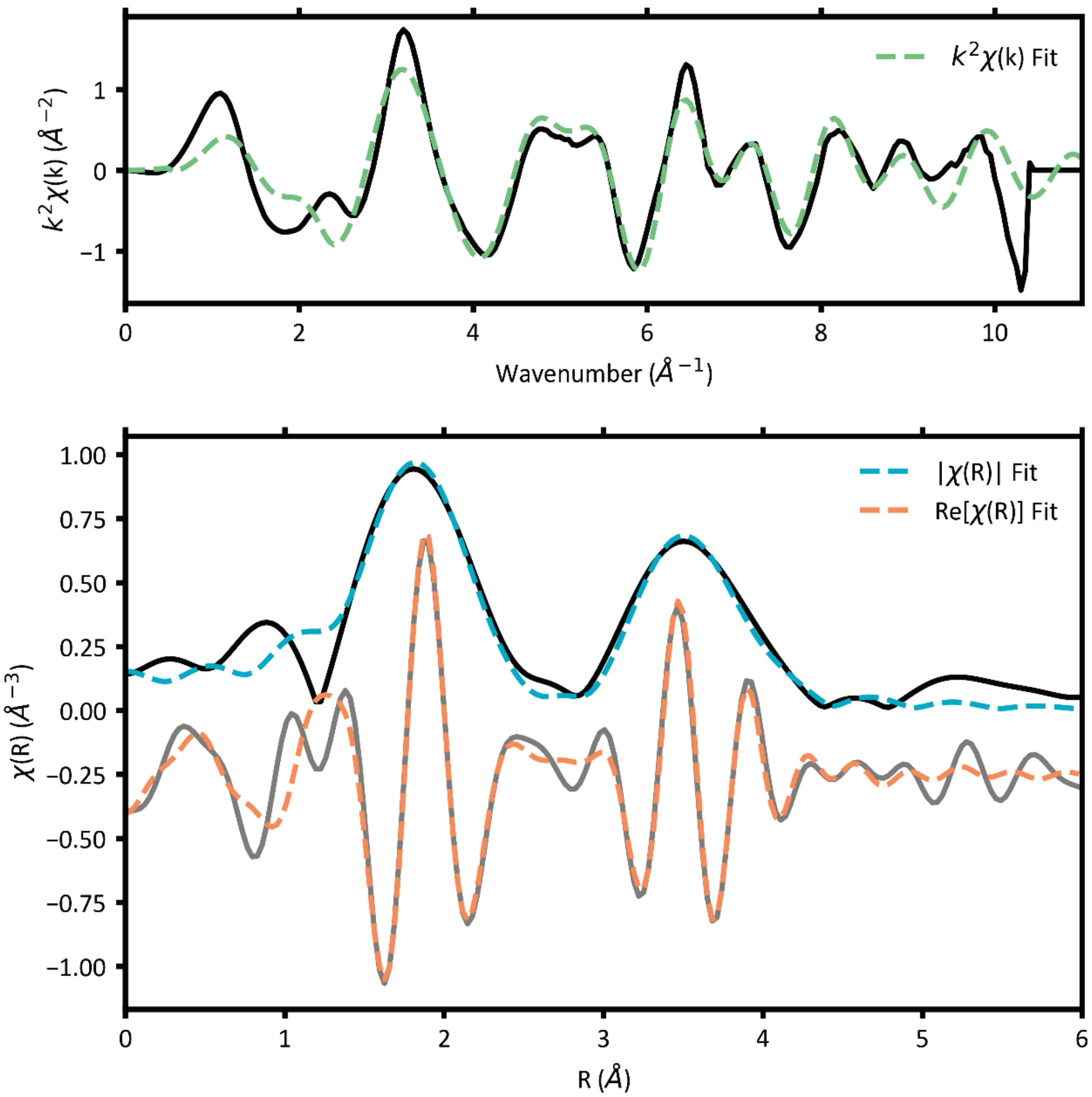

Figure S11 The k[2]-weighted k-space (top) and magnitude and real part of the Fourier Transform (bottom) of the EXAFS for the Eu_2O_3 L_2-edge (solid black and gray lines). Fits with k-range 3.6-9.5 Å-1 and R-range 1.3-4.7 Å are represented by the colored dashed lines.

Table S13 Fitting parameters for the L_2-edge EXAFS of Eu_2O_3.

		Varied Parameter	Fit Results
Eu-O	CN	-	6
	σ^2 (10^{-3} Å^2)	ss1	6.89 ± 1.09
	ΔR (Å)	dr1	-0.04 ± 0.02
	R (Å)		2.31 ± 0.02
Eu-Eu	CN	-	6
	σ^2 (10^{-3} Å^2)	ss2	6.31 ± 3.05
	ΔR (Å)	dr2	0.00 ± 0.03
	R (Å)		3.62 ± 0.03
Eu-Eu	CN	-	6
	σ^2 (10^{-3} Å^2)	ss3	12.82 ± 8.44
	ΔR (Å)	dr3	-0.03 ± 0.09
	R (Å)		4.06 ± 0.09
Eu-O	CN	-	4.5
	σ^2 (10^{-3} Å^2)	ss*1.5	10.34 ± 1.63
	ΔR (Å)	dr4	-0.22 ± 0.18
	R (Å)		3.99 ± 0.18
Eu-O	CN	-	7.5
	σ^2 (10^{-3} Å^2)	ss*1.5	10.34 ± 1.63
	ΔR (Å)	dr4	-0.22 ± 0.18
	R (Å)		4.32 ± 0.18
All Paths	ΔE_o (eV)	dE	0.98 ± 2.29
	S_o^2	-	1.0 [a]
Fit Statistics	Independent Points		12.6
	Number of Parameters		8
	Reduced χ^2		1392
	R-factor		0.019
Fit Range	k-range (Å^{-1})		3.6-9.5
	R-range (Å)		1.3-4.7
Fuzziness	Distance Fuzz (Å)		0.10

[a] S_o^2 set to 1.0 and CN set to the value of the structure file during fitting.

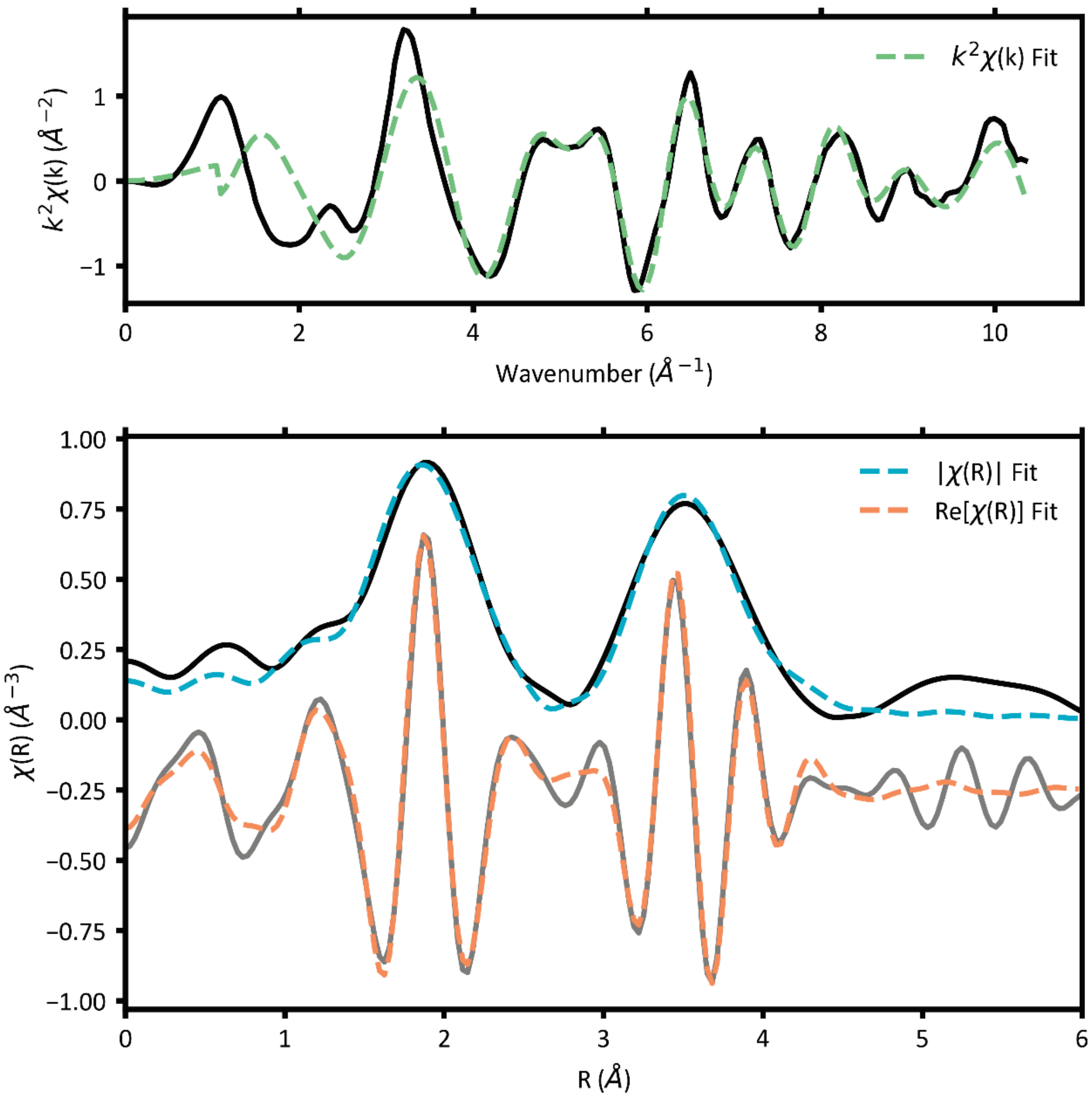

Figure S12 The k^2-weighted k-space (top) and magnitude and real part of the Fourier Transform (bottom) of the EXAFS for the Gd_2O_3 L_2-edge (solid black and gray lines). Fits with k-range 3.65-9.1 Å-1 and R-range 1.3-4.2 Å are represented by the colored dashed lines.

Table S14 Fitting parameters for the L_2-edge EXAFS of Gd_2O_3.

		Varied Parameter	Fit Results
Gd-O	CN	-	4.5[a]
	σ^2 (10^{-3} Å^2)	ss1	5.9 ± 1.14
	ΔR (Å)	dr1	0.01 ± 0.02
	R (Å)		2.31 ± 0.02
Gd-O	CN	-	1.5[a]
	σ^2 (10^{-3} Å^2)	ss1	5.9 ± 1.14
	ΔR (Å)	dr1	0.01 ± 0.02
	R (Å)		2.42 ± 0.02
Gd-Gd	CN	-	6[a]
	σ^2 (10^{-3} Å^2)	ss2	4.12 ± 1.32
	ΔR (Å)	dr2	0.03 ± 0.02
	R (Å)		3.62 ± 0.02
Gd-O	CN	-	1.5[a]
	σ^2 (10^{-3} Å^2)	ss3	6.23 ± 2.53
	ΔR (Å)	dr3	0.02 ± 0.03
	R (Å)		4.05 ± 0.03
Gd-Gd	CN	-	6[a]
	σ^2 (10^{-3} Å^2)	ss3	6.23 ± 2.53
	ΔR (Å)	dr3	0.02 ± 0.03
	R (Å)		4.11 ± 0.03
All Paths	ΔE_o (eV)	dE	4.53 ± 1.64
	S_o^2	-	1.0[a]
Fit Statistics	Independent Points		10.9
	Number of Parameters		7
	Reduced χ^2		1340
	R-factor		0.014
Fit Range	k-range (Å^{-1})		3.65-9.7
	R-range (Å)		1.3-4.2
Fuzziness	Distance Fuzz (Å)		0.10

[a] S_o^2 set to 1.0 and CN set to the value of the structure file during fitting.

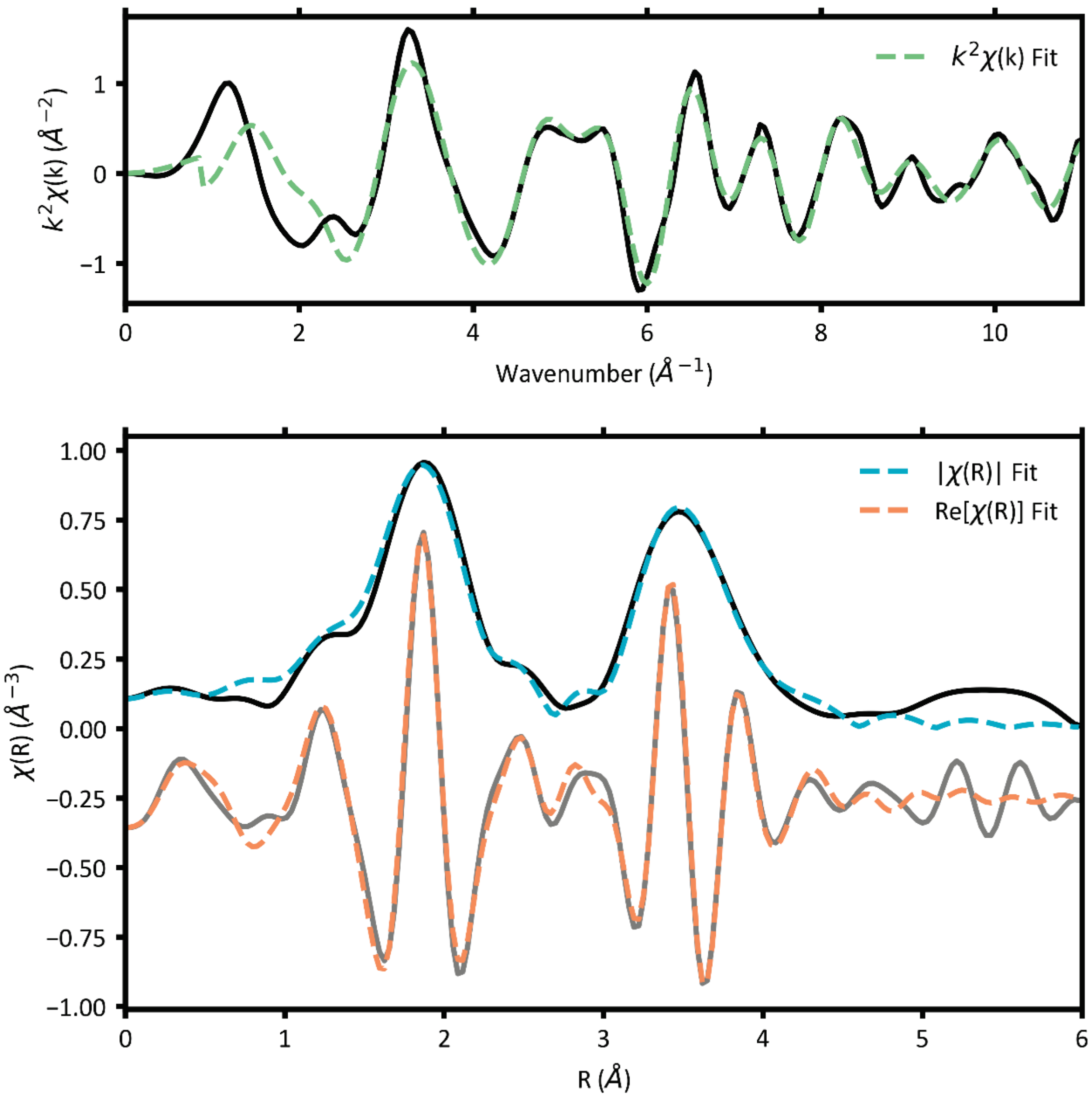

Figure S13 The k^2-weighted k-space (top) and magnitude and real part of the Fourier Transform (bottom) of the EXAFS for the Tb_2O_3 L_3-edge (solid black and gray lines). Fits with k-range 3.65-10.5 Å-1 and R-range 1.3-4.7 Å are represented by the colored dashed lines.

Table S15 Fitting parameters for the L_3-edge EXAFS of Tb_2O_3.

		Varied Parameter	Fit Results
Tb-O	CN	-	6 [a]
	σ^2 (10^{-3} Å^2)	ss1	8.24 ± 0.81
	ΔR (Å)	dr1	0.00 ± 0.01
	R (Å)		2.30 ± 0.01
Tb-Tb	CN	-	6 [a]
	σ^2 (10^{-3} Å^2)	ss2	5.02 ± 0.85
	ΔR (Å)	dr2	0.02 ± 0.01
	R (Å)		3.59 ± 0.01
Tb-Tb	CN	-	6 [a]
	σ^2 (10^{-3} Å^2)	ss3	7.30 ± 2.00
	ΔR (Å)	dr3	0.04 ± 0.02
	R (Å)		4.07 ± 0.02
Tb-O	CN	-	1.5 [a]
	σ^2 (10^{-3} Å^2)	ss1*1.5	12.37 ± 1.22
	ΔR (Å)	dr4	-0.14 ± 0.06
	R (Å)		3.97 ± 0.06
Tb-O	CN	-	6 [a]
	σ^2 (10^{-3} Å^2)	ss1*1.5	12.37 ± 1.22
	ΔR (Å)	dr4	-0.14 ± 0.06
	R (Å)		4.21 ± 0.06
All Paths	ΔE_o (eV)	dE	2.76 ± 1.43
	S_o^2	-	1.0[a]
Fit Statistics	Independent Points		14.6
	Number of Parameters		8
	Reduced χ^2		1555
	R-factor		0.013
Fit Range	k-range (Å^{-1})		3.65-10.5
	R-range (Å)		1.3-4.7
Fuzziness	Distance Fuzz (Å)		0.15

[a]S_o^2 set to 1.0 and CN set to the value of the structure file during fitting.

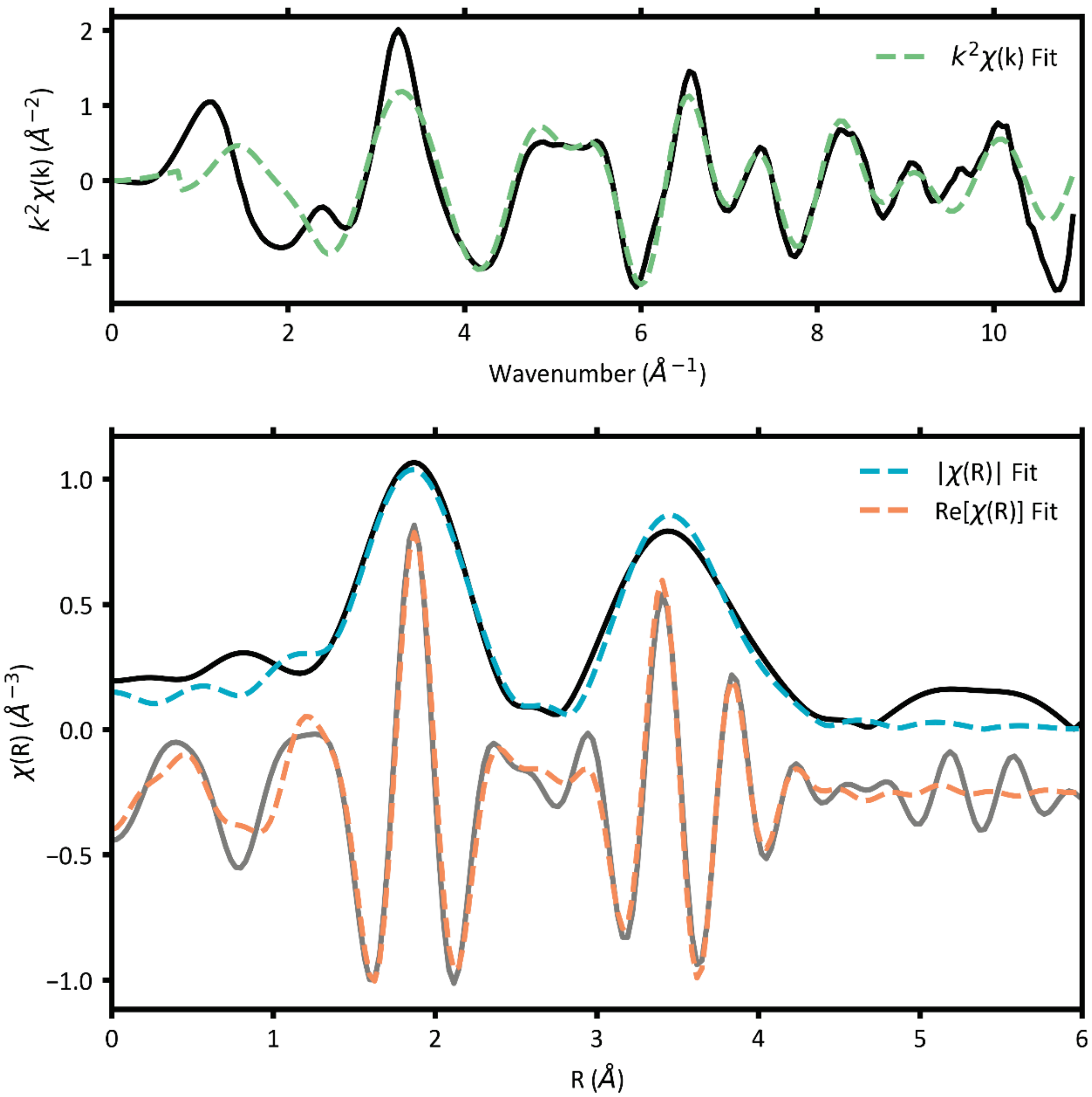

Figure S14 The k²-weighted k-space (top) and magnitude and real part of the Fourier Transform (bottom) of the EXAFS for the Dy_2O_3 L_2-edge (solid black and gray lines). Fits with k-range 3.65-9.76 Å-1 and R-range 1.3-4.2 Å are represented by the colored dashed lines.

Table S16 Fitting parameters for the L_2-edge EXAFS of Dy_2O_3.

		Varied Parameter	Fit Results
Dy-O	CN	-	6[a]
	σ^2 (10^{-3} Å^2)	ss1	5.74 ± 0.90
	ΔR (Å)	dr1	0.01 ± 0.00
	R (Å)		2.29 ± 0.00
Dy-Dy	CN	-	6[a]
	σ^2 (10^{-3} Å^2)	ss2	3.69 ± 1.27
	ΔR (Å)	dr2	0.03 ± 0.02
	R (Å)		3.57 ± 0.02
Dy-Dy	CN	-	6[a]
	σ^2 (10^{-3} Å^2)	ss3	7.34 ± 2.43
	ΔR (Å)	dr3	0.03 ± 0.03
	R (Å)		4.04 ± 0.03
Dy-O	CN	-	3[a]
	σ^2 (10^{-3} Å^2)	ss3	7.34 ± 2.43
	ΔR (Å)	dr3	0.03 ± 0.03
	R (Å)		4.25 ± 0.03
All Paths	ΔE_o (eV)	dE	2.27 ± 1.60
	S_o^2	-	1.0[a]
Fit Statistics	Independent Points		11.0
	Number of Parameters		7
	Reduced χ^2		1846
	R-factor		0.012
Fit Range	k-range (Å^{-1})		3.65-9.76
	R-range (Å)		1.3-4.2
Fuzziness	Distance Fuzz (Å)		0.10

[a]S_o^2 set to 1.0 and CN set to the value of the structure file during fitting.

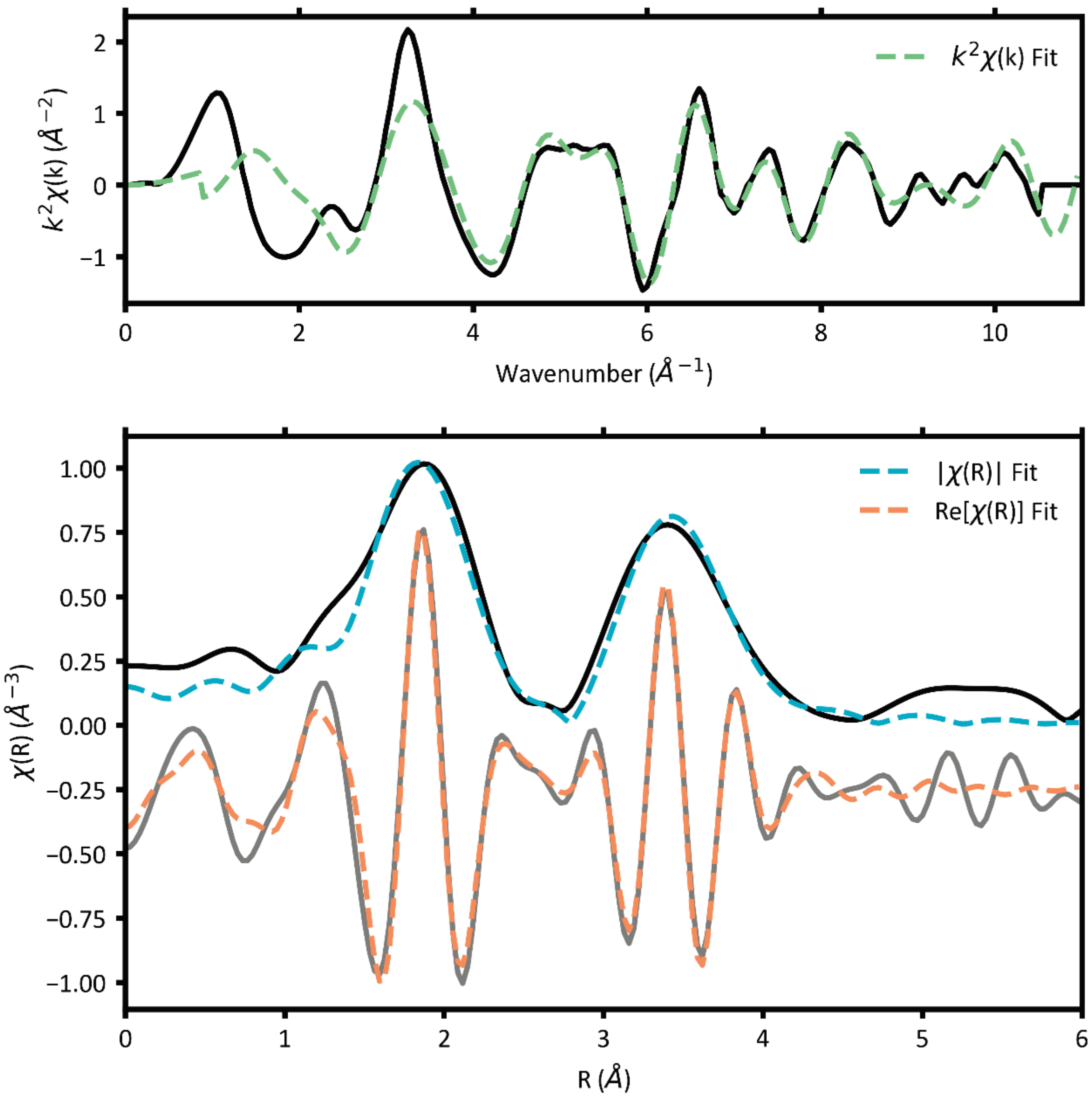

Figure S15 The k^2-weighted k-space (top) and magnitude and real part of the Fourier Transform (bottom) of the EXAFS for the Ho_2O_3 L_2-edge (solid black and gray lines). Fits with k-range 3.7-9.9 Å-1 and R-range 1.5-4.2 Å are represented by the colored dashed lines.

Table S17 Fitting parameters for the L_2-edge EXAFS of Ho_2O_3.

		Varied Parameter	Fit Results
Ho-O	CN	-	5.5[a]
	σ^2 (10^{-3} Å^2)	ss1	5.41 ± 1.04
	ΔR (Å)	dr1	0.01 ± 0.02
	R (Å)		2.28 ± 0.02
Ho-Ho	CN	-	6.0[a]
	σ^2 (10^{-3} Å^2)	ss2	3.85 ± 1.22
	ΔR (Å)	dr2	0.02 ± 0.02
	R (Å)		3.55 ± 0.02
Ho-Ho	CN	-	6.0[a]
	σ^2 (10^{-3} Å^2)	ss3	7.06 ± 2.64
	ΔR (Å)	dr3	0.07 ± 0.03
	R (Å)		4.06 ± 0.03
Ho-O	CN	-	5.0[a]
	σ^2 (10^{-3} Å^2)	ss3	7.06 ± 2.64
	ΔR (Å)	dr3	0.07 ± 0.03
	R (Å)		4.20 ± 0.03
All Paths	ΔE_o (eV)	dE	2.93 ± 2.04
	S_o^2	-	1.0[a]
Fit Statistics	Independent Points		10.5
	Number of Parameters		7
	Reduced χ^2		1631
	R-factor		0.015
Fit Range	k-range (Å^{-1})		3.7 - 9.9
	R-range (Å)		1.5 – 4.2
Fuzziness	Distance Fuzz (Å)		0.10

[a]S_o^2 set to 1.0 and CN set to the value of the structure file during fitting.

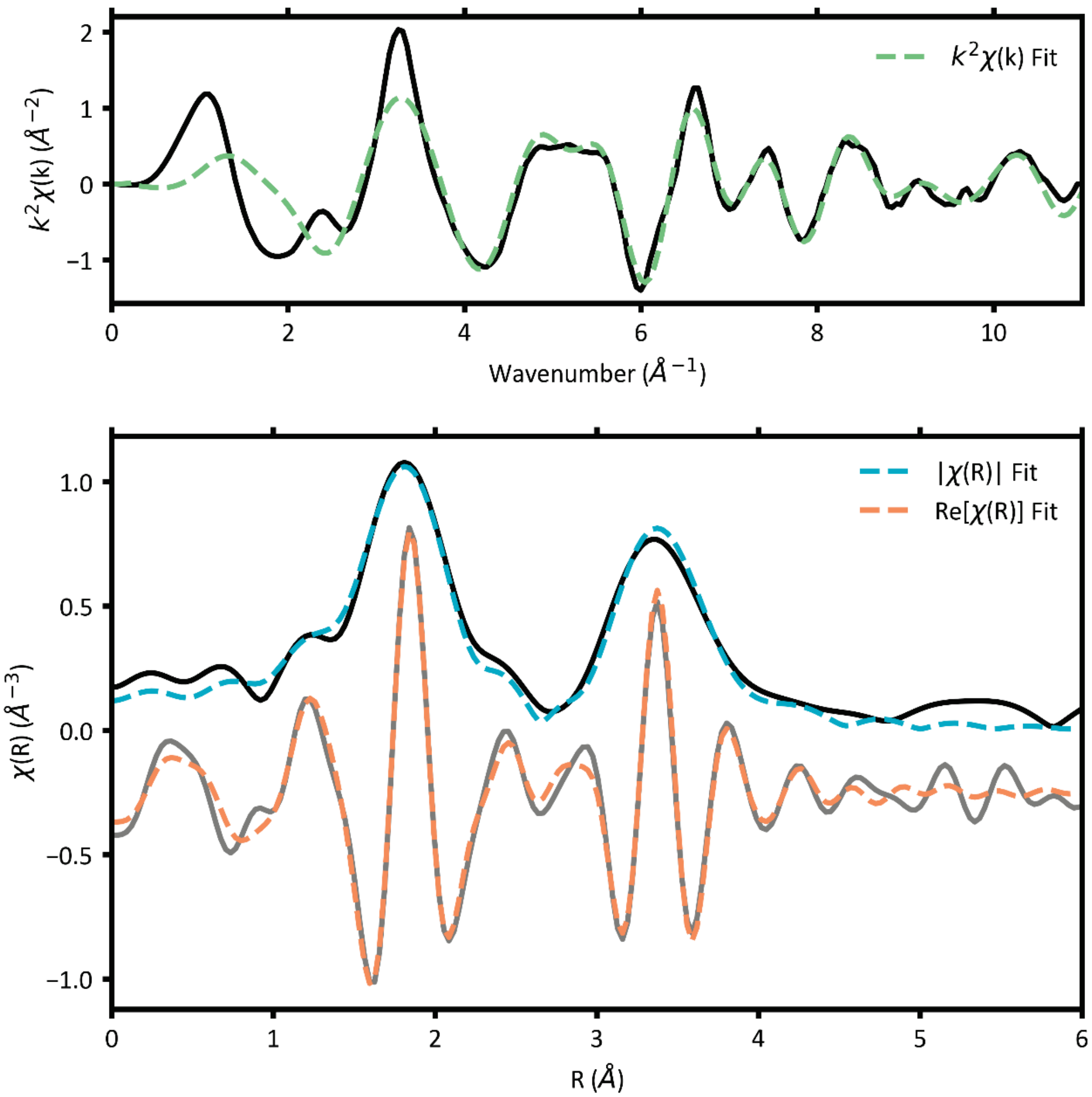

Figure S16 The k^2-weighted k-space (top) and magnitude and real part of the Fourier Transform (bottom) of the EXAFS for the Er_2O_3 L_2-edge (solid black and gray lines). Fits with k-range 3.7-10.64 Å-1 and R-range 1.3-4.2 Å are represented by the colored dashed lines.

Table S18 Fitting parameters for the L_2-edge EXAFS of Er_2O_3.

		Varied Parameter	Fit Results
Er-O	CN	-	6.0 [a]
	σ^2 (10^{-3} Å^2)	ss1	6.89 ± 0.61
	ΔR (Å)	dr1	-0.02 ± 0.01
	R (Å)		2.25 ± 0.01
Er-Er	CN	-	6.0 [a]
	σ^2 (10^{-3} Å^2)	ss2	5.40 ± 0.65
	ΔR (Å)	dr2	0.01 ± 0.01
	R (Å)		3.51 ± 0.01
Er-Er	CN	-	6.0 [a]
	σ^2 (10^{-3} Å^2)	ss3	7.75 ± 1.26
	ΔR (Å)	dr2	0.01 ± 0.01
	R (Å)		4.00 ± 0.01
Er-O	CN	-	3.0 [a]
	σ^2 (10^{-3} Å^2)	ss3	7.75 ± 1.26
	ΔR (Å)	dr2	0.01 ± 0.01
	R (Å)		4.18 ± 0.01
All Paths	ΔE_o (eV)	dE	0.32 ± 0.96
	S_o^2	-	1.0[a]
Fit Statistics	Independent Points		12.4
	Number of Parameters		6
	Reduced χ^2		379.6
	R-factor		0.009
Fit Range	k-range (Å^{-1})		3.7 – 10.64
	R-range (Å)		1.3 – 4.2
Fuzziness	Distance Fuzz (Å)		0.10

[a]S_o^2 set to 1.0 and CN set to the value of the structure file during fitting.

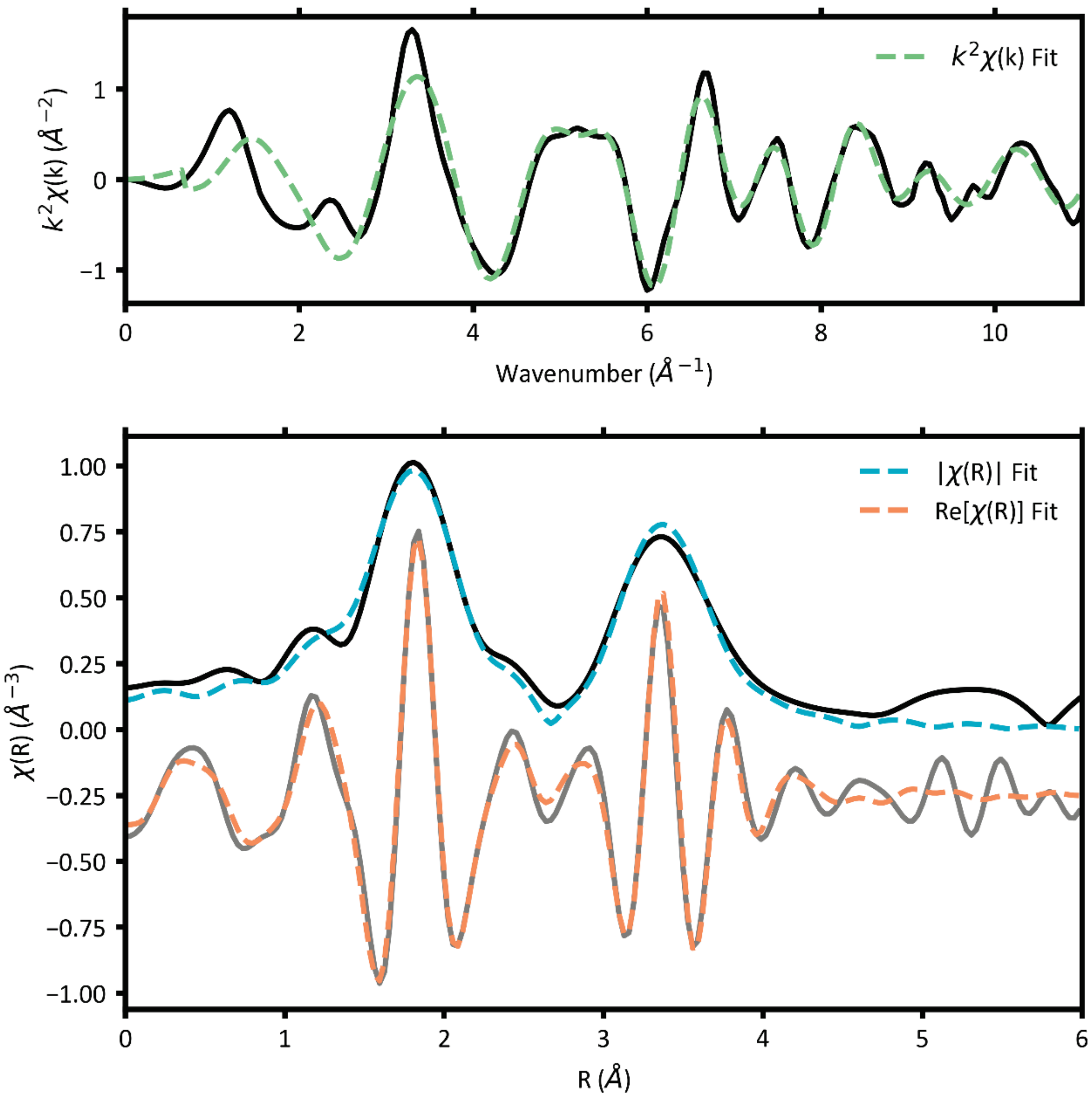

Figure S17 The k^2-weighted k-space (top) and magnitude and real part of the Fourier Transform (bottom) of the EXAFS for the Tm_2O_3 L_3-edge (solid black and gray lines). Fits with k-range 3.7-10.5 Å-1 and R-range 1.3-4.2 Å are represented by the colored dashed lines.

Table S19 Fitting parameters for the L_3-edge EXAFS of Tm_2O_3.

		Varied Parameter	Fit Results
Tm-O	CN	-	6[a]
	σ^2 (10^{-3} Å^2)	ss1	7.88 ± 0.91
	ΔR (Å)	dr1	0.00 ± 0.01
	R (Å)		2.24 ± 0.01
Tm-Tm	CN	-	6[a]
	σ^2 (10^{-3} Å^2)	ss2	4.96 ± 1.08
	ΔR (Å)	dr2	0.01 ± 0.01
	R (Å)		3.51 ± 0.01
Tm-Tm	CN	-	6[a]
	σ^2 (10^{-3} Å^2)	ss3	7.27 ± 2.08
	ΔR (Å)	dr3	0.03 ± 0.03
	R (Å)		3.96 ± 0.03
All Paths	ΔE_o (eV)	dE	1.68 ± 1.55
	S_o^2	-	1.0[a]
Fit Statistics	Independent Points		12.3
	Number of Parameters		7
	Reduced χ^2		1715
	R-factor		0.015
Fit Range	k-range (Å^{-1})		3.7 – 10.5
	R-range (Å)		1.3 – 4.2
Fuzziness	Distance Fuzz (Å)		0.10

[a]S_o^2 set to 1.0 and CN set to the value of the structure file during fitting.

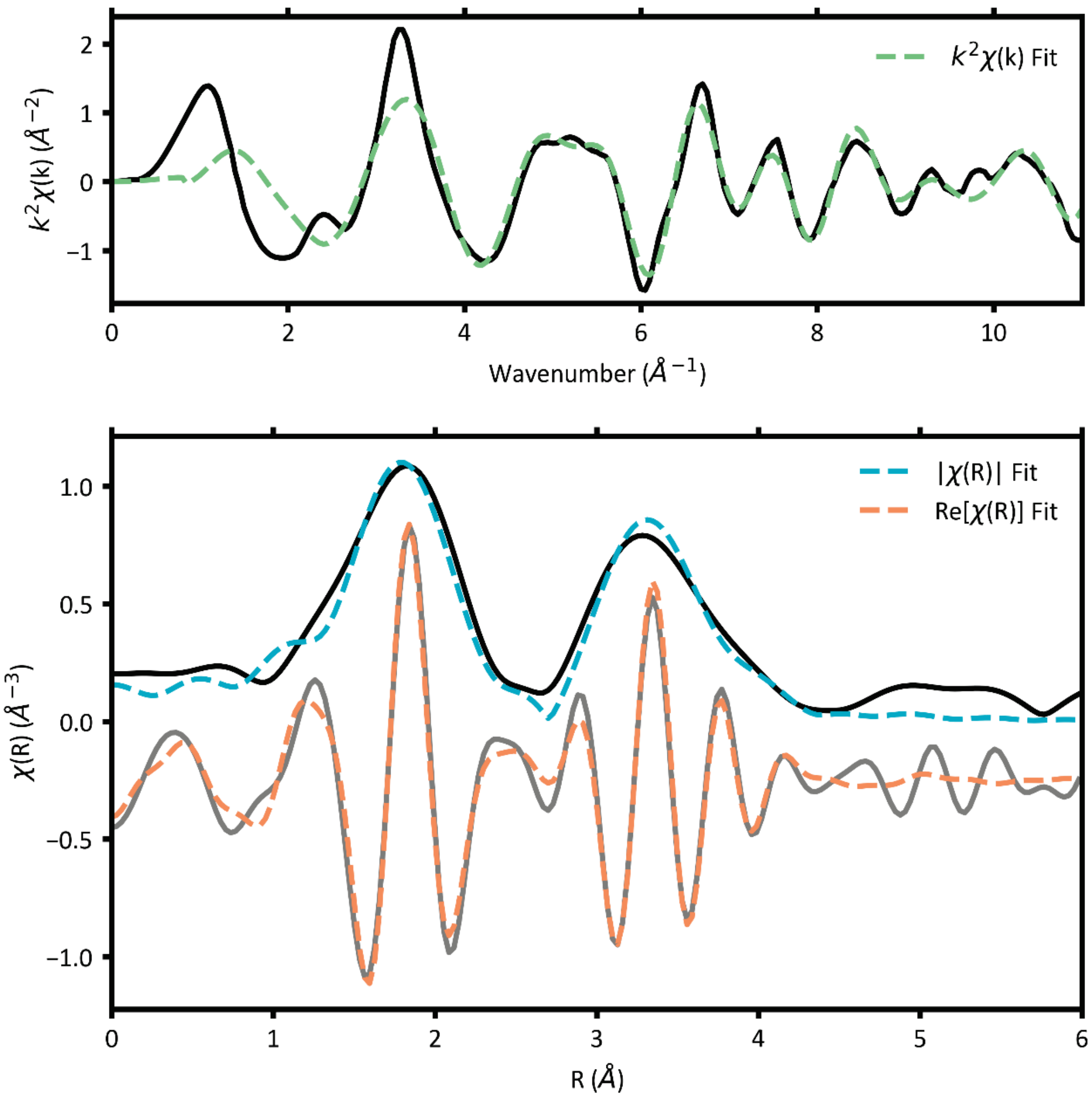

Figure S18 The k^2-weighted k-space (top) and magnitude and real part of the Fourier Transform (bottom) of the EXAFS for the Yb_2O_3 L_2-edge (solid black and gray lines). Fits with k-range 3.65-10.0 Å-1 and R-range 1.3-4.5 Å are represented by the colored dashed lines.

Table S20 Fitting parameters for the L_2-edge EXAFS of Yb_2O_3.

		Varied Parameter	Fit Results
Yb-O	CN	-	6.0[a]
	σ^2 (10^{-3} Å^2)	ss1	6.42 ± 0.98
	ΔR (Å)	dr1	-0.01 ± 0.01
	R (Å)		2.23 ± 0.01
Yb-Yb	CN	-	6.0[a]
	σ^2 (10^{-3} Å^2)	ss2	3.45 ± 1.25
	ΔR (Å)	dr2	0.02 ± 0.02
	R (Å)		3.48 ± 0.02
Yb-Yb	CN	-	6.0[a]
	σ^2 (10^{-3} Å^2)	ss3	5.34 ± 2.21
	ΔR (Å)	dr3	0.01 ± 0.3
	R (Å)		3.96 ± 0.03
All Paths	ΔE_o (eV)	dE	2.52 ± 1.64
	S_o^2	-	1.0[a]
Fit Statistics	Independent Points		12.7
	Number of Parameters		7
	Reduced χ^2		2403
	R-factor		0.019
Fit Range	k-range (Å^{-1})		3.65-10.0
	R-range (Å)		1.3-4.5
Fuzziness	Distance Fuzz (Å)		0.10

[a]S_o^2 set to 1.0 and CN set to the value of the structure file during fitting.

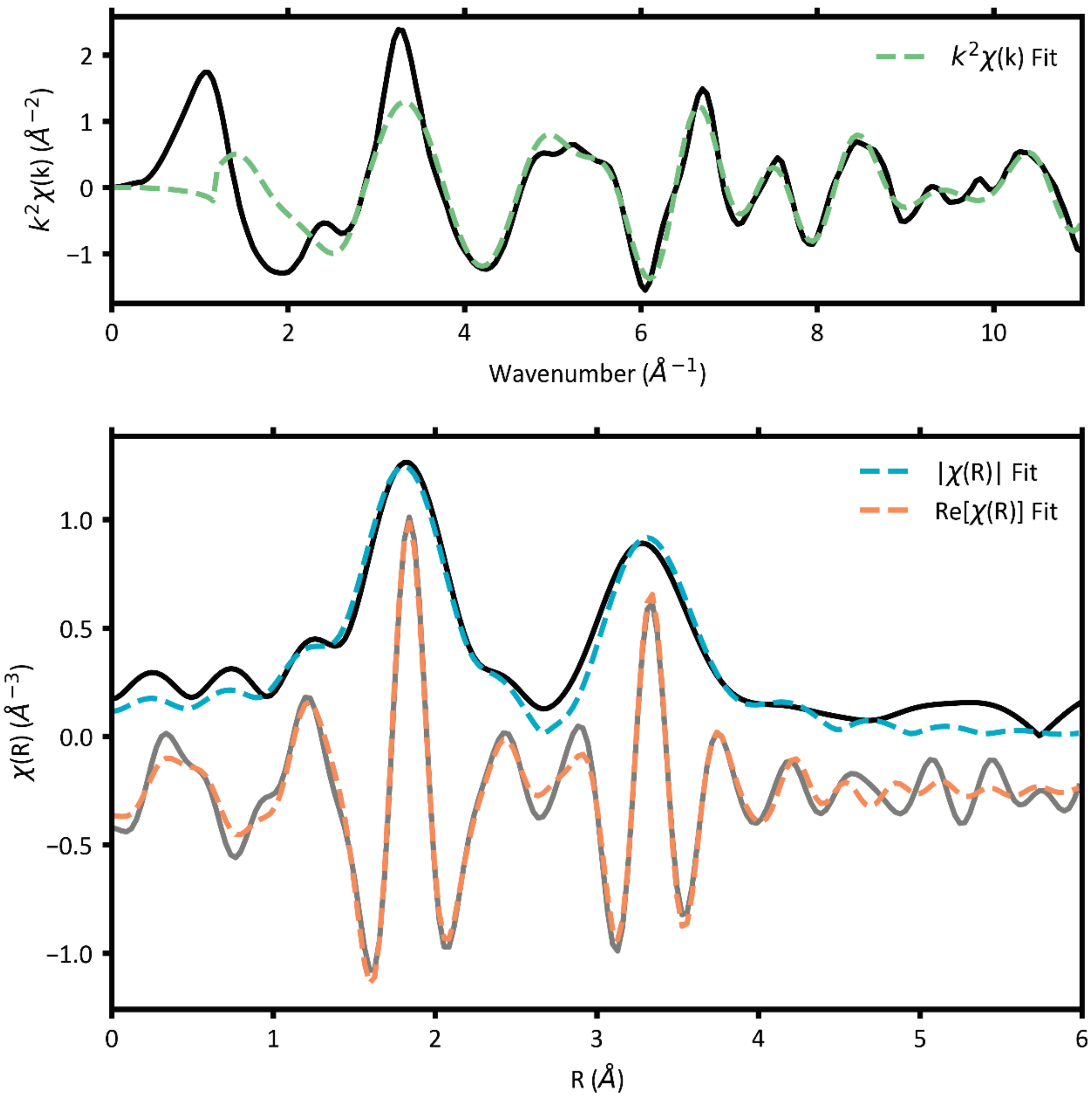

Figure S19 The k^2-weighted k-space (top) and magnitude and real part of the Fourier Transform (bottom) of the EXAFS for the Lu_2O_3 L_2-edge (solid black and gray lines). Fits with k-range 3.71-10.7 Å-1 and R-range 1-4.2 Å are represented by the colored dashed lines.

Table S21 Fitting parameters for the L_2-edge EXAFS of Lu_2O_3.

		Varied Parameter	Fit Results
Lu-O	CN	-	4.5[a]
	σ^2 (10^{-3} Å^2)	ss1	4.47 ± 0.66
	ΔR (Å)	dr1	0.00 ± 0.01
	R (Å)		2.22 ± 0.01
Lu-O	CN	-	1.5[a]
	σ^2 (10^{-3} Å^2)	ss1	4.47 ± 0.66
	ΔR (Å)	dr1	0.00 ± 0.01
	R (Å)		2.30 ± 0.01
Lu-Lu	CN	-	6.0[a]
	σ^2 (10^{-3} Å^2)	ss2	3.23 ± 0.74
	ΔR (Å)	dr2	0.03 ± 0.01
	R (Å)		3.47 ± 0.01
Lu-Lu	CN	-	6.0[a]
	σ^2 (10^{-3} Å^2)	ss3	6.04 ± 1.52
	ΔR (Å)	dr3	0.04 ± 0.02
	R (Å)		3.96 ± 0.02
Lu-O	CN	-	3.0[a]
	σ^2 (10^{-3} Å^2)	ss3	6.04 ± 1.52
	ΔR (Å)	dr3	0.04 ± 0.02
	R (Å)		4.13 ± 0.02
All Paths	ΔE_o (eV)	dE	5.04 ± 1.17
	S_o^2	-	1.0[a]
Fit Statistics	Independent Points		14.0
	Number of Parameters		7
	Reduced χ^2		1862
	R-factor		0.013
Fit Range	k-range (Å^{-1})		3.71-10.7
	R-range (Å)		1-4.2
Fuzziness	Distance Fuzz (Å)		0.10

[a]S_o^2 set to 1.0 and CN set to the value of the structure file during fitting.

S3. Powder X-ray Diffraction (XRD) Patterns Confirming Phase

Powder X-ray diffraction (XRD) was used to confirm the phase of the lanthanide oxide samples helping guide the XAS analysis. Diffraction patterns were collected on a lab source diffractometer or using beamline 2-1 at the Stanford Synchrotron Radiation Lightsource. For the lab source diffractometer, samples were front-loaded into a silicon zero background holder and diffraction data were collected from 15 to 70° 2θ using a Malvern Panalytical Empyrean® instrument fitted with a copper (λ = 1.5406 Å) long-fine-focus X-ray tube operated at 45 kV and 40 mA. The incident beam path included iCore® optics fitted with a BBHD® optic with 0.03 radian Soller slits, a 14 mm primary and a 14 mm secondary mask, and a fixed 1/4° divergence slit. The diffracted beam path incorporated dCore® optics with a 1/4° fixed anti-scatter slit, and 0.04 radian Soller slits. A PIXcel3D® detector was used in scanning line (1D) mode with an active length of 3.347° 2θ. Data were collected with a nominal step size of 0.0263° 2θ for 96.39 seconds for a total scan time of 15 minutes. For the synchrotron diffraction data, beamline 2-1 was operated at 17,000 eV (λ = 0.73 Å) with the energy selected by using a Si (111) monochromator and the diffraction patterns collected using a Pilatus 100K area detector.

XRD analysis consisted of comparing Bragg peak positions to those in literature data from the International Centre for Diffraction Data (ICDD) PDF4® database using Jade® software (version 8.7) from Materials Data Inc. (MDI). The CIF files from the PDF4® database were used as structure files to create theoretical scattering paths used in the EXAFS fitting. All collected lanthanide oxide diffraction patterns (listed in Table S23) were good qualitative matches to structures in the PDF4® database (Figures S20-S33). There are some peaks from minor phases and impurities in some of the samples (Figs. S20, S22, S23). However, these peaks are quite few and low intensity compared to the identified peaks, suggesting that these phases/impurities are very minor. This is further supported by the EXAFS results (Fig. 6, Figs. S2-4, S8-9 and Tables S5-7, S9-12) that closely match what is expected of the primary phase. Of these samples with minor phases/impurities, Pr showed the largest deviation from the expected XRD structure, but a satisfactory EXAFS fit was still achieved (R-factor = 0.02) and is discussed in Section 3.4 of the main text with more detailed results in the S2.

Table S22 Lanthanide oxide structure files used in the XRD phase confirmation and subsequent EXAFS fitting.

Lanthanide Oxide	PDF4 Database Code
La_2O_3	00-005-0602
CeO_2	01-090-3051[a]<br>01-075-0223 (diamond)[a, c]
Pr_2O_3	04-008-6652 (major phase: cubic)<br>04-008-8161 (minor phase: hexagonal)[b]
Nd_2O_3	04-007-5405[a]<br>01-075-0223 (diamond)[a, c]
Sm_2O_3	00-015-0813
Eu_2O_3	00-034-0392
Gd_2O_3	00-012-0797
Tb_2O_3	01-086-2478
Dy_2O_3	01-081-8691
Ho_2O_3	01-074-1829
Er_2O_3	01-070-8740
Tm_2O_3	01-078-0389
Yb_2O_3	01-074-1831
Lu_2O_3	01-085-7630

[a]To compare synchrotron XRD to benchtop XRD data, we used Bragg's law. The synchrotron data was gathered at an energy of 17,000 eV and wavelength of 0.73 Å, while the benchtop data used copper k-α X-rays (1.5406 Å).

[b]Not used in EXAFS fitting. Only used in phase confirmation of a minor phase by XRD

[c]Used to account for diamond peaks in XRD done using synchrotron x-rays at the SLAC National Accelerator Laboratory

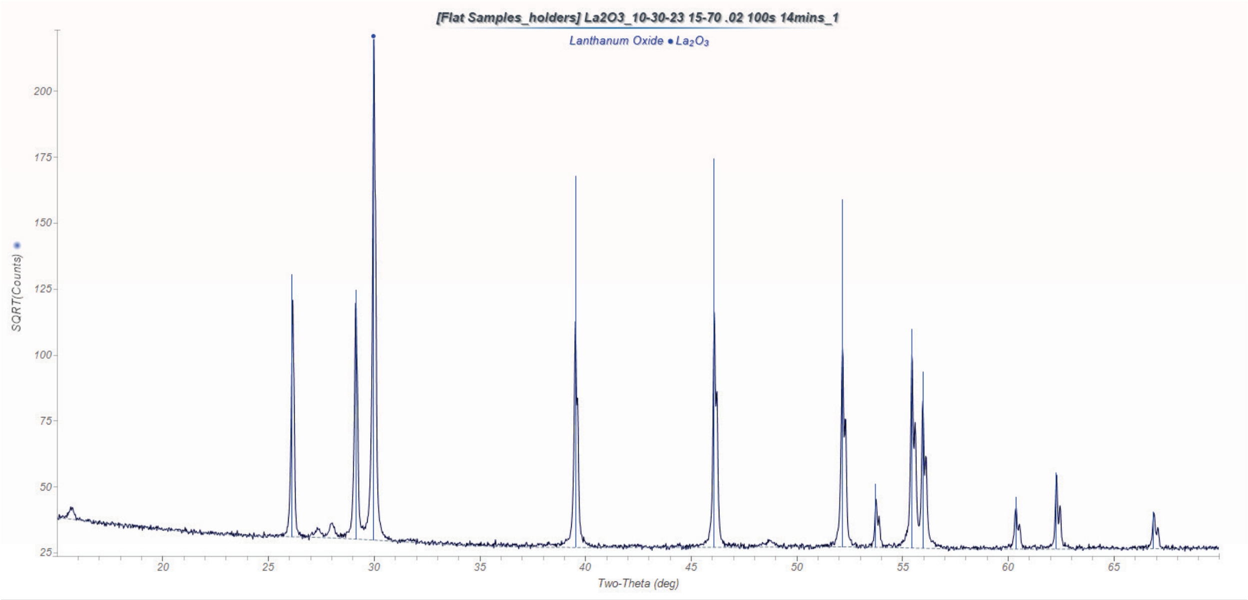

Figure S20 The XRD diffraction pattern for the calcined La_2O_3 used in the XAS data collection. The blue sticks are the relative intensity and Two-Theta angle of a previously refined structure from the PDF4® database.

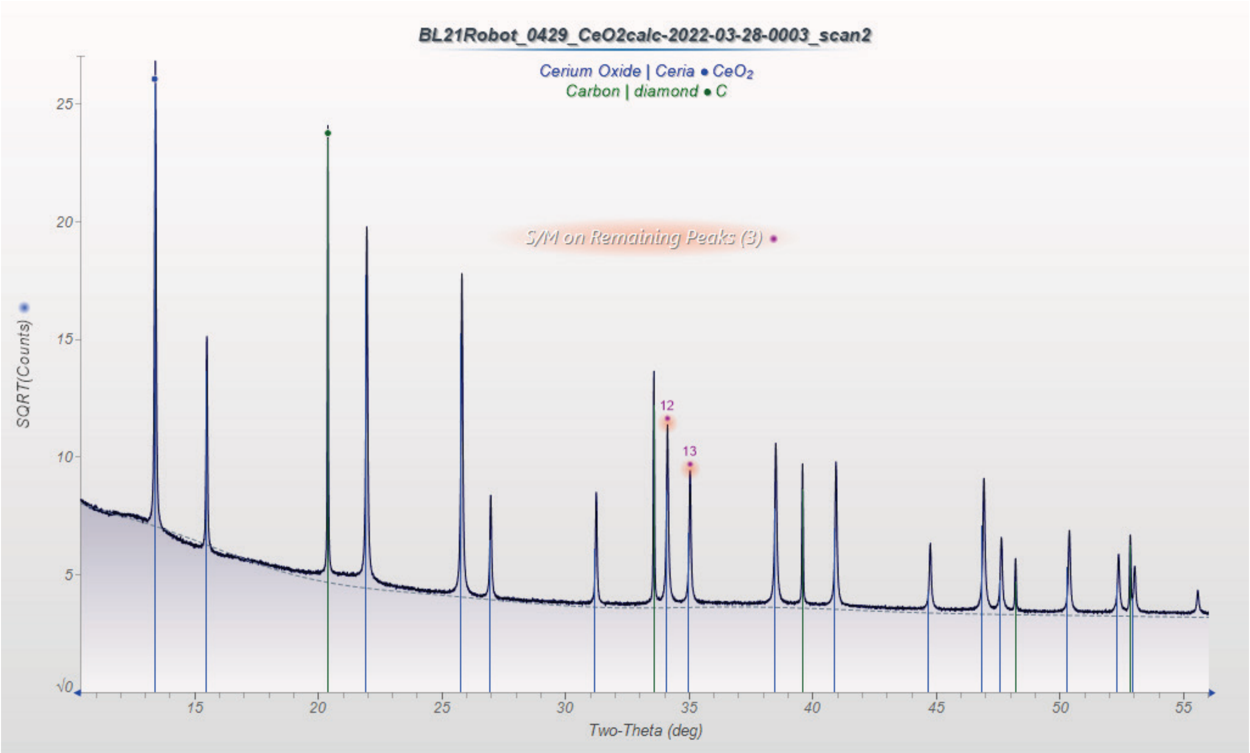

Figure S21 The XRD diffraction pattern (collected using synchrotron X-rays) for the calcined CeO_2 used in the XAS data collection. The blue sticks are the relative intensity and Two-Theta angle of a previously refined structure from the PDF4® database. Diamond was added to achieve an appropriate sample absorption given the capillary geometry for the synchrotron measurements.

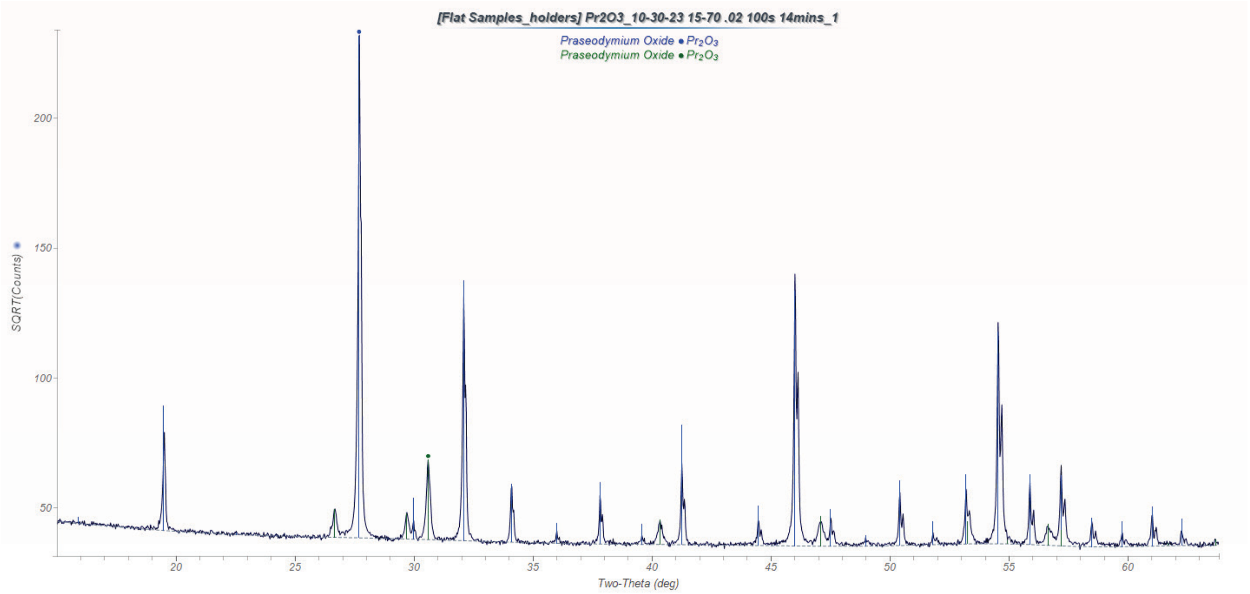

Figure S22 The XRD diffraction pattern for the calcined Pr_2O_3 used in the XAS data collection. The blue sticks are the relative intensity and Two-Theta angle of a previously refined structure from the PDF4® database.

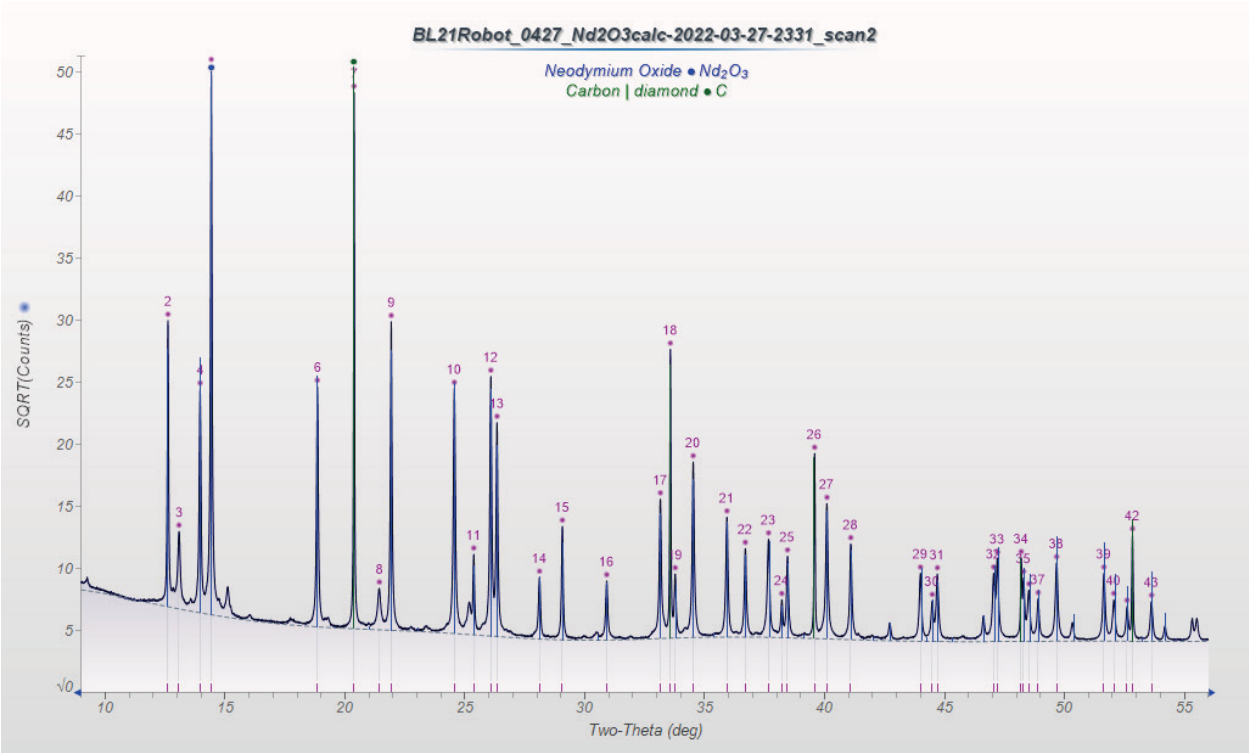

Figure S23 The XRD diffraction pattern (collected using synchrotron X-rays) for the calcined Nd_2O_3 used in the XAS data collection. The blue sticks are the relative intensity and Two-Theta angle of a previously refined structure from the PDF4® database. Diamond was added to achieve an appropriate sample absorption given the capillary geometry for the synchrotron measurements.

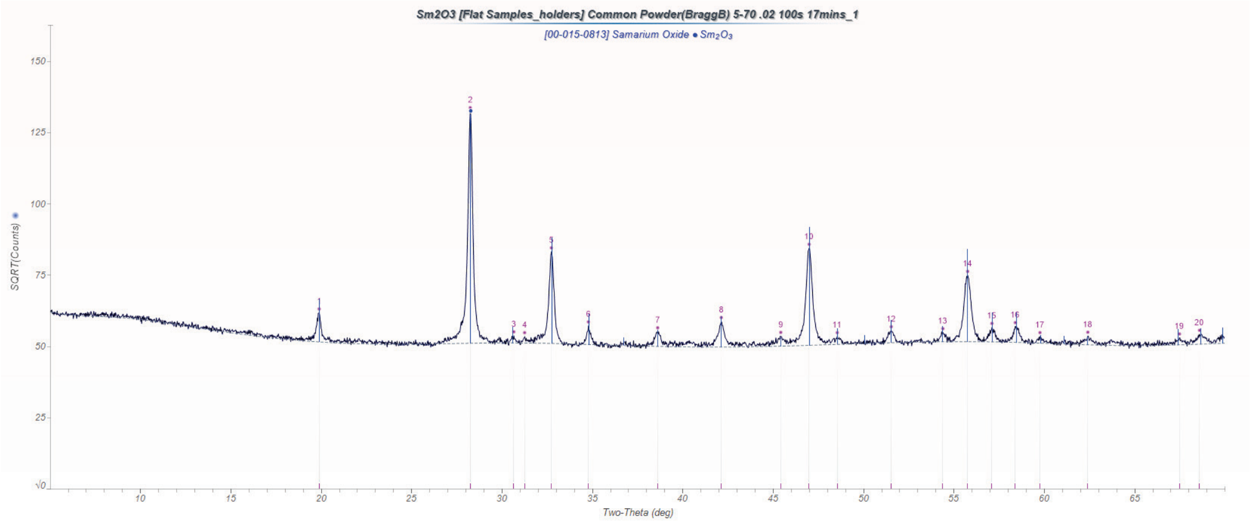

Figure S24 The XRD diffraction pattern for the calcined Sm_2O_3 used in the XAS data collection. The blue sticks are the relative intensity and Two-Theta angle of a previously refined structure from the PDF4® database.

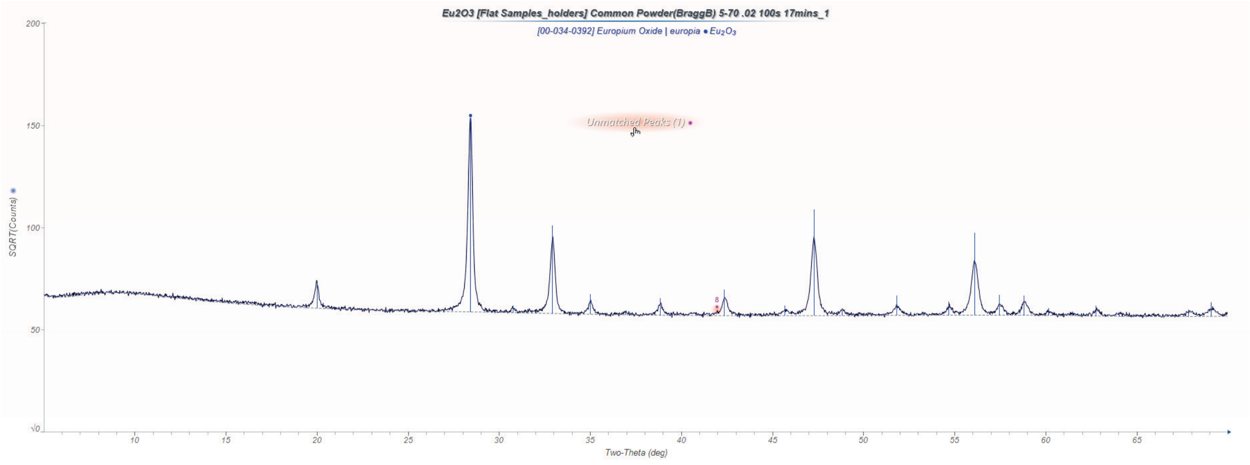

Figure S25 The XRD diffraction pattern for the calcined Eu_2O_3 used in the XAS data collection. The blue sticks are the relative intensity and Two-Theta angle of a previously refined structure from the PDF4® database.

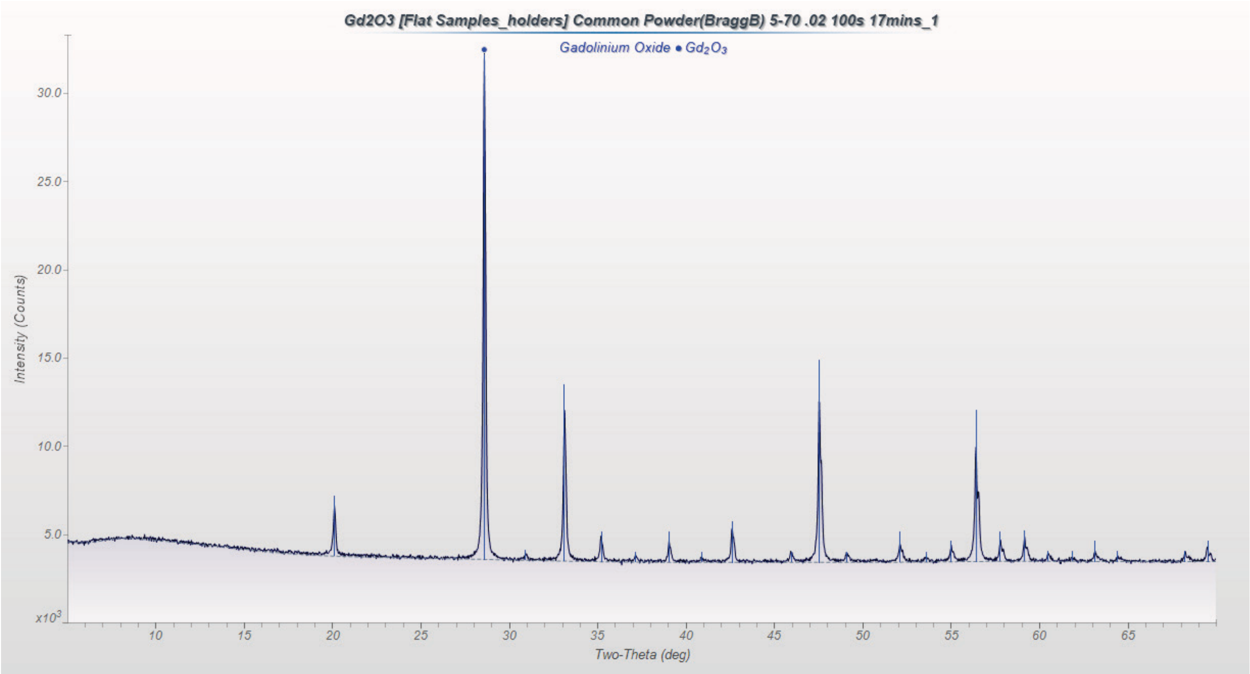

Figure S26 The XRD diffraction pattern for the calcined Gd_2O_3 used in the XAS data collection. The blue sticks are the relative intensity and Two-Theta angle of a previously refined structure from the PDF4® database.

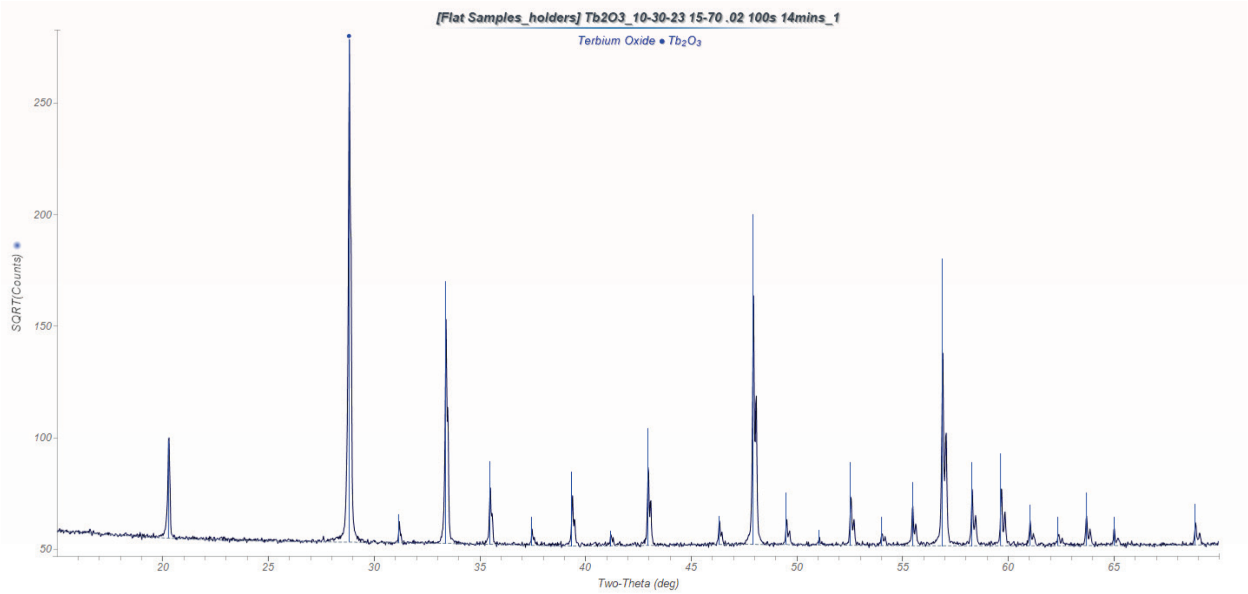

Figure S27 The XRD diffraction pattern for the calcined Tb_2O_3 used in the XAS data collection. The blue sticks are the relative intensity and Two-Theta angle of a previously refined structure from the PDF4® database.

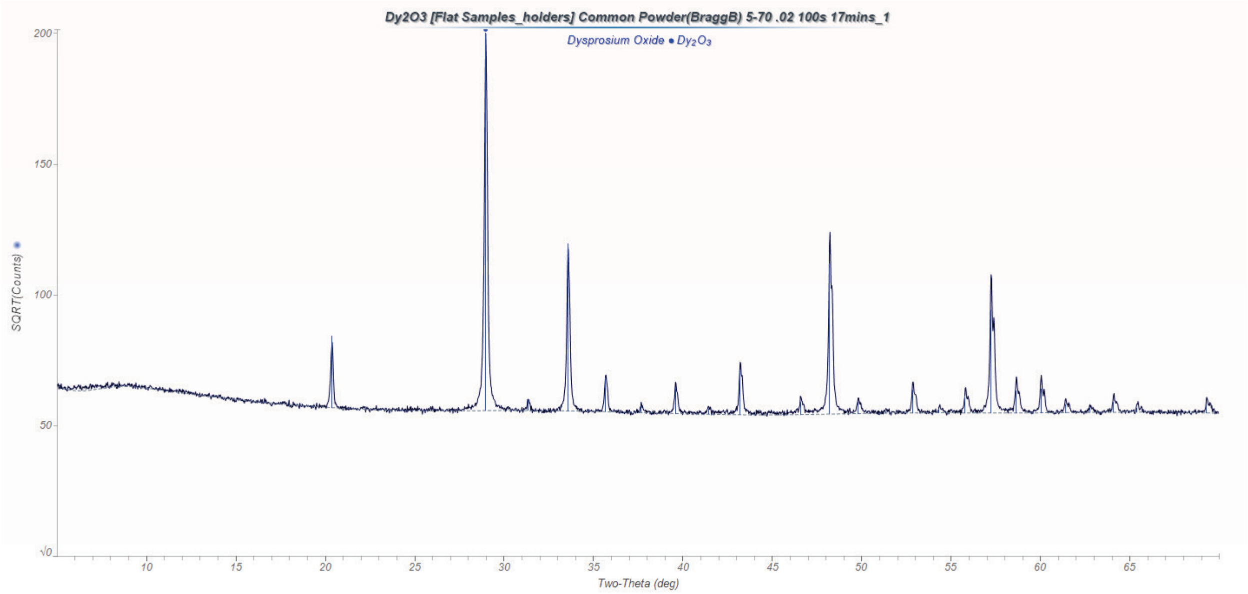

Figure S28 The XRD diffraction pattern for the calcined Dy_2O_3 used in the XAS data collection. The blue sticks are the relative intensity and Two-Theta angle of a previously refined structure from the PDF4® database.

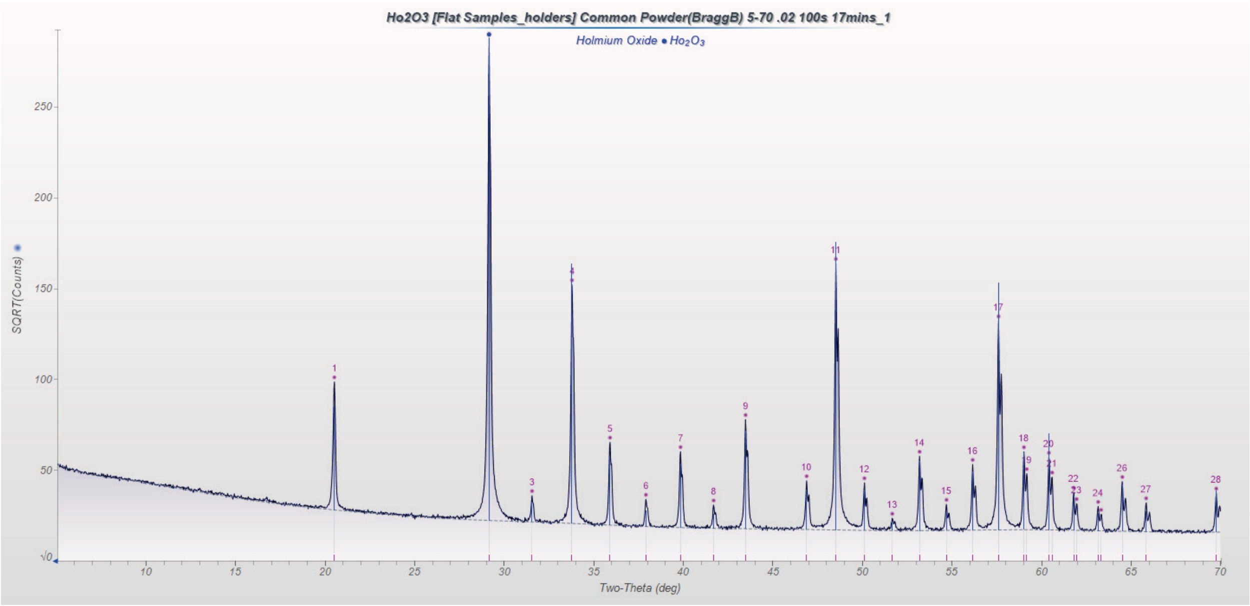

Figure S29 The XRD diffraction pattern for the calcined Ho_2O_3 used in the XAS data collection. The blue sticks are the relative intensity and Two-Theta angle of a previously refined structure from the PDF4® database.

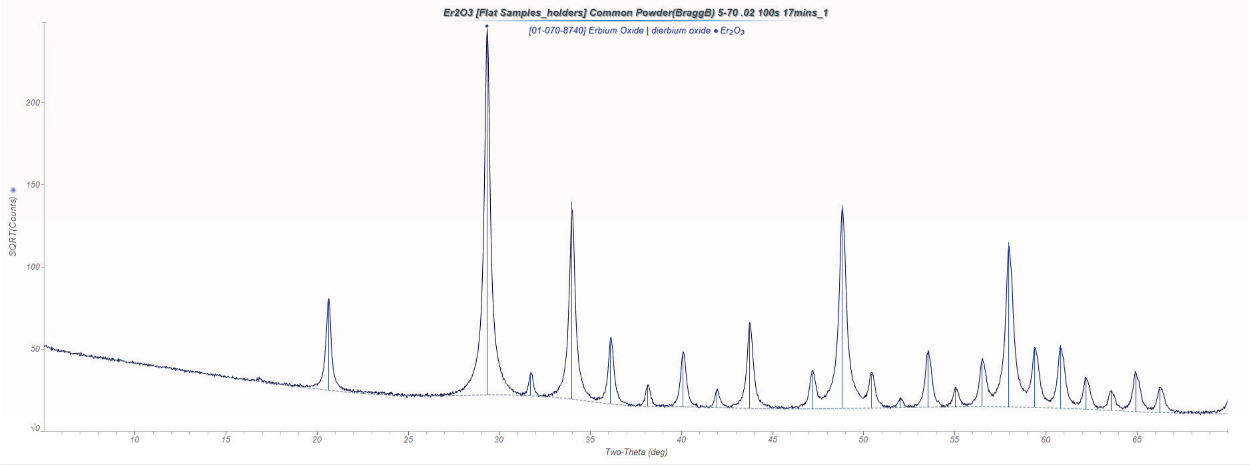

Figure S30 The XRD diffraction pattern for the calcined Er_2O_3 used in the XAS data collection. The blue sticks are the relative intensity and Two-Theta angle of a previously refined structure from the PDF4® database.

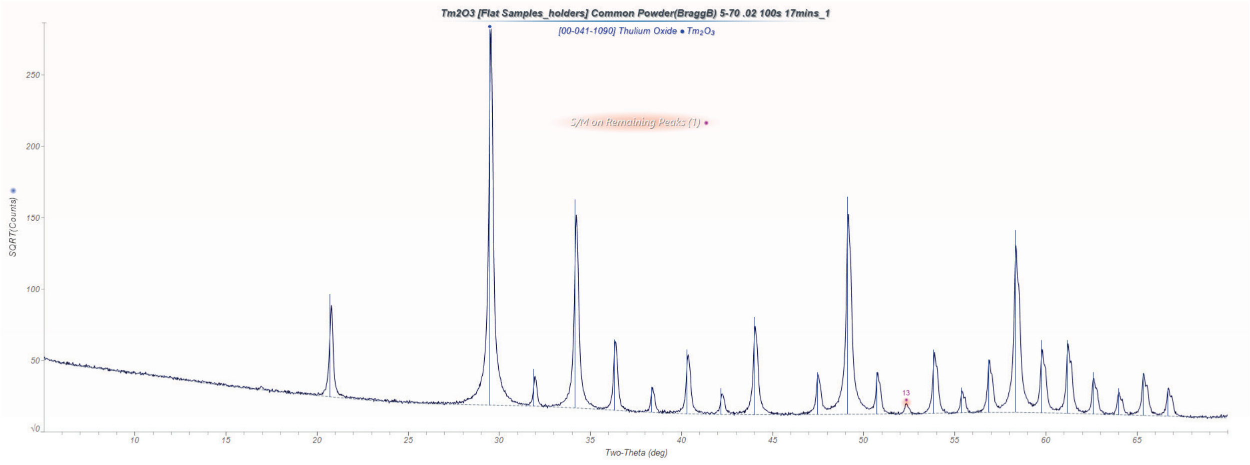

Figure S31 The XRD diffraction pattern for the calcined Tm_2O_3 used in the XAS data collection. The blue sticks are the relative intensity and Two-Theta angle of a previously refined structure from the PDF4® database.

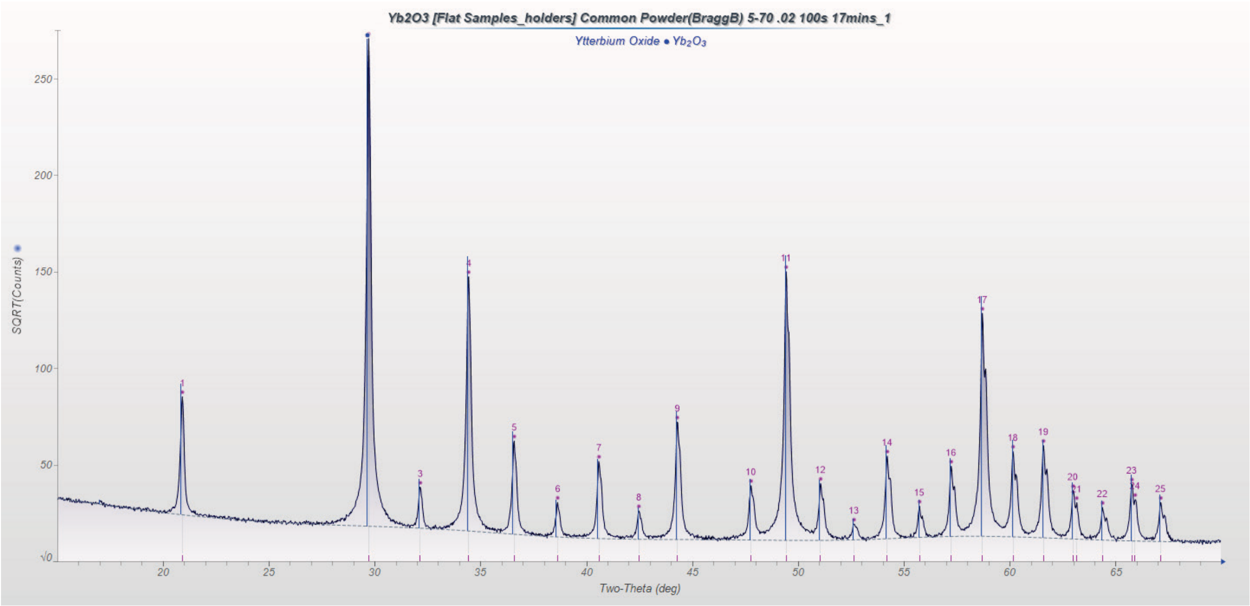

Figure S32 The XRD diffraction pattern for the calcined Yb_2O_3 used in the XAS data collection. The blue sticks are the relative intensity and Two-Theta angle of a previously refined structure from the PDF4® database.

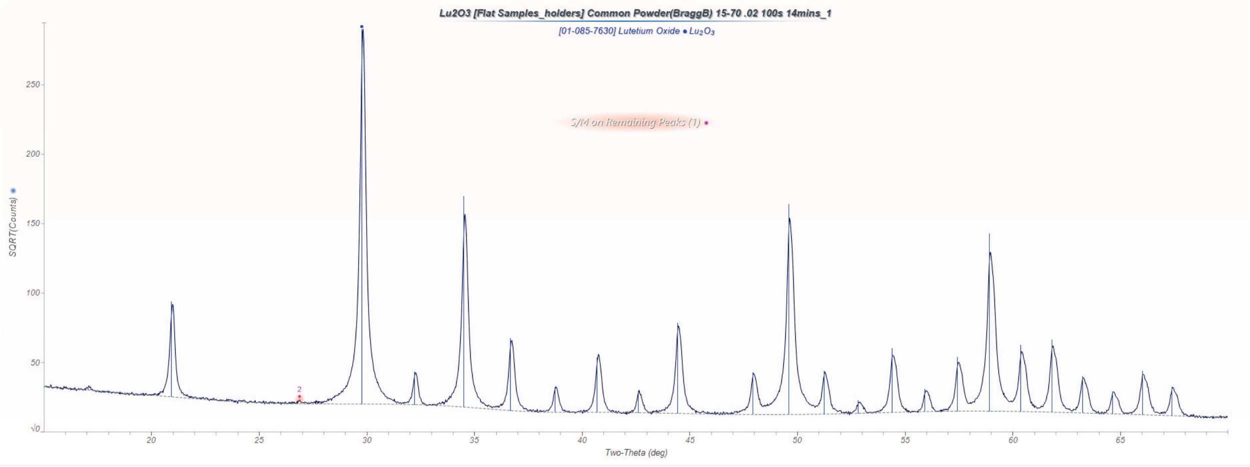

Figure S33 The XRD diffraction pattern for the calcined Lu_2O_3 used in the XAS data collection. The blue sticks are the relative intensity and Two-Theta angle of a previously refined structure from the PDF4® database.

References

Abdusalyamova, M. N., Makhmudov, F. A., Shairmardanov, E. N., Kovalev, I. D., Fursikov, P. V., Khodos, I. I. & Shulga, Y. M. (2014). *Journal of Alloys and Compounds* **601**, 31–37.

Abu-Zied, B. M. & Asiri, A. M. (2014). *Journal of Rare Earths* **32**, 259–264.

Abu-Zied, B. M., Hussein, M. A. & Asiri, A. M. (2016). *Int. J. Electrochem. Sci.* **11**, 7182–7197.

An, L., Zhang, J., Liu, M. & Wang, S. (2008). *Journal of Alloys and Compounds* **451**, 538–541.

Azad, F. & Maqsood, A. (2014). *Electron. Mater. Lett.* **10**, 557–563.

Calvin, S. (2013). XAFS for Everyone Boca Raton: CRC Press.

Chen, F., Zhang, X. H., Hu, X. D., Zhang, W., Zeng, R., Liu, P. D. & Zhang, H. Q. (2016). *Journal of Alloys and Compounds* **664**, 311–316.

Chen, J.-C., Chen, W.-C., Tien, Y.-C. & Shih, C.-J. (2010). *Journal of Alloys and Compounds* **496**, 364–369.

Curtis, C. E. & Tharp, A. G. (1959). *Journal of the American Ceramic Society* **42**, 151–156.

Djuričić, B. & Pickering, S. (1999). *Journal of the European Ceramic Society* **19**, 1925–1934.

Duhan, S., Aghamkar, P. & Singh, M. (2008). *Physics Research International* **2008**, e237023.

Ekthammathat, N., Phuruangrat, A., Kuntalue, B., Thongtem, S. & Thongtem, T. (2015). *Dig. J. Nanomater. Biostructures* **10**, 715–719.

El Desouky, F. G., Saadeldin, M. M., Mahdy, M. A., Wahab, S. M. A. E. & El Zawawi, I. K. (2020). *Materials Science in Semiconductor Processing* **111**, 104991.

Farahmandjou, M., Zarinkamar, M., Firoozabadi, T. P., Farahmandjou, M., Zarinkamar, M. & Firoozabadi, T. P. (2016). *Revista Mexicana de Física* **62**, 496–499.

Gao, J., Zhao, Y., Yang, W., Tian, J., Guan, F., Ma, Y., Hou, J., Kang, J. & Wang, Y. (2003). *Materials Chemistry and Physics* **77**, 65–69.

Ghiasi, M. & Malekzadeh, A. (2015). *Superlattices and Microstructures* **77**, 295–304.

Ghosh, D., Choudhury, N., Balaji, S., Dana, K. & Dhar, A. (2021). *J Mater Sci: Mater Electron* **32**, 4505–4514.

Hosokawa, S., Iwamoto, S. & Inoue, M. (2007). *Journal of the American Ceramic Society* **90**, 1215–1221.

Hussein, G. A. M. (2001). *Powder Technology* **118**, 285–290.

Hussein, G. A. M., Buttrey, D. J., DeSanto, P., Abd-Elgaber, A. A., Roshdy, H. & Myhoub, A. Y. Z. (2003). *Thermochimica Acta* **402**, 27–36.

Kirk, N. B. & Wood, J. V. (1995). *JOURNAL OF MATERIALS SCIENCE* **30**, 2171–2175.

Kumar, S., Prakash, R. & Singh, V. K. (2015). *Reviews in Advanced Sciences and Engineering* **4**, 247–257.

Lee, S. W., Park, S. K., Min, B.-K., Kang, J.-G. & Sohn, Y. (2014). *Applied Surface Science* **307**, 736–743.

Leoni, M., Di Maggio, R., Polizzi, S. & Scardi, P. (2004). *Journal of the American Ceramic Society* **87**, 1133–1140.

Li, J.-G., Ikegami, T. & Mori, T. (2004). *Acta Materialia* **52**, 2221–2228.

Li, L., Lian, S., Tang, J., Chen, S., Guo, R., Pan, S. & Peng, C. (2022). *Journal of Colloid and Interface Science* **615**, 69–78.

Mekhemer, G. A. H. (2004). *Applied Catalysis A: General* **275**, 1–7.

M. Hussein, G. A., H. Mekhemer, G. A. & A. Balboul, B. A. (2000). *Physical Chemistry Chemical Physics* **2**, 2033–2038.

Mortazavi-Derazkola, S., Zinatloo-Ajabshir, S. & Salavati-Niasari, M. (2015). *Ceramics International* **41**, 9593–9601.

Nachimuthu, P., Shih, W.-C., Liu, R.-S., Jang, L.-Y. & Chen, J.-M. (2000). *Journal of Solid State Chemistry* **149**, 408–413.

Neumann, A. & Walter, D. (2006). *Thermochimica Acta* **445**, 200–204.

Panitz, J.-C. (1999). *Journal of Raman Spectroscopy* **30**, 1035–1042.

Panitz, J.-C., Schubnell, M., Durisch, W. & Geiger, F. (1997). *AIP Conference Proceedings* **401**, 265–276.

Rahimi-Nasrabadi, M., Pourmortazavi, S. M., Aghazadeh, M., Ganjali, M. R., Karimi, M. S. & Norouzi, P. (2017). *J Mater Sci: Mater Electron* **28**, 9478–9488.

Rahimi-Nasrabadi, M., Pourmortazavi, S. M., Aghazadeh, M., Ganjali, M. R., Sadeghpour Karimi, M. & Novrouzi, P. (2017). *J Mater Sci: Mater Electron* **28**, 5574–5583.

Rahimi-Nasrabadi, M., Pourmortazavi, S. M., Ganjali, M. R., Novrouzi, P., Faridbod, F. & Karimi, M. S. (2017). *J Mater Sci: Mater Electron* **28**, 3325–3336.

Rahimi-Nasrabadi, M., Pourmortazavi, S. M., Sadeghpour Karimi, M., Aghazadeh, M., Ganjali, M. R. & Norouzi, P. (2017). *J Mater Sci: Mater Electron* **28**, 13267–13277.

Riva, F., Martin, T., Douissard, P. A. & Dujardin, C. (2016). *J. Inst.* **11**, C10010.

Rosid, S. J. M., Toemen, S., Wan Abu Bakar, W. A., Zamani, A. H. & Wan Mokhtar, W. N. A. (2019). *Journal of Saudi Chemical Society* **23**, 284–293.

Rudraswamy, B. & Dhananjaya, N. (2012). *IOP Conf. Ser.: Mater. Sci. Eng.* **40**, 012034.

Salavati-Niasari, M., Javidi, J. & Davar, F. (2010). *Ultrasonics Sonochemistry* **17**, 870–877.

Seo, C.-W., Kyung Cha, B., Jeon, S., Kyung Kim, R. & Huh, Y. (2013). *Nuclear Instruments and Methods in Physics Research Section A: Accelerators, Spectrometers, Detectors and Associated Equipment* **699**, 129–133.

Sidorowicz, A., Wajler, A., Węglarz, H., Jach, K., Orliński, K. & Olszyna, A. (2016). *International Journal of Applied Ceramic Technology* **13**, 302–307.

Sirotinkin, V. P., Podzorova, L. I., Mikhailina, N. A. & Pen'kova, O. I. (2022). *Crystallogr. Rep.* **67**, 278–285.

Squire, G. D., Luc, H. & Puxley, D. C. (1994). *Applied Catalysis A: General* **108**, 261–278.

Sunding, M. F., Hadidi, K., Diplas, S., Løvvik, O. M., Norby, T. E. & Gunnæs, A. E. (2011). *Journal of Electron Spectroscopy and Related Phenomena* **184**, 399–409.

Tok, H. A. I. Y., Boey, F. Y. C., Huebner, R. & Ng, S. H. (2006). *J Electroceram* **17**, 75–78.

Tsuzuki, T., Pirault, E. & McCormick, P. G. (1999). *Nanostructured Materials* **11**, 125–131.

Watcharapasorn, A., Jiansirisomboon, S. & Tunkasiri, T. (2008). *J Electroceram* **21**, 613–616.

Whba, F., Mohamed, F., Md Rosli, N. R. A., Abdul Rahman, I. & Idris, M. I. (2021). *Radiation Physics and Chemistry* **179**, 109212.

Yang, H., Zhang, D., Shi, L. & Fang, J. (2008). *Acta Materialia* **56**, 955–967.

Zawadzki, M. & Kępiński, L. (2004). *Journal of Alloys and Compounds* **380**, 255–259.

Zhang, Z., Guo, D., Yang, X. & Zhang, J. (2020). *Materials Letters* **261**, 126866.

Zhao, Q., Guo, N., Jia, Y., Lv, W., Shao, B., Jiao, M. & You, H. (2013). *Journal of Colloid and Interface Science* **394**, 216–222.

Zinatloo-Ajabshir, S., Mortazavi-Derazkola, S. & Salavati-Niasari, M. (2017). *Journal of Molecular Liquids* **231**, 306–313.
